# Multiple Transmitter Receptors in Regions and Layers of the Human Cerebral Cortex

**DOI:** 10.3389/fnana.2017.00078

**Published:** 2017-09-20

**Authors:** Karl Zilles, Nicola Palomero-Gallagher

**Affiliations:** ^1^Research Centre Jülich, Institute of Neuroscience and Medicine (INM-1) Jülich, Germany; ^2^Department of Psychiatry, Psychotherapy, and Psychosomatics, Medical Faculty, RWTH Aachen, and JARA—Translational Brain Medicine Aachen, Germany

**Keywords:** visual cortex, ventral stream, dorsal stream, somatosensory cortex, supragranular layers, granular layer, infragranular layers, multimodal association cortex

## Abstract

We measured the densities (fmol/mg protein) of 15 different receptors of various transmitter systems in the supragranular, granular and infragranular strata of 44 areas of visual, somatosensory, auditory and multimodal association systems of the human cerebral cortex. Receptor densities were obtained after labeling of the receptors using quantitative *in vitro* receptor autoradiography in human postmortem brains. The mean density of each receptor type over all cortical layers and of each of the three major strata varies between cortical regions. In a *single* cortical area, the multi-receptor fingerprints of its strata (i.e., polar plots, each visualizing the densities of multiple *different* receptor types in supragranular, granular or infragranular layers of the *same* cortical area) differ in shape and size indicating regional and laminar specific balances between the receptors. Furthermore, the three strata are clearly segregated into well definable clusters by their receptor fingerprints. Fingerprints of *different* cortical areas systematically vary between functional networks, and with the hierarchical levels within sensory systems. Primary sensory areas are clearly separated from all other cortical areas particularly by their very high muscarinic M_2_ and nicotinic α_4_β_2_ receptor densities, and to a lesser degree also by noradrenergic α_2_ and serotonergic 5-HT_2_ receptors. Early visual areas of the dorsal and ventral streams are segregated by their multi-receptor fingerprints. The results are discussed on the background of functional segregation, cortical hierarchies, microstructural types, and the horizontal (layers) and vertical (columns) organization in the cerebral cortex. We conclude that a cortical column is composed of segments, which can be assigned to the cortical strata. The segments differ by their patterns of multi-receptor balances, indicating different layer-specific signal processing mechanisms. Additionally, *the differences between the strata-and area-specific fingerprints* of the 44 areas reflect the segregation of the cerebral cortex into functionally and topographically definable groups of cortical areas (visual, auditory, somatosensory, limbic, motor), and reveals their hierarchical position (primary and unimodal (early) sensory to higher sensory and finally to multimodal association areas).

**Highlights**

- Densities of transmitter receptors vary between areas of human cerebral cortex.
- Multi-receptor fingerprints segregate cortical layers.
- The densities of all examined receptor types together reach highest values in the supragranular stratum of all areas.
- The lowest values are found in the infragranular stratum.
- Multi-receptor fingerprints of entire areas and their layers segregate functional systems
- Cortical types (primary sensory, motor, multimodal association) differ in their receptor fingerprints.

## Introduction

Cortical layers—as defined in classical architectonic studies (Brodmann, 1909; von Economo and Koskinas, 1925)—differ by cell types (Markram et al., 2004; Xu and Callaway, 2009; DeFelipe et al., 2013; Jiang et al., 2015), number or packing density of cells (von Economo and Koskinas, 1925; Haug et al., 1984; Zilles et al., 1986; Meyer et al., 2010), density of myelinated fibers (Vogt and Vogt, 1919; Annese et al., 2004), and densities of various transmitter receptors (e.g., Cortés et al., 1986, 1987; Hoyer et al., 1986a,b; Pazos et al., 1987a,b; Jansen et al., 1989; Scheperjans et al., 2005a; Eickhoff et al., 2008; Amunts et al., 2010; Vogt et al., 2013; Zilles and Palomero-Gallagher, 2017). For recent reviews see Nieuwenhuys (2013) and Zilles et al. (2015b).

Cortical layers also differ by their input and output, as well as by the preferred direction of connections with other cortical areas (Rockland and Pandya, 1979; Felleman and Van Essen, 1991; Rockland, 1997, 2015; Markov and Kennedy, 2013; Markov et al., 2014). The feedforward connection from V1 to V2 has cells of origin mainly in layers III and IVb (Kennedy and Bullier, 1985; Sincich et al., 2010). Cells which give rise to feedback connections are typically distributed over several cortical layers and are found in the supragranular layers II to upper layer III, and the infragranular layer VI. However, the specific differentiation into layers in V1, and the organization of functionally diverse visual input (direction, color, shape) makes V1 to an example which cannot be generalized for the entire cortex. Although most of the source neurons of feedforward pathways are present in the supragranular layers and terminate in the same layers of the target region, the source neurons of feedback pathways are found in the infragranular layers, but terminate in both supra- and infragranular layers (Rockland and Van Hoesen, 1994; Markov et al., 2014). Thus, a single cortical layer does not exclusively contain feedforward or feedback neurons; instead they are found in varying proportions in both of these strata (Barone et al., 2000).

Also important for the present analysis of receptor fingerprints is the location of the terminal fields of connections which build most of the synapses, and thus must contain most of the receptors required for signal processing. Cortico-cortical neurons of the supragranular layers extend their apical dendrites up to layer I, where they form tufts, whereas not all of the infragranular neurons reach layer I with their apical dendrites, but have most of their dendritic arborizations in supragranular layers (Lund et al., 1981; Katz, 1987; Hübener et al., 1990; Mohan et al., 2015). Layer IV neurons receive most of their input from thalamo-cortical connections. Since transmitter receptors are key molecules of signal transmission, we hypothesized that the distinct regional and laminar distribution patterns of multiple transmitter receptors may also contribute to a better understanding of connectivity. It is hitherto largely unknown, whether the regional and laminar density of transmitter receptors and the locally distinct balances between the densities of multiple receptor types (regional and laminar receptor fingerprints) reflect hierarchies of cortical areas, and also may provide insight into principle rules of cortical architecture and connectivity. It is also unknown whether the receptor fingerprints of the three major cortical strata studied here are similar, or each of them exhibits distinct patterns. Therefore, densities of 15 different receptor types are studied in the supragranular, granular and infragranular layers of 44 human visual, somatosensory, auditory and multimodal association areas. The relatively large number of receptors and cortical areas enables the detection of probably general rules valid for the entire cerebral cortex.

## Materials and Methods

Brains of three donors without any record of neurological or psychiatric diseases or of long-term drug treatment (age range: 72–77 years; 2 males, 1 female) were removed at autopsy. Subjects had given written consent before death and/or had been included in the body donor program of the Department of Anatomy, University of Düsseldorf, Germany, which also requires written consent by the donor. All procedures complied with the requirements defined by the local ethical committee. Post mortem delay before deep freezing was between 8 h and 18 h. Causes of death were cardiac arrest, lung edema, and myocardial infarction. After having separated each hemisphere into approximately 3 cm thick slabs, the slabs were shock frozen in isopentane at −40°C and stored at −80°C in airtight plastic bags until further processing. Thus, brain tissue was not treated with any chemical fixation substances. The total post mortem delay, including the deep freezing step, varied between 8 h and 18 h.

The unfixed frozen slabs were serially sectioned in the coronal plane (section thickness 20 μm) with a large scale cryostat microtome. Alternating sections were processed for quantitative *in vitro* receptor autoradiography, or stained for the visualization of cell bodies (Merker, 1983) or of myelin (Gallyas, 1979) using silver staining methods. Fifteen different receptors for glutamate (AMPA, NMDA, kainate), GABA (GABA_A_, GABA_A_ benzodiazepine binding sites [GABA_A_/BZ], GABA_B_), acetylcholine (muscarinic M_1_, M_2_, M_3_, nicotinic α_4_β_2_), noradrenaline (α_1_, α_2_), serotonin (5-HT_1A_, 5-HT_2_), and dopamine (D_1_) were identified using tritium-labeled ligands according to previously published receptor protocols (Zilles et al., 2002a,b; Graebenitz et al., 2011; Palomero-Gallagher and Zilles, 2017b) which are summarized in Supplementary Table S1. In short, sections were rehydrated during the pre-incubation, and endogenous substances which could block the binding site for the tritiated receptor ligands were removed. Sections were then incubated in buffer solutions containing the receptor-specific tritiated ligand (in nM concentrations), or the tritiated ligand plus a non-labeled specific displacer (in mM concentrations). Incubation with the labeled ligand alone demonstrates total binding, whereas incubation with the tritiated ligand and the displacer reveals the non-specific binding. Specific binding can be calculated as the difference between total and non-specific binding. Since for the experimental protocols used here non-specific binding only amounted to 95% of total binding in all cases, we consider autoradiographs visualizing total binding to also be representative for the specific binding of the ligand in question.

The labeled sections were exposed against tritium-sensitive films (Hyperfilm, Amersham, Braunschweig, Germany) together with plastic (Microscales^®^, Amersham) or brain tissue scales (with a known protein density) containing step-wise increasing radioactivity concentrations. Protein content in the homogenate used to create the brain tissue scales had previously been determined by means of the Lowry method (Lowry et al., 1951), and radioactivity concentrations had been measured by liquid scintillation. The resulting autoradiographs were digitized by means of an image acquisition and processing system Axiovision (Zeiss, Germany) for subsequent densitometric analysis (Zilles et al., 2002b; Palomero-Gallagher and Zilles, 2017b). The relationship between the gray value of a pixel in the digitized autoradiograph and the receptor binding site density was defined in two steps: first, the gray value images of the co-exposed scales were used to compute a calibration curve by non-linear, least-squares fitting, thus defining the relationship between gray values in the autoradiographs and concentrations of radioactivity. Then, these concentrations of radioactivity were corrected to account for experimental conditions (e.g., specific activity, dissociation constant and free concentration of the ligand during incubation) by means of formula 1:

(1) $$C_{b}=\frac{R}{E\cdot B\cdot W_{b}\cdot S_{a}}\cdot \frac{K_{D}+L}{L}$$

where *R* is the concentration of radioactivity in counts per minute (cpm), *E* is the efficiency of the scintillation counter (depends on the actual counter), *B* is a constant representing the number of decays per unit of time and radioactivity (Ci/min), *W_b_* the protein weight of a standard (mg), *S_a_* the specific activity of the ligand (Ci/mmol), *K_D_* the dissociation constant of the ligand (nM), and *L* the free concentration of the ligand during incubation (nM). Thus, the gray value of each pixel in a digitized autoradiograph, which can be color coded for visualization purposes, codes for a receptor concentration per unit protein (B_max_, in fmol/mg protein) at saturation of ligand-receptor complexes.

Equidistant receptor profiles oriented vertically to the cortical surface were extracted by means of a minimum length algorithm from the linearized autoradiographs (Schleicher et al., 2000). Special attention was given to collect the profiles at sites where the cortex is not obliquely or tangentially sectioned. These profiles quantify the receptor density from the pial surface to the border between layer VI and the white matter, and were obtained from cytoarchitectonically defined regions. These receptor profiles were subdivided into three strata representing the supragranular (layers I–III), granular (layer IV) and infragranular (layers V–VI) layers by overlaying the laminar borders visible in the neighboring cell body-stained sections (cytoarchitectonic definition of the laminar borders). Since the motor cortex (areas 4 and 6) does not have a clearly recognizable granular layer (layer IV, Brodmann, 1909; von Economo and Koskinas, 1925), but the typical cells of layer IV have been demonstrated at the border region between layers III and V (García-Cabezas and Barbas, 2014; Barbas and García-Cabezas, 2015), we tentatively defined layer IV of areas 4 and 6 as stripe with a thickness of 3% of the total cortical depth below the lower border of layer III. A 3% thickness for layer IV is an estimate derived from the thickness of this layer in the rostrally adjoining prefrontal cortex (von Economo and Koskinas, 1925). The surface defined beneath a receptor profile, or beneath the discrete sectors defined by the position of borders between strata, can be computed to yield the absolute binding site densities for the entire cortical depth (mean density over all layers) or for the each of the three strata in each particular area. Differential shrinkage between autoradiographs and neighboring silver-stained sections does not play a role, since we projected the cell-body (silver) stained section onto the images of the different receptor autoradiographs by means of a microscope equipped with a drawing tube. Thus, we could control the precise spatial matching of the autoradiographs and the histologically stained sections.

**Figure 1:**
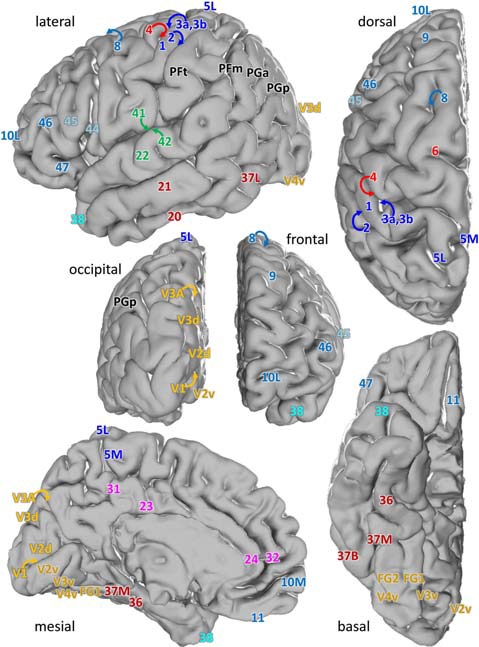
Location of the analyzed cortical regions in the single subject template brain of the Montreal Neurological Institute. Location of areas: 10M, 10L from Bludau et al. (2014); 24, 32 from Palomero-Gallagher et al. (2009), 44 and 45 from Amunts et al. (1999), 1, 2, 3a, 3b, 4, 5L and 5M from Geyer et al. (1996, 1999), Grefkes et al. (2001) and Scheperjans et al. (2008), PFm, PFt, PGa, PGp from Caspers et al. (2006), 41, 42 from Morosan et al. (2001, 2005), V1, V2 from Amunts et al. (2000), V3A, V3d, V3v, V4v from Rottschy et al. (2007) and Kujovic et al. (2013), FG1, FG2 from Caspers J. et al. (2013) and of all other areas from Brodmann (1909).

We examined laminar distributions of 15 different receptors in 44 iso- and periallocortical areas (Figure 1). Regions were defined based on the description by Brodmann (1909), or the JuBrain Atlas (Amunts and Zilles, 2015). In detail, the cortical areas were described in the following publications:

- Isocortical prefrontal areas 11, 8, 9, 10L, 10M, 46 and 47 (Brodmann, 1909),
- 4 and 6 (primary motor and premotor cortices; Brodmann, 1909),
- 3b, 1, 2, and 3a (primary somatosensory cortex; Brodmann, 1909; Jones, 1986; Geyer et al., 1999; Grefkes et al., 2001),
- V1 (primary visual cortex, cytoarchitectonical area 17; Brodmann, 1909; Amunts et al., 2000),
- Dorsal (V2d) and ventral (V2v) parts of the secondary visual cortex (cytoarchitectonical area 18; Brodmann, 1909; Amunts et al., 2000),
- V3d, V3A, V3v, V4v, FG1 and FG2 (higher visual areas, cyto- and receptorarchitectonically defined areas hOc3d, hOc4d, hOc3v, hOc4v, FG1 and FG2, respectively; Rottschy et al., 2007; Caspers J. et al., 2013; Kujovic et al., 2013; Caspers et al., 2015),
- 44 and 45 (receptorarchitectonically defined ventral and anterior portions of Brodmann’s areas 44 (44v) and 45 (45a) in Broca’s region; Amunts et al., 2010),
- superior parietal areas 5L and 5M (cyto- and receptorarchitectonically identified areas of the higher unimodal somatosensory cortex; Scheperjans et al., 2005b, 2008),
- inferior parietal areas PFt, PFm, PGa, and PGp (cyto- and receptorarchitectonically defined; Caspers et al., 2006; Caspers S. et al., 2013),
- primary and higher unimodal auditory areas 41, 42, and 22 (cytoarchitectonically defined Te1, Te2 and 22; Morosan et al., 2001, 2005),
- lateral, medial and basal portions of the parieto-temporo-occipital regtion (37L, 37M and 37B, respective parts of area 37; Brodmann, 1909),
- multimodal temporal areas 20, 21, 36 and 38 (Brodmann, 1909),
- periarchicortical cingulate area 24 and isocortical cingulate areas 23, 31 and 32 (Brodmann, 1909; Palomero-Gallagher et al., 2008).

Analysis of the region-specific balance between the densities of multiple receptors in a single cortical area prompted the introduction of the term receptor fingerprint (Zilles et al., 2002a). The size of a fingerprint is given by the area of the polar coordinate graph and the actual shape of a fingerprint depends on the contribution by the absolute density of each receptor type to the multi-receptor fingerprint. The shape reflects the balance between the different receptor types in each area. For comparison of fingerprints between different cortical areas, the sequence of receptors around the polar graph and the scaling of absolute receptor densities must be identical in each cortical area. Multivariate analyses of the multi-receptor fingerprints were conducted to visualize putative clusters of areas and strata according to the degree of (dis)similarity of their fingerprints using the Matlab Statistics Toolbox (MatLab R2009a; Mathworks Inc., Natick, MA, USA), in house R-scripts and Systat (Systat 13; Systat Software Inc., Chicago, IL, USA). In these analyses, receptor fingerprints were treated as feature vectors describing the balance between all receptors studied here in a defined cortical area or its strata. Before each analysis, densities were normalized by computing *z*-scores for each receptor type separately, thus ensuring an equal weighting of each receptor without eliminating relative differences in receptor densities among areas or each of their three strata. Hierarchical cluster analyses were performed as previously described (Palomero-Gallagher et al., 2009) using the Euclidean distance as a measure of (dis)similarity and the Ward linkage algorithm as the linkage method. Euclidean distances were chosen because they take both the differences in size and in shape of receptor fingerprints into account, and in combination with the Ward linkage yielded the maximum cophenetic correlation coefficient as compared to any combination of alternative dissimilarity measurement and linkage methods. The number of clusters in the dendrograms was defined using k-means clustering. A multidimensional scaling analysis was carried out as described previously (Sherwood et al., 2004) using the Kruskal stress scaling method to reduce the 15-dimensional space resulting from the analysis of 15 different receptors into two dimensions for graphical representation of the Euclidean distances between the stratum-specific fingerprints of cortical areas.

To determine whether the density (over all layers) of each receptor type separately was homogeneously or not homogeneously distributed over the 44 areas, ANOVA tests were carried out and *p* values were Bonferroni corrected for multiple comparisons (15 receptor types). Threshold was set at *p* ≤ 0.05. Subsequently, one-sample *t*-tests were carried out for each receptor type to determine whether its density in a given area differed significantly from the mean density of that receptor over all examined areas (expected value). Since these tests were only carried out for the receptor types for which the ANOVA was found to be significant, *p* values (threshold *p* ≤ 0.05) of the one-sample *t*-tests must not corrected for multiple testing. The same procedure was applied to densities measured in each of the three strata.

For the question whether the 44 areas significantly differed in their receptor fingerprints, a discriminant analysis was carried with “area” as a grouping factor. This enables the determination of homogeneity or inhomogeneity of the fingerprints between areas. Since the discriminant analysis over all areas indicated a highly significant inhomogeneity of the fingerprints between the areas (*p* < 0.000), a pairwise comparison between all areas was also performed. The *p* values of these subsequent discriminant analyses were not corrected for multiple comparisons, because the Omnibus test was significant and the subsequent tests were performed as *post hoc* tests. This procedure was carried out for the fingerprints of the mean densities over all layers as well as for those of each of the three strata.

## Results

### Transmitter Receptors Are Heterogeneously Distributed Over Regions and Layers in the Human Cerebral Cortex

**Figure 2:**
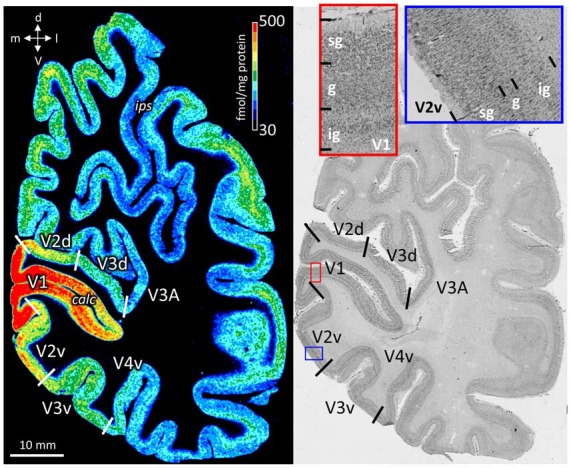
Neighboring coronal sections through the human occipital lobe. Left: distribution of the cholinergic muscarinic M_2_ receptors. Color bar codes for receptor densities in fmol/mg protein. Right: Cell body stained section. Red contoured inset from primary visual cortex (V1) and blue contoured inset from V2v. The high magnifications in cell body stained sections were used to define the borders of the supragranular (sg), granular (g), and infragranular (ig) strata, which were then manually traced in the neighboring receptor autoradiograph. calc, calcarine sulcus; d, dorsal direction; ips, intraparietal sulcus; l, lateral direction; m, medial direction; v, ventral direction.

The color coded images of receptor densities give a first impression of their heterogeneous regional and laminar receptor distribution (Figure 2). By comparison with neighboring cell body stained sections, the precise upper and lower limits of the cerebral cortex and the borders between layers were determined (Figure 2), and then used to define the borders of supragranular, granular and infragranular strata in the receptor profiles.

The regional densities (fmol/mg protein) of 15 different transmitter receptors for glutamate, GABA, acetylcholine, dopamine, noradrenaline and serotonin were measured in the three strata (supragranular, granular and infragranular strata) of 44 cytoarchitectonically defined iso- and periarchicortical areas of the human brain. Additionally, the mean density of each receptor over all cortical layers (mean areal density) was calculated. All original data are provided in Supplementary Table S2.

#### Mean Areal Densities of Single Transmitter Receptors in the Human Cerebral Cortex

**Figure 3:**
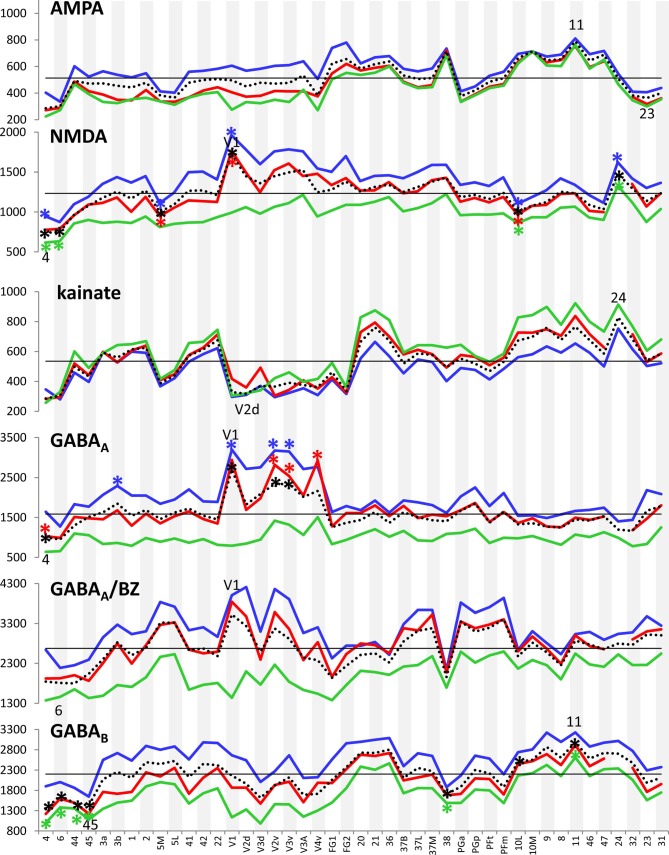

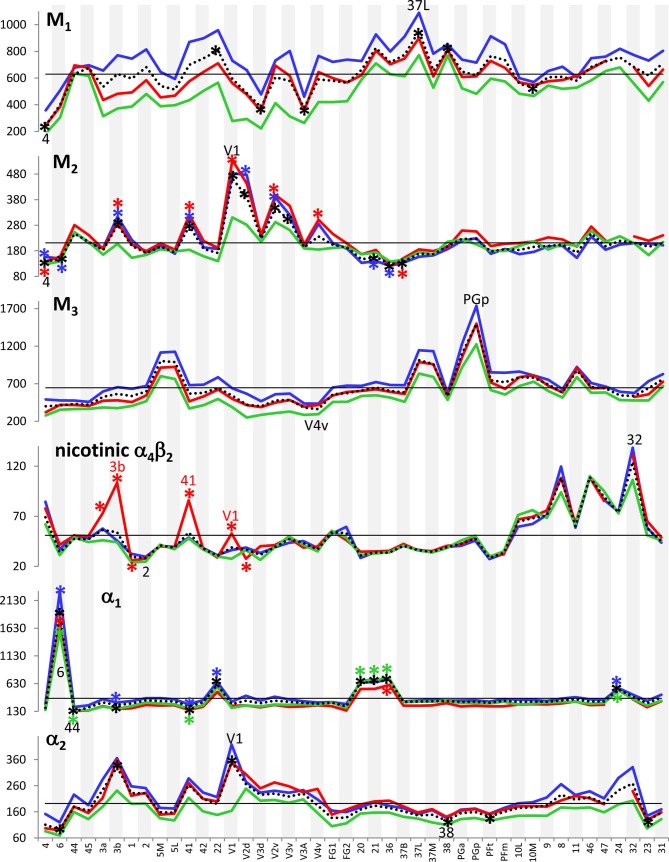

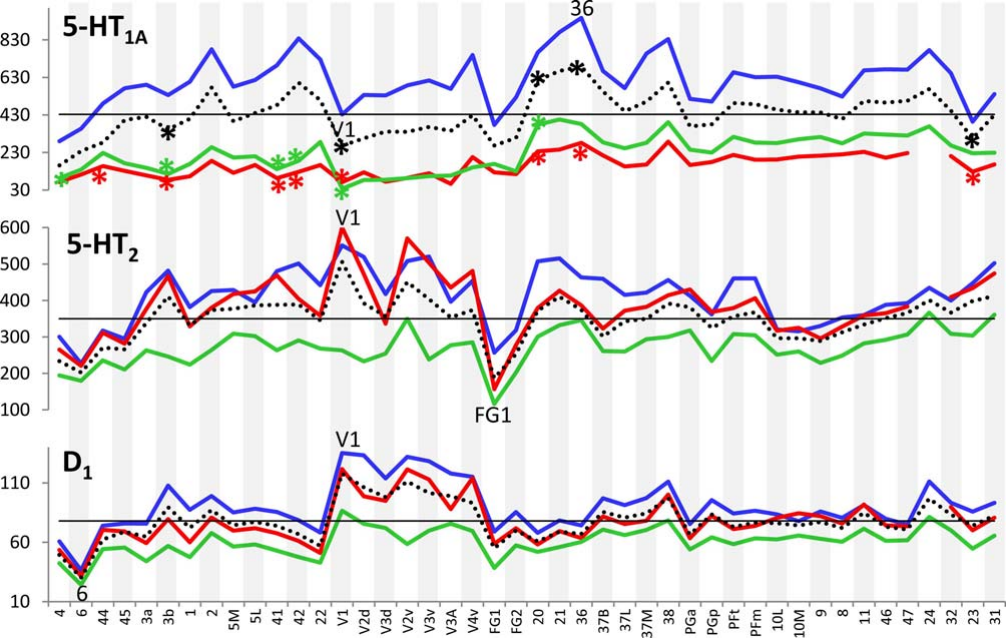
Absolute densities (fmol/mg protein) of each of the studied receptor types in 44 cortical areas. Dotted line: mean areal receptor density; blue line: receptor density in the supragranular stratum; red line: receptor density in the granular stratum; green line: receptor density in the infragranular stratum; straight black line indicates the mean areal density of each receptor averaged over all 44 areas. Areas with maximal or minimal mean areal receptor densities are indicated by their names in the respective graphs. In the case of the nicotinic α_4_β_2_ receptor, the absolute density of this receptor in the granular stratum of the three primary sensory areas (V1, 3b and 41) is highlighted. Asterisks indicate maxima and/or minima in the densities of supragranular (in blue), granular (in red), infragranular (in green) or all layers (in black) which differ significantly from the mean density of the given receptor averaged over all examined areas.

Figure 3 provides an overview of the strata-specific and mean areal receptor densities of all 44 analyzed areas. ANOVAs revealed significant differences in mean densities (averaged over all layers) as well as in those of the three strata only for the NMDA, GABA_A_, GABA_B_, M_1_, M_2_, α_1_, α_2_ and 5-HT_1A_ receptors (*p* values after Bonferroni correction, see Table 1). Interestingly, also the nicotinic α_4_β_2_ receptors reached significance when densities of the granular stratum were analyzed. AMPA, kainate, M_3_, 5-HT_2_ and D_1_ receptors were not significant in the ANOVA. Therefore, significance of minima and maxima was not tested in these cases. The *mean areal densities* (dotted line in Figure 3) demonstrate that NMDA, GABA_A_, GABA_A_/BZ, M_2_, α_2_, 5-HT_2_, and D_1_ receptors reach their maximal densities in V1. The absolute maxima of other receptors are found in areas 24 (NMDA), 11 (AMPA, GABA_B_), lateral part of area 37 (M_1_), PGp (M_3_), 32 (nicotinic α_4_β_2_), 6 (α_1_), and 36 (5-HT_1A_). The lowest densities of AMPA receptors are reached in the posterior cingulate area 23, NMDA receptors in area 4, kainate receptors in area V2d, GABAergic GABA_A_ receptors and GABA_A_/BZ binding sites in the motor cortex (areas 4 and 6, respectively), and of GABA_B_ receptors in area 45. Muscarinic M_1_ and M_2_ receptors have their minima in area 4, M_3_ receptors in area V4v, nicotinic α_4_β_2_ receptors in area 2, adrenergic α_1_ receptors in area 44, adrenergic α_2_ receptors in area 38, serotonergic 5-HT_1A_ receptors in area V1, serotonergic 5-HT_2_ receptors in area FG1, and dopaminergic D_1_ receptors in premotor area 6. Despite of this considerable regional heterogeneity of the maximal and minimal mean areal densities of single receptor types, regional preferences can be detected. NMDA, GABA_A_, GABA_A_/BZ, M_2_, α_2_, 5-HT_2_, and D_1_ receptors all reach high densities in the primary visual cortex V1, whereas the same receptor types (except α_2_ and 5-HT_2_) and the M_1_ receptor show very low densities over all layers in primary motor and premotor areas.

**Table 1:** *p* values (after Bonferroni correction) of the ANOVA tests carried out to determine the inhomogeneous distribution (*p* ≤ 0.05) of 15 receptor types throughout the 44 areas in the three brains (over all layers, or in the supragranular, granular or infragranular strata).

Receptor	All layers	Supragranular	Granular	Infragranular
AMPA	7.190	7.468	1.2936	0.981
NMDA	**0.000**	**0.000**	**0.000**	**0.001**
Kainate	11.386	13.877	13.651	12.354
GABA_A_	**0.000**	**0.003**	**0.007**	0.787
GABA_A_/BZ	3.917	6.892	6.093	7.391
GABA_B_	**0.014**	0.176	0.104	**0.013**
M_1_	**0.043**	0.477	0.092	0.171
M_2_	**0.000**	**0.000**	**0.000**	0.106
M_3_	5.617	0.472	0.274	0.088
α_4_β_2_	0.246	0.258	**0.018**	5.837
α_1_	**0.000**	**0.000**	0.687	**0.000**
α_2_	**0.050**	0.532	1.273	8.385
5-HT_1A_	**0.018**	9.792	**0.000**	**0.000**
5-HT_2_	12.009	4.544	8.523	12.998
D_1_	1.231	2.040	0.188	5.412

*Significant values highlighted in bold font*.

#### Strata Specific Densities of Transmitter Receptors in the Human Cerebral Cortex

If we focus on the *strata-specific densities* of each receptor type (Supplementary Table S2), the courses of all three strata seem to run nearly parallel to each other throughout all cortical areas (Figure 3). Thus, regionally coincident maxima of all three strata are found for GABA_B_, M_2_, and D_1_ receptors in area V1, and for M_1_ and M_3_ receptors in areas 37L and PGp, respectively. Coincident maxima in the supragranular and granular strata occur in area V1 (NMDA, GABA_A_, 5-HT_2_), and in the anterior cingulate area 32 (nicotinic α_4_β_2_). The supra- and infragranular strata reach coincident maxima in the orbitofrontal area 11 (AMPA) and the premotor area 6 (α_1_, D_1_), whereas such coincident maxima of granular and infragranular layers are found in areas 11 (kainate) and 3b (α_2_) (Figure 3). Coincident minima of 5-HT_2_ receptors are found in area FG1 in all three strata, whereas NMDA and kainate receptors show such minima in supragranular and granular layers of area V1, GABA_A_/BZ binding sites in area 6, GABA_B_ in area 45, and α_1_ in area 44. Coincident minima in supra- and infragranular strata are found in area 6 for the D_1_ receptor, and in granular and infragranular strata for NMDA, GABA_A_, GABA_A_/BZ and M_2_ receptors in the primary motor or the premotor cortices 4 and 6, respectively (Figure 3).

Large differences between 5-HT_1A_ receptor densities of the different strata are visible throughout all regions studied (Figure 3). The density of the 5-HT_1A_ receptor is considerably higher in the supragranular stratum than in the other two strata, suggesting a modulatory influence preferably on cortico-cortical projection neurons and interneurons, which are more frequent in this stratum than in the other two strata, as well as on the apical dendrites of the pyramidal cells located in deeper layers. The location of exceptionally high densities of the nicotinic α_4_β_2_ receptor only in the granular layer of all three primary sensory areas is also notable (Figure 3), since these three maxima considerably differ from the density of this receptor in the supra- and infragranular strata of the same areas (3b, 41 and V1). Thus, the predominant input layer of primary sensory cortices seems to be under a strong modulatory influence of this cholinergic receptor type. Three coincident maxima of α_4_β_2_ receptors are visible in all strata of the multimodal association areas 8 and 46 of the prefrontal cortex and the anterior cingulate cortex (area 32). In all other areas the density of this receptor is low, and shows no clear-cut local preference.

In most areas a canonical sequence of receptor densities from highest values in the supragranular stratum, intermediate values in the granular layer IV, and lowest values in the infragranular stratum is found. Exceptions from this rule are seen for the kainate receptor, which shows highest densities in the infragranular stratum, the M_2_ receptor, which reaches highest densities in the granular stratum of most areas, and the α_1_ and 5-HT_1A_ receptors, which in some areas present higher densities in the infragranular than in the granular stratum.

### Multi-Receptor Fingerprints of Areas and Layers Reflect Principle Aspects of Cortical Organization

**Figure 4:**
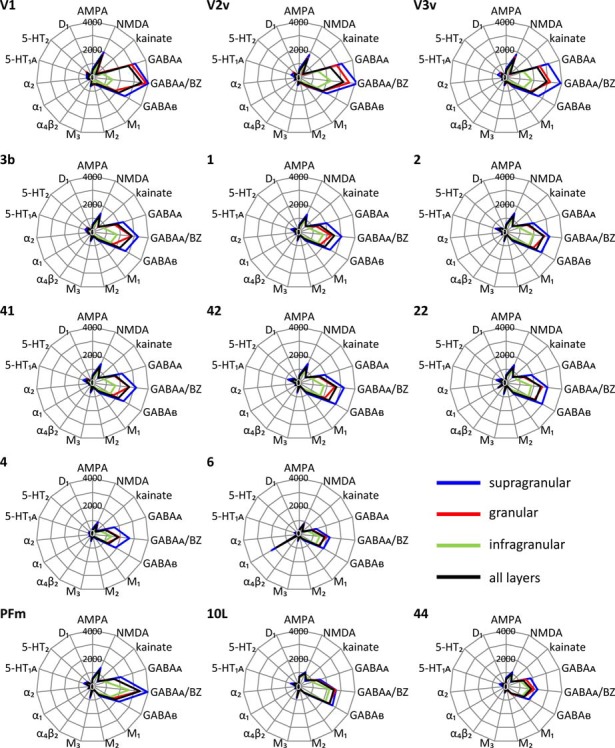
Absolute multi-receptor fingerprints of 15 different receptor types in each of the 44 cortical areas. From these areas, V1, V2v and V3v (visual system), 3b, 1 and 2 (somatosensory system), 41, 42 and 22 (auditory system), 4 and 6 (motor cortex), PFm (inferior parietal cortex), 10L (lateral part of the frontopolar cortex) and 44 (part of Broca’s region) were chosen as typical fingerprints representing different functional systems. The fingerprints of all other areas are found in Supplementary Figure S1. Scaling of the absolute fingerprints in fmol/mg protein is the same in all areas.

**Figure 5:**
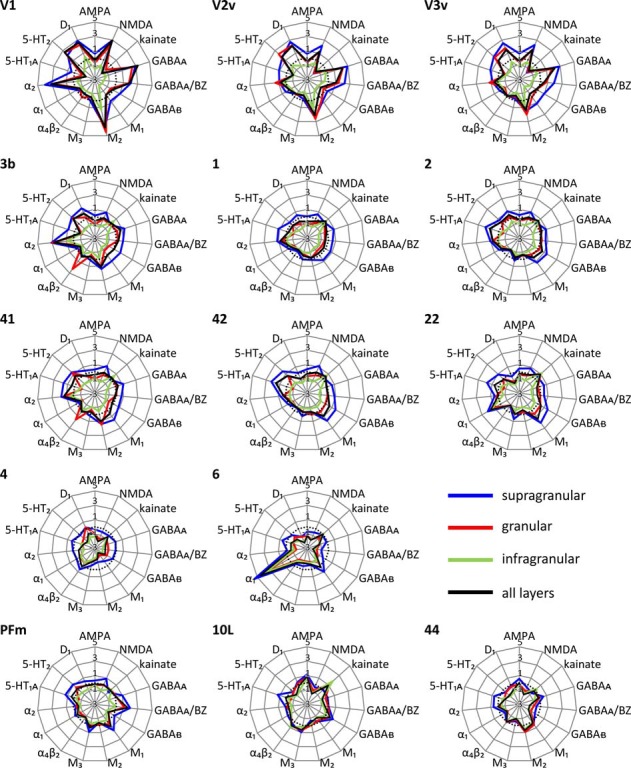
Normalized multi-receptor fingerprints of 15 different receptor types in each of the 44 cortical areas. For further information see Figure 4. The fingerprints are normalized by their *z*-scores, and the dotted line indicates the average *z*-score over all areas. Positive *z-scores* indicate receptor densities above average, negative *z-scores* those below average. The normalized fingerprints of all other areas are found in Supplementary Figure S2.

Each cortical area expressed all receptor types, but at different mean areal and laminar densities (Supplementary Table S2). The regional-specific expression of all receptors studied constitutes the receptor fingerprint of an area or a stratum. We generated four receptor fingerprints per cortical area, visualizing the mean areal density, as well as the density in its supragranular, granular and infragranular strata. This is done for both the absolute (for selected areas see Figure 4, for all other areas see Supplementary Figure S1), and the *z*-score normalized (for selected areas see Figure 5, for all areas see Supplementary Figure S2) receptor densities, which is done because the absolute densities of the various receptors differ by the order of one to two magnitudes. Specifically, the GABA and glutamate receptors reach much higher densities than those of all other receptor types in the absolute fingerprints (Figure 4, Supplementary Figure S1). Therefore, it is difficult to estimate the contribution of the modulatory receptors to the shape of the fingerprint because there receptors occur at much lower densities. In the normalized fingerprints, a receptor density above the mean of that receptor over all examined areas has a positive z-score, a receptor density below the mean of that receptor has a negative *z-score* (Figure 5, Supplementary Figure S2). The normalized fingerprint facilitates a visual comparison of the relative contribution of a single receptor to the fingerprint of each area.

Discriminant analyses of the fingerprints over all layers, and separately for the three strata shows that the fingerprints of all 44 areas are heterogeneous (*p* = 0.000 for each of the analyses). After these Omnibus tests, a pairwise comparison between all combinations of areas revealed some significant inter-areal differences, but most comparisons did not reach significance (Supplementary Tables S3–S6).

#### Absolute Fingerprints of Cortical Areas

Since a pure visual comparison between the different fingerprints depends on the interpretation by the observer, we quantified the size of the mean areal and strata-specific fingerprints by computing the sum of the densities of all receptors over all layers or in each of the three strata.

**Figure 6:**
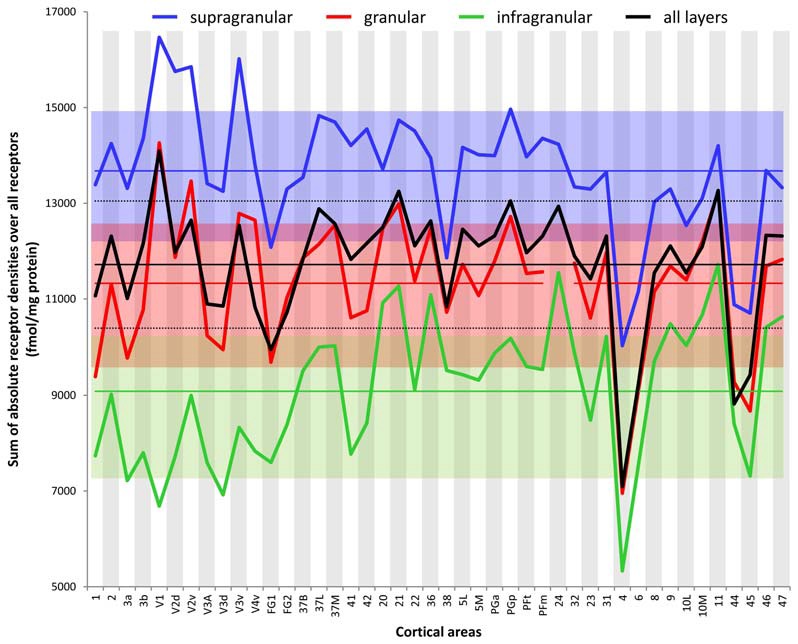
Sum of the absolute densities of all receptors examined in each area and stratum as well as over all layers of each brain region. Mean values over all areas of the three strata and the total cortical depth and their standard deviations are indicated by straight lines and dotted lines, respectively.

The ranges of the sizes overlap slightly between the strata, if the standard deviations are taken as measure (Figure 6). The comparison between all areas reveals a general rule: the areal sizes of absolute fingerprints are always larger in the supragranular stratum, followed by the granular and then the infragranular stratum (Figure 6). The size of the fingerprint over all layers shows values above the grand mean plus standard deviation in the primary visual cortex V1, ventral part of V2, temporo-occipital transition area 37L, temporal association area 21, parietal association areas 5L and PGp, as well as orbitofrontal area 11. Corresponding lowest values are found in the higher visual area FG1, the temporal association area 38, the motor cortical areas 4 and 6, as well as in the Broca areas 44 and 45. Significant higher values for the supragranular fingerprints were found only in the early visual areas V1, dorsal and ventral parts of V2 as well as in V3v. The corresponding lowest values were found in the same areas as described for the size of the mean areal fingerprints (FG1, 38, 4, 6, 44, 45). Significant higher values for the granular fingerprints were found in V1, V2v, V3v, 21 and 11. The corresponding lowest values were found in the somatosensory area 1, higher visual area FG1, motor areas 4 and 6, as well as in Broca areas 44 and 45. The highest values of the infragranular fingerprints are found in temporal association areas 20, 21, 36, anterior cingulate area 24, posterior cingulate area 31, as well as in the prefrontal areas 9, 10L, 10M, 11, 46 and 47. The corresponding lowest values were seen in the somatosensory area 3a, visual areas V1, V3A, V3d, and FG1, motor areas 4 and 6, as well as in Broca’s area 45.

Although the absolute densities of the different receptors vary between the examined brains (as revealed by the SD values and the variation coefficients specified in Supplementary Table S2), the proportional changes in densities between cortical areas remain constant when comparing different brains; e.g., in all examined brains, V1 contained higher overall NMDA, GABA_A_, or α_2_, but lower α_1_, or 5-HT_1A_ receptor densities than did V2. Likewise, the relationship between receptor densities in the examined strata was also constant in the different brains examined; e.g., in area V1 of all brains highest 5-HT_1A_ receptor densities were always found in the supragranular stratum, and lowest ones in the infragranular stratum.

In conclusion, the sizes of absolute fingerprints, and thus the density of all receptors together in each area or stratum, are regional-specific and show a canonical sequence (supragranular to granular to infragranular) of the strata from large to small fingerprints.

#### Normalized Fingerprints of Cortical Areas

Using the normalized fingerprints, differences in the *shape*, and thus in the regional balance between multiple receptors can be better visualized.

##### Unimodal sensory areas

The shapes of the normalized fingerprints (Figure 5, Supplementary Figure S2) of visual areas clearly differ from those of the somatosensory and auditory systems with a notably higher similarity between the fingerprints of the latter two functional systems. The impact of receptor density analyses on revealing regional organizational principles of the cortex is further supported by the exceptionally high nicotinic α_4_β_2_ receptor densities in the granular layers of the core regions of the primary somatosensory (3b) and auditory (41) cortices. Within each of the three sensory systems, the fingerprints are most similar between primary and early sensory areas (areas V1, V2d, and V2v in the visual system; areas 1, 2, 3a and 3b in the somatosensory system; areas 41 and 42 in the auditory system). Furthermore, the fingerprints of early unimodal visual areas (V3v, V3A, V3d, V4v) are more similar to V1and V2 than to the hierarchically higher visual areas (areas FG1 and FG2 of the fusiform gyrus). Notably, the fingerprints of the early visual areas of the dorsal stream (V3A, V3d) differ from those of the ventral stream (V3v, V4v). The primary visual area V1 shows considerably higher normalized densities of NMDA, GABA_A_, GABA_A_/BZ, M_2_, α_2_, 5-HT_2_ and D_1_ receptors in all strata than the primary somatosensory (1, 2, 3a and 3b) and auditory (41) areas (Figure 5, Supplementary Figure S2).

The fingerprints of areas of the primary auditory (area 41) and the secondary and multimodal auditory/temporal areas (areas 42, 20–22, 36) systematically differ. Particularly, the normalized density of the α_1_ receptor is higher in the association areas 20–22 and 36 compared to the unimodal auditory areas 41 and 42. The fingerprint of the temporo-polar area 38 differs in shape from all other temporal areas studied here. Likewise, the fingerprints of the temporo-occipital transition region (areas 37B, 37L and 37M) differ from those of the areas of the temporal and occipital lobes (Figure 5, Supplementary Figure S2).

The normalized fingerprints of motor areas 4 and 6 completely contrast with those of all sensory areas. Additionally, the high density of α_1_ receptors in the premotor cortex (area 6) contributes to the segregation of this area from the primary motor cortex (area 4).

##### Multimodal association areas

Multimodal association regions are located in the prefrontal, temporal and parietal lobes including the precuneus region. Areas of the inferior parietal lobule are clearly segregated by different shapes of receptor fingerprints into two different groups: the supramarginal group with areas PFm and PFt, and the angular group with areas PGa and PGp. Both groups of fingerprints are separated by the high to very high density of the muscarinic M_3_ receptors in the angular group, and a lower density of this receptor in the supramarginal group which resembles only that of the average over all 44 areas. Notably, the fingerprints of PGa and the postcentral areas 5L and 5M are very similar, although the latter areas are not located in the inferior parietal lobule. Additional to their high M_3_ receptor density, the supragranular and granular strata of 5L, 5M, PGa and PGp show a GABA_A_/BZ density clearly above average. Thus, both receptors largely contribute to the similarity of the fingerprints between these four parietal areas and segregate them from PFm and PFt.

In a next step the area- and stratum-specific fingerprints were tested to answer two questions:

- Do the fingerprints of the three strata build separate, strata-specific clusters if all cortical areas are compared?
- Do the fingerprints over all layers and/or the stratum-specific fingerprints systematically differ by their shapes and sizes between cortical areas? Do these variations indicate principal aspects of functional and topographical segregation, as well as hierarchical organization?

#### Multidimensional Scaling Analysis of Receptor Fingerprints

**Figure 7:**
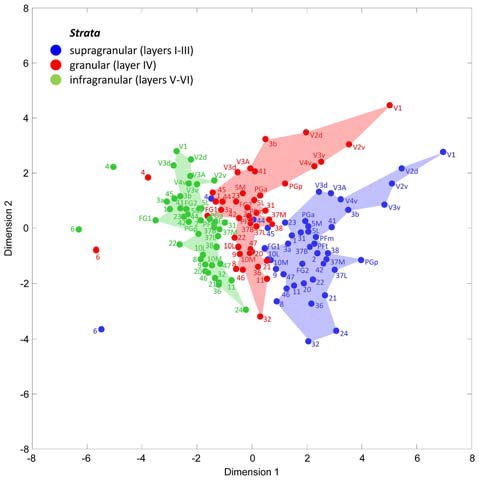
Multidimensional scaling analysis to visualize the different clusters of receptor fingerprints extracted from the supragranular, granular and infragranular strata of all examined areas. Note the exceptional positions of both motor areas 4 and 6, and of the Broca areas 44 and 45. These are the only areas whose supragranular and granular fingerprints take positions outside the cluster of all other areas.

A multidimensional scaling analysis of the stratum-specific fingerprints shows three clusters (Figure 7), which clearly separate the fingerprints of the supragranular from those of the granular and the infragranular strata in nearly all areas. Only exceptions are the positions of fingerprints of the granular stratum of the motor areas 4 and 6, which are shifted into the range of the infragranular cluster. Furthermore, the fingerprints of the supragranular stratum of areas 4, 44 and 45 are slightly shifted into the cluster of the fingerprints of the granular stratum. The cause of these exceptional shifts will be addressed in the “Discussion” Section. In conclusion, the laminar fingerprints of the iso- and periarchicortex completely differ between the three strata. Therefore, the multi-receptor densities are specific for each of the three groups of cortical layers (strata), and thus indicate a canonical receptor balance in each stratum.

#### Hierarchical Cluster Analyses of Receptor Fingerprints

**Figure 8:**
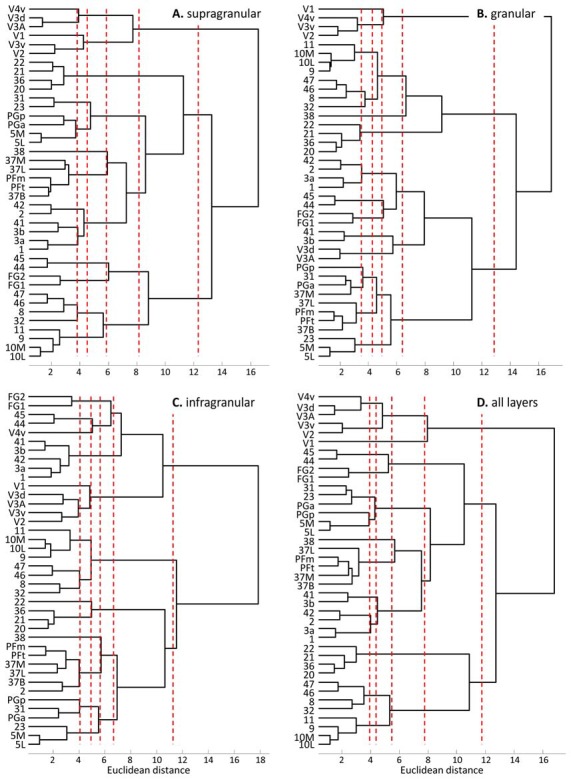
Hierarchical clustering of all regions except the agranular cortices of areas 4, 6 and 24 based on the receptor fingerprints extracted from their supragranular **(A)**, granular **(B)**, or infragranular **(C)** strata, or based on the fingerprints of mean receptor densities over all layers **(D)**. Dashed red lines indicate the number of “main” clusters as determined by the k-means analyses.

**Figure 9:**
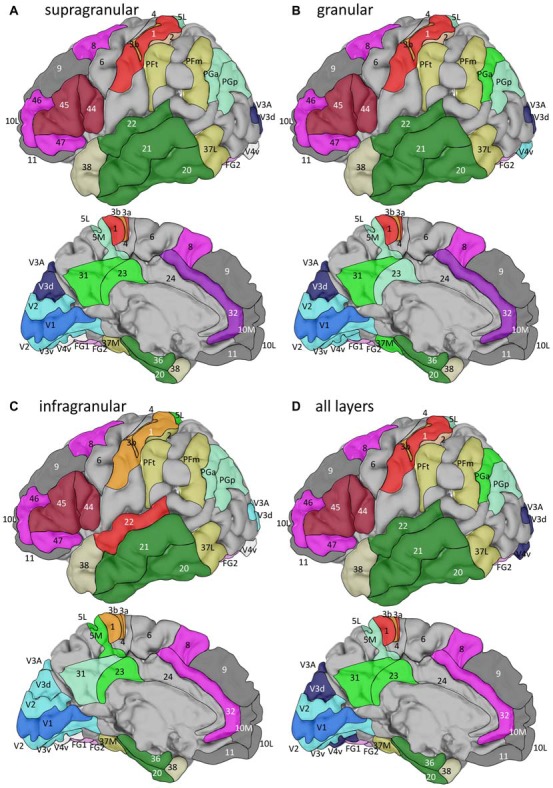
Location of the clusters with areas of similar fingerprints in the single subject template brain of the Montreal Neurological Institute. Clusters were identified by hierarchical and k-means cluster analyses (see Figure 8) of fingerprints extracted from the supragranular **(A)**, granular **(B)**, or infragranular **(C)** strata, or from all layers **(D)**. For the lateral views, anterior is on the left, and for the medial views it is on the right.

The hierarchical cluster analysis of fingerprints of mean areal receptor densities separates all early visual areas from the rest of the cortex already after the first branching in the dendrogram (Figure 8D). The highest acceptable number of clusters after k-means analysis shows that the fingerprint of the primary visual cortex differs from those of the early (V2, V3v, V3A, V3d, V4v) and higher (FG1, FG2) visual areas. The highest possible level of clustering is mapped in Figure 9D on the single subject MNI template brain. At this clustering level, the areas of the Broca region (44 and 45) form a separate cluster, as well as the parietal areas PGa, PGp, 5L, 5M with the cingulate areas 23 and 32. The temporo-polar area 38 forms a cluster by its own (Figure 9D), but is relatively similar to the multimodal temporal and inferior parietal regions as well as the temporo-occipital transition region (Figure 8D). Areas of the latter regions are found in a cluster, which comprises the temporo-occipital areas 37B, 37L and 37M as well as the inferior parietal areas PFm and PFt. Notably, the fingerprints of the primary somatosensory and primary auditory cortex cluster together. Areas 3a and 1 of the somatosensory cortex form another cluster which is separated from a cluster with somatosensory area 2 and the secondary auditory area 42. Then, all temporal isocortical areas (20–22 and 36) are found in one cluster, as well as the lateral prefrontal areas 46, 47, 8 and anterior cingulate area 32 in another cluster. Finally, the most rostral prefrontal areas 9, 10L and 10M are comprised in a cluster with the orbitofrontal area 11 (Figure 9D).

The fingerprints of the supragranular stratum (Figures 8A, 9A) again separate the visual areas from all other areas of the neo- and periarchicortex. Interestingly, V1 is again separated from the other early visual areas. Areas V3A and V3d are in one cluster, different from those of the other early visual areas. Thus, a segregation of the ventral and dorsal visual streams is supported by the multi-receptor fingerprints. All other areas show a very similar clustering as described above for the mean (all layers together) receptor fingerprints. Only PGa and PGp are found in separate clusters if the fingerprints of all layers together are analyzed, whereas both areas are in the same cluster when fingerprints from the supragranular stratum are studied.

The fingerprints of the granular stratum (Figures 8B, 9B) clearly separate the visual areas of the ventral stream from those of the dorsal stream, and also from the higher visual areas FG1 and FG2. Area V4v represented a separate cluster in the analysis of the supragranular stratum, but here in the granular stratum (Figures 8B, 9B) it clusters together with the early visual areas of the ventral stream. All other clusters contain the same areas as found in the analysis of the supragranular stratum with the exceptions of area 23 (which clusters together with 5L, 5M and PGp) and of area 37M (which clusters together with 31).

The fingerprints of the infragranular stratum (Figures 8C, 9C) do not separate the early visual areas of the ventral and dorsal streams, as found in the analyses of the supragranular and granular strata. Moreover, V4v forms a separate cluster. Considerable differences to the clustering pattern of the supragranular and granular strata are also found in the cases of the temporal area 22, the anterior cingulate area 32 and the superior parietal areas 5M and 5L as well as area 31 on the precuneus.

The cluster analyses of stratum-specific fingerprints (Figures 8, 9) highlight the special position of visual areas, particularly the early ones, since they always segregate from the remaining bulk of areas at an earlier branching level and remain separated even at the highest branching level. The fingerprints of the granular stratum most clearly support the separation of the early visual areas into dorsal and ventral streams. To a lesser extent (with the exception of V4v which forms a cluster by itself), this separation is also found in the analysis of the supragranular stratum, but not in that of the infragranular stratum.

In summary, the fingerprints of the different strata differed greatly and enabled a separation of these strata-specific fingerprints into three clusters (Figure 7). Only the agranular motor areas 4 and 6, as well as the agranular cingulate area 24 did not follow this principal segregation of the strata-specific fingerprints. Regarding the motor areas, we must conclude that a typical receptor pattern indicating the presence of an inner granular layer IV could not be demonstrated by the receptor density analyses. If the fingerprints of the three strata and that of all strata together are analyzed for all 41 granular areas, a clustering could be found which roughly follows the topographical segregation of the areas into prefrontal, parietal, temporal and occipital regions (Figures 8, 9). It is notable that the fingerprints of prefrontal multimodal association areas always cluster together and are different from those of parietal or temporal association areas. However, a deeper analysis reveals that the topographical segregation is superposed by a functional classification of areas. Hence, a further segregation of the receptor fingerprints of early visual areas into separate clusters is found in the granular and to a lesser degree also in the supragranular stratum following the concept of dorsal and ventral visual streams. Finally, the fingerprints of the supragranular and granular strata show a similar clustering pattern, whereas those of the infragranular stratum show divergent patterns in the precuneus region, the superior temporal sulcus and the anterior cingulate cortex. The analysis of the contribution of single receptor types (Figure 3) highlights the distinct increased levels of the nicotinic α_4_β_2_ receptor in the granular layers of all three primary sensory areas, and of the M_2_ receptor in the same layer of V1 and early visual areas of the ventral stream, as well as in the primary auditory cortex (area 41). In these areas, the density of the nicotinic α_4_β_2_ and the M_2_ receptors in the granular layer exceeds that of the supragranular stratum, which is the stratum with the highest receptor density in most areas. The general rule of a sequence in receptor densities from highest levels in the supragranular strata and lowest in the infragranular strata of all 44 areas is only violated by the kainate, noradrenergic α_1_ and serotonin 5-HT_1A_ receptors where the canonical sequence from highest to lowest levels is changed in some or most areas.

## Discussion

### Mean Areal Densities of Single Transmitter Receptors in the Human Cerebral Cortex

The new aspect of this study is the large scale cross area comparison of stratum-specific receptor fingerprints. It has been demonstrated that the densities of various transmitter receptors vary considerably between different cytoarchitectonically defined areas in the human cerebral cortex (Cortés et al., 1986, 1987; Hoyer et al., 1986b; Pazos et al., 1987b; Jansen et al., 1989; Zilles and Palomero-Gallagher, 2001; Zilles et al., 2004, 2015a; Morosan et al., 2005; Scheperjans et al., 2005a,b; Eickhoff et al., 2007, 2008; Palomero-Gallagher et al., 2008, 2009, 2015; Zilles and Amunts, 2009; Caspers S. et al., 2013; Vogt et al., 2013; Caspers et al., 2015; Palomero-Gallagher and Zilles, 2017a). Frequently, the densities show distinct changes at the borders between cytoarchitectonic areas, or reveal a finer parcellation of the cortex than found in cytoarchitectonic studies (Geyer et al., 1996; Amunts et al., 2010). As stated in the articles from our group, this obvious regional heterogeneity is not random, but shows systematic changes depending on the participation of cortical areas in different functional networks and their subdivisions. In the present observations, we included numerous areas for interareal comparison which were not previously studied. More importantly, the previous comparisons by cluster analyses were focussed on some specific functional networks (e.g., language-related areas, cingulate areas), thus neglecting the impact on the results of a cluster analysis caused by a greater number of cortical areas included and by a brain-wide balanced analysis. Most importantly, previous studies concentrated on mean areal densities, i.e., regional distribution patterns of receptors averaged over all cortical layers. The present study hypothesizes, that layers show different receptor balances and their fingerprints reflect the different contribution of layers to the regional fingerprints described in previous articles from our group.

As previously stated (Mash et al., 1988; Zilles et al., 2002a; Zilles, 2005), the cholinergic muscarinic M_2_ receptor consistently reaches higher densities in human and non-human primate primary visual, auditory and somatosensory areas than in hierarchically higher isocortical sensory areas or in areas of the motor cortex. This could be confirmed for V1 in the present study by new measurements at different sites in the cortical areas and by a partly new sample of brains (Figure 2). A similar outcome for previously reported noradrenergic α_2_ and serotonergic 5-HT_2_ receptors in primary sensory areas (Zilles and Palomero-Gallagher, 2017) was also observed in the present study, again using a partly new sample of brains. Accordingly, the previously mentioned principal classifications of cortical areas as primary visual, unimodal visual or higher visual cortices (Caspers et al., 2015) is confirmed by the detection of characteristic levels of densities of single or several (fingerprints) receptor types in a brain-wide analysis. Using that analysis, we could now show that NMDA, GABA_A_, GABA_A_/BZ, M_2_, α_2_, 5-HT_2_, and D_1_ receptors all reach absolute maxima in the primary visual cortex V1, whereas the same receptor types (except α_2_ and 5-HT_2_) and the M_1_ receptor reach very low densities in primary motor and premotor areas. Furthermore, the mean areal densities of GABA_A_, GABA_A_/BZ, M_2_, 5-HT_2_ and D_1_ receptors are higher in the early visual areas than in other isocortical regions. This latter finding is in sharp contrast to the regional distribution of kainate receptors, which are present at exceptionally low densities in all areas of the visual system. The multimodal association areas of the prefrontal and temporal cortices are characterized by high GABA_B_ densities above the mean of all areas. The higest AMPA and kainate receptor densities are found in areas of the prefrontal association cortex or in this region and in the temporal association cortex, respectively. Low densities of the M_2_ receptor were found in the temporal and prefrontal association cortices, whereas the nicotinic receptor reached highest mean areal densites in the prefrontal and lowest densities in the temporal association cortex. Very high α_1_ and 5-HT_1A_ receptor densities are found in the entire temporal cortex, particularly in the multimodal temporal association areas. In conclusion, primary sensory and multimodal association areas frequently showed a segregation by receptor densities. In some cases even prefrontal and temporal association areas can be separated by the distinct levels of their receptor expression. Thus, densities of single receptors are not randomly distributed over the entire cortex, but vary according to a general classification scheme into sensory, motor and multimodal areas.

### Strata Specific Densities of Transmitter Receptors in the Human Cerebral Cortex

One main focus of the present study was on the layer specificity of single receptor densities. We found a canonical sequence from high to low receptor densities when moving from the supragranular, to the granular and finally the infragranular stratum in nearly all regions. This general rule is only violated by the kainate, muscarinic M_2_, noradrenergic α_1_ and serotonin 5-HT_1A_ receptors. Kainate receptors reach their highest densities in the infragranular stratum. However, also in this case a regional segregation into visual, multimodal temporal, parietal and prefrontal association areas can be confirmed by the variable kainate receptor densities in the different cortical areas. The M_2_ receptor shows the highest and the α_1_ receptor the lowest densities in the granular stratum of numerous areas. The most extreme situation was found for the 5-HT_1A_ receptor, which reached by far highest densities in the supragranular stratum and lowest densities in the granular stratum. Very high absolute densities of this receptor were found in the supragranular stratum of temporal association areas 36 and 38 as well as of unimodal somatosensory area 2, auditory area 42 and anterior cingulate area 24.

**Figure 10:**
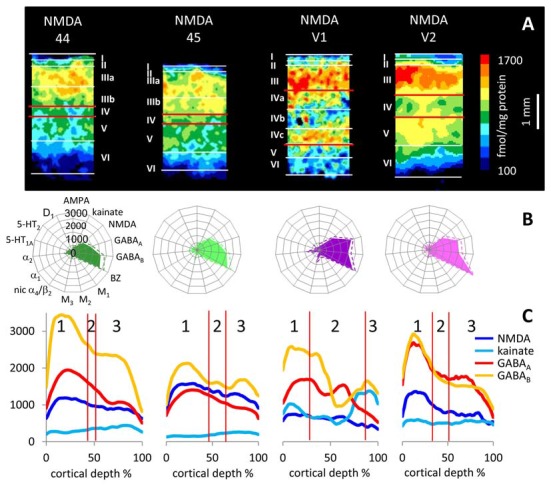
Laminar distribution of the NMDA receptor in human primary (V1) and secondary (V2) visual cortex as well as in areas 44 and 45 of the Broca region. **(A)** Color coded autoradiographs. Scale bar codes for receptor densities in fmol/mg protein. **(B)** Multi-receptor fingerprints. **(C)** Receptor density profiles. 1 supragranular stratum, 2 granular stratum, 3 infragranular stratum. The laminar borders have been defined by comparison with neighboring cell-body stained (cytoarchitecture) sections. Cortical depths of the different areas are normalized to 100%. *Y* axis codes for receptor densities in fmol/mg protein.

In accordance with previous reports (e.g., Rakic et al., 1988; Young et al., 1990), NMDA receptor binding was found to be most dense in the supra- and granular strata of cerebral cortex. AMPA receptor densities were highest in layers II and III of primary visual cortex and relatively low in layer IV (see also Rakic et al., 1988; Carlson et al., 1993). Although many AMPA and NMDA receptors are localized at thalamo-cortical and cortico-cortical synapses, the co-localization of these receptors with interneurons is of special importance for the analysis of local signal processing within cortical microcircuits and layers. The co-localization of AMPA and NMDA receptor subunits with calcium binding protein expressing inhibitory interneurons was immunohistochemically studied in macaque primary visual cortex (Kooijmans et al., 2014; Kooijmans, 2016). The co-localizations of parvalbumin with GluA1 or GluA4 AMPA receptor subunits in the fast spiking chandelier and basket cells was found to be minor, in contrast to a higher co-localization of parvalbumin and GluA2 or GluA3. Calbindin and GluA2 or GluA3 AMPA receptor subunits are co-localized to a minor degree, but calbindin and GluA1 or GluA4 show a higher degree in the intermediate spiking neurogliaform and Martinotti cells. Co-localization of calretinin with GluA2 or GluA3 is minor, but more was found between calretinin and GluA1 or GluA4 AMPA receptor subunits in the intermediate spiking double bouquet cells. Calcium binding protein expressing interneurons also synthesize NMDA receptors, which consist of two GluN1 and two GluN2 subunits in most cases (Kooijmans, 2016). Therefore, a co-localization analysis of the four isoforms of the GluN2 receptor with the three classes of calcium binding protein containing interneurons provides insight into the cellular localization of the NMDA receptor both on its interneuron-type specific and laminar-specific distribution. Parvalbumin neurons preferentially express only a few NMDA receptors, whereas the calbindin and calretinin neurons show a much higher co-localization with GluN2 NMDA receptor subunits (Kooijmans, 2016). If we compare the laminar patterns of the GluN2 receptor in the neuropil with the present autoradiographic observations of the NMDA receptors, a conspicuous similar laminar pattern can be seen. Summarizing the NMDA and AMPA receptors, our data (for NMDA receptors see Figure 10; Supplementary Table S2) show that the laminar patterns of NMDA and AMPA receptors in the human cortex are comparable to that previously reported in both primary sensory and motor cortices of Old World macaques (Young et al., 1990; Geyer et al., 1998; Garraghty et al., 2006).

**Figure 11:**
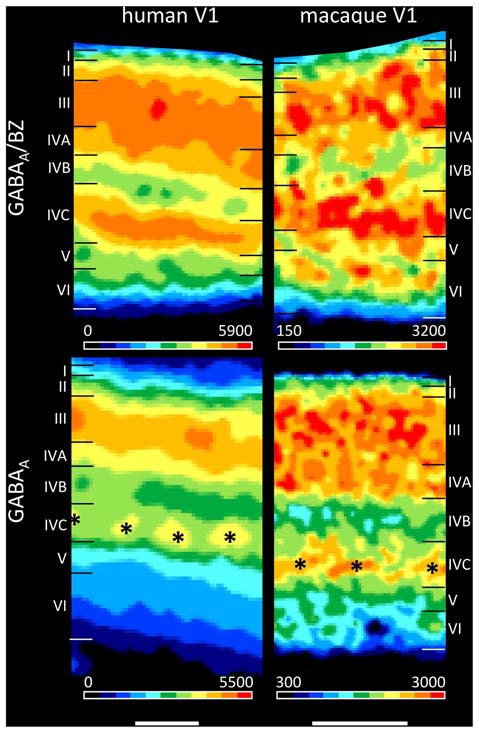
Laminar distribution of the GABA_A_/BZ binding sites and the GABA_A_ receptor in human and macaque primary visual cortex V1. Color bars code for receptor densities in fmol/mg protein. Scale bars 1 mm. Asterisks highlight the portion of layer IV with higher GABA_A_ receptor densities in macaque than in human V1.

The present findings on the laminar distribution of GABA_A_ receptors labeled with [^3^H] muscimol in human area V1 displayed a high density of this receptor in layer IVC that stood out due to the considerably lower densities in layers IVB and V. Notable is the patchy appearance of layer IVC with distributed GABA_A_ receptor maxima along this layer. This is visible both in human and macaque V1 (Figure 11), and may be indicating the modular organization of this layer (Hubel and Wiesel, 1968, 1969; Wiesel et al., 1974). Supragranular layer II has a moderate density and layers III–IVA a high density of GABA_A_ receptors. The infragranular layers V–VI show densities comparable or even lower than in layer I. This pattern of human V1 is well comparable with that in the macaque (Figure 11 and Rakic et al., 1988).

We found a bilaminar distribution pattern of the GABA_A_/BZ binding sites in human area V1 with the highest density in layer IVC, somewhat lower but still high densities in layers III–IVA, and the lowest densities in layers IVB and V–VI (Figure 11). This is comparable with autoradiographic measurements in V1 of macaque monkeys (Rakic et al., 1988), with the exception of the intermediate to high density in the upper part of macaque layer VI. To resolve the divergent finding of this layer VI band in macaque in contrast to human V1, where it is not visible, we labeled the GABA_A_/BZ binding sites in V1 of both species. Figure 11 shows that indeed the thin band with high binding site density in layer VI as described by Rakic et al. (1988) is caused by a species difference, since it is present in macaque but not in human V1.

The calcium-binding proteins parvalbumin, calbindin and calretinin label almost exclusively inhibitory GABAergic interneurons in the macaque visual cortex (Van Brederode et al., 1990; DeFelipe et al., 1999a; Disney and Aoki, 2008; Kooijmans et al., 2014). These interneurons with their rich axonal arborization account for more than 90% of the inhibitory cells in the primate cerebral cortex (DeFelipe et al., 1999a; Disney and Aoki, 2008), and are layer-specifically distributed (Van Brederode et al., 1990; Shaw et al., 1991; Lund and Wu, 1997; Disney and Aoki, 2008; Kooijmans et al., 2014). Since GABAergic signal processing requires GABA receptors, and GABAergic interneurons often synapse in their near surrounding (Lund and Wu, 1997), the preferential localization of GABAergic interneurons in the supragranular and granular strata (Fitzpatrick et al., 1987; Lund, 1987; Lund et al., 1988; Lund and Yoshioka, 1991; Lund and Wu, 1997) may be correlated with that of GABA receptors. The present laminar-specific data on GABA_A_ and GABA_A_/BZ binding sites in the supragranular and granular strata clearly supports this hypothesis (Figures 10C, 11). The particularly large divergence of the laminar densities of these receptor types between the supragranular and granular strata of V1 (high densities) on the one hand, and its infragranular stratum (low densities) on the other sites demonstrates the preferential laminar localization of these inhibitory receptor types in the primate cortex (also see Figure 3).

The inhibitory GABA_A_ receptors are activated by the agonist muscimol. [^3^H]muscimol binds at the orthosteric α1/β2 subunit interface and interacts with the receptor via a high-affinity binding site. The α1 subunit has a strong influence on all binding properties, including desensitization, while the β2 subunit has minor impact (Baur and Sigel, 2003). The subunit composition of the functional GABA_A_ receptor in the cerebral cortex is almost unknown, but it has been shown that probably all GABA_A_ receptors contain α1, β and γ subunits (Huntsman et al., 1994). The α1 subunit of the GABA_A_ receptor is the most frequent subunit. The highest density of this subunit was found in layer IVC of the primary visual cortex, while layers II–III and IVA show a homogeneous somewhat lower density than layer IVC. The distribution of the β2 subunit is similar to that of the α1 subunit. Layers I and IVB have low, layers II–III moderate, and layers IVA and IVC high densities of the β2 subunit. Sublayer IVCβ shows a very high density in its upper part and lower density in its deeper part. Layers V and VI have very low densities, with only slightly higher values in layer V (Huntsman et al., 1994). The laminar distribution of α1 and β2 subunits is therefore comparable to the strata-specific distribution of the [^3^H] muscimol binding to the GABA_A_ receptor as found in the present observations. In area 3b of the primary somatosensory cortex, the present findings of the laminar distribution of GABA_A_ and GABA_B_ receptors (highest in superficial layers) are also consistent with previous reports in the macaque monkey (Rakic et al., 1988; Shaw et al., 1991).

The α_1_ receptor reaches highest densities in layers I–II of human V1, moderate values in upper layer III. Lowest receptor densities are found in layers IVA–IVC and VI. Layer V shows a density intermediate between layers III and VI. The macaque V1 shows a very similar laminar distribution pattern (Rakic et al., 1988). The 5-HT_2_ receptors reach relatively high densities in layers III and IVa of human V1, followed by a slightly lower but still high density in layer IVC. Thus, two bands of high receptor densities are present in human V1. Lowest values are found in layers V–VI, whereas layers I–II and IVB have moderate densities. These findings are comparable to the laminar pattern in macaque V1 (Rakic et al., 1988).

Whereas most receptors show a parallel course of the mean areal densities and the layer-specific densities, the nicotinic α_4_β_2_ receptor is a notable exception. In all three primary sensory areas, this receptor displayed distinctly higher maxima in the granular stratum exceeding those of other strata and of mean areal densities (Figure 3). Thus, the predominant input layer of primary sensory cortices seems to be under a strong modulatory influence of this cholinergic receptor type. The granular strata of some visual areas also had higher M_2_, α_2_ and 5-HT_2_ receptor densities than the supragranular strata. This complements from a molecular point of view the exceptional cytoarchitectonic differentiation and thickness of the granular layer in the visual cortical areas of the human cortex, particularly in V1. Furthermore, our present finding of generally higher receptor densities in the supragranular than in the infragranular layers of most cortical areas is supported by a comparable laminar relation of synapse numbers (Huttenlocher and Dabholkar, 1997; DeFelipe et al., 1999b), if the density of all receptors in a cortical area and layer are correlated with the number of synapses (for further details see “Discussion” in “Sizes of Absolute Receptor Fingerprints Reveal Regional and Laminar Heterogeneity of Receptor Densities” Sections, and Rakic et al., 1986, 1994).

### Multi-Receptor Fingerprints of Cortical Areas and Layers Reflect Principle Aspects of Functional Organization

Since all cortical layers expressed all receptor types studied here, we analyzed the aspect of multi-receptor expression by calculating receptor fingerprints, a major tool for characterizing the balance between receptor expressions in the different strata. Here, the densities of 15 different receptor types in each of the 44 areas with a brain-wide distribution were visualized separately for each stratum. The discriminant analyses demonstrated the regional inhomogeneity of the fingerprints averaged over all layers and separately for each of the three strata. The results of the subsequent pairwise comparisons between areas, which resulted in significant differences only for a subsample of pairs, is not surprising because only three brains could be included in the present study of 15 different receptors in 44 cortical areas and their three strata. An enlargement of the sample of brains is currently not possible due to practical limitations (shortage of adequate human brain tissue with short post mortem delay, technical difficulties associated with the serial sectioning of entire deep frozen and unfixed human hemispheres and the financial requirements for autoradiographical processing of thousands of sections). The size of the fingerprints, scaled by absolute mean areal or stratum-specific densities, reflects the sum of the densities of all receptors, and shows considerable regional variations. Since the absolute densities of AMPA, NMDA, GABA_A_, GABA_A_/BZ and GABA_B_ receptors are much higher than those of all other receptor types, we additionally calculated normalized fingerprints. These normalized fingerprints also show a regional heterogeneity of multi-receptor expression both for the mean areal densities and the stratum-specific densities.

#### Sizes of Absolute Receptor Fingerprints Reveal Regional and Laminar Heterogeneity of Receptor Densities

The size of the absolute fingerprint of a cortical area is defined by the sum of the densities of all receptors measured in that area. Since we measured all receptor types in all areas, and the absolute fingerprints are identically scaled, the sizes of fingerprints enable a comparison of the densities (fmol mg/protein) between all 44 areas, and reveal area- as well as strata-specific differences, thus demonstrating the regional heterogeneity of receptor expression (Figure 4). In the supragranular stratum, the sum of the densities of all receptors is consistently larger throughout all areas than that of the granular stratum. The infragranular stratum reaches the lowest value. The largest fingerprints are found in the primary, secondary, and early visual areas, as well as in temporal and parietal multimodal association areas and area 11 of the orbitofrontal cortex, particularly in the supragranular stratum. The size of the fingerprints of the infragranular stratum is very low in the primary visual cortex, which stands out by an extremely large size of the fingerprint in its supragranular stratum. This is in contrast to the supra- vs. infragranular relation of the fingerprint sizes in the areas of the motor cortex and the Broca region, since these regions have very small sized supragranular fingerprints. In conclusion, the relation of the densities of all receptors can vary between supragranular and infragranular strata in a regionally and functionally dependent manner. The independent variation of receptor expression between both strata is further supported by the shape of the fingerprints, which addresses the balance between receptor types in a given area (see below).

The smallest fingerprints are found in the primary motor cortex area 4 (as previously described in Zilles et al., 2015a) and motor area 6. Accordingly, by also studying the premotor cortex, our previous conclusion based on a multi-receptor fingerprint analysis in the primary motor cortex of a partly different brain sample can now be generalized for all motor areas, which show the lowest level of the sum of *all* receptor densities studied in the 44 cortical regions. The motor areas are followed by areas 44 and 45 of the Broca region. These results of a multi-receptor fingerprint analysis emphasize a special position of these language-related areas between language and motor function.

Interestingly, the motor and Broca areas are both highly myelinated in the adult brain (Hopf, 1956), thus potentially causing a higher quenching of the tritium-based β-radiation (Rakic et al., 1988; Zilles et al., 1990), which may lead to an underestimation of actual receptor densities in these areas. However, highest myelination levels were reported for area 41 and 42 and the fusiform gyrus, and much lower levels for area 38 and 20 (Hopf, 1955), but the size of fingerprints of these areas shows a sequence from large to small sizes of fingerprint which does not reflect the myelination degree; e.g., area 38 has small areal and laminar fingerprint sizes, but a low myelination level. Furthermore, the sparsely myelinated area 20 has a fingerprint size above the average. Moreover, the fingerprint size of area 20 is well comparable to that of the highly myelinated areas 41 and 42. Therefore, the regional myelination levels vary independently from those of receptor densities. Since myelinated fibers do not have synaptic contacts on the surface of the myelin sheaths, and therefore do not express transmitter receptors along their course through a cortical area, a higher portion of cortical tissue in these areas is free of receptors compared to areas with lower content of myelinated fibers. This may better explain the regional and laminar heterogeneity of the sizes of absolute fingerprints and the low levels total sum of the densities of all receptors in these particular regions than a general quenching effect. The density in the granular stratum of V1 is averaged over the sublayers IVa–IVc, including the heavily myelinated layer IVb. Thus, the issue of quenching is also relevant for all data from the granular stratum of V1, and may be underestimated. However, the potential quenching effect apparently did not severely influence the clustering of the granular stratum of V1 as a whole, because layer IVb contributes by only about a third of the size of the granular stratum, and it is found in the same cluster as other granular stratum-specific data in all other cortical areas with a distinct layer IV.

In general, unimodal sensory areas show a higher inter-laminar divergence of total receptor densities than multimodal association areas. This suggests a greater difference of receptor-mediated processing mechanisms between the cortical layers in the unimodal sensory areas, particularly in the primary visual cortex, compared to multimodal association areas.

The principal differences in the total densities of all receptors in a cortical area may be associated with the different prevailing connections of cortical layers. Whereas the supragranular strata are preferred by cortical-cortical connections, the granular stratum is that of thalamo-cortical and lateral connections, and the infragranular stratum gives raise mainly to subcortical projections (Rockland and Pandya, 1979; Kennedy and Bullier, 1985; Felleman and Van Essen, 1991; Rockland and Van Hoesen, 1994; Rockland, 1997, 2015; Hupé et al., 1998; Barone et al., 2000; Ekstrom et al., 2008; Sincich et al., 2010; Markov and Kennedy, 2013; Markov et al., 2014). Thus, these different connectivity patterns may be paralleled by different receptor expression levels in the cortical strata. Furthermore, the branching of the apical dendrites is maximal in the supragranular stratum compared to the other strata (Lund et al., 1981; Katz, 1987; Hübener et al., 1990). The number of synapses shows a comparable trend (Rakic et al., 1986, 1994). The similar laminar distribution of the number of synapses and the density of receptors is further supported by reports which demonstrated their parallel development during brain maturation (Rakic et al., 1986, 1994). Thus, the stratum-specific proportion between the sizes of receptor fingerprints may also indicate a comparable laminar distribution of the total number of synapses in a given cortical area.

#### Shape of Receptor Fingerprints

In contrast to the absolute size of fingerprints, which is caused by the total density of all receptors in a given area, the shape of a fingerprint reflects the balance between the different receptors in an area. Since the absolute fingerprints are based on the same scaling of the densities of all receptors, the peculiarities of modulatory receptors are difficult to recognize by visual inspection, because their absolute densities are lower than those of the GABAergic receptors, sometimes by two orders of magnitude. Therefore, we additionally calculated normalized fingerprints.

E.g., the high M_2_ receptor density compared to those of all other receptors in the visual cortex, particularly in V1, is reflected by the shapes of their normalized fingerprints. i.e., V1 contains the highest M_2_ receptor density of all areas examined here. Thus, the impact of the M_2_ receptor on the specific shape of a fingerprint, and possibly on the balance between all receptors, is relatively the highest in V1. Also the contribution of α_2_ receptors on the shape of the fingerprint clearly stands out in all primary sensory areas when compared to the other receptors in these areas. Additionally, the above mentioned high absolute density of the nicotinic α_4_β_2_ receptor in the granular stratum of the primary somatosensory and auditory areas is well recognizable in the normalized fingerprint. Another example is also the high relative density of α_1_ receptors in all three strata of the premotor cortex. This suggests that the influence of this receptor is the highest in this area compared to the other areas examined here. A further exceptional position is observed for the M_3_ receptor in the inferior parietal area PGp (Supplementary Figure S2). In conclusion, the normalized fingerprints clearly demonstrate locally specific roles of certain receptor types which lead to different balances between the receptors in the here studied areas and possibly to such different balances in all other regions of the cerebral cortex. That is a hint to a regional specificity of the receptor-mediated mechanisms of information processing.

Since both the different sizes and shapes of fingerprints reflect the regional and stratum-specific balances between receptors, a multidimensional scaling analysis of the fingerprints was performed. It shows that each stratum and each area have a characteristic fingerprint and thus, specific levels of total receptor densities and balances between the receptors (Figure 5). This analysis shows that the fingerprints of the three strata of all areas form three different clusters. This result supports our interpretation above that the stratum-specific information processing is based on different balances between the receptors. The separation between the clusters of the three strata is nearly perfect, and their fingerprints do not overlap in the multidimensional scaling analysis.

Only exceptions from this general finding are the positions of the fingerprints of the granular stratum in motor areas 4 and 6, which are shifted into the range of the infragranular cluster, and the concomitant shift of the supragranular fingerprints into the granular cluster. This shift of stratum-specific fingerprints of areas 4 and 6 to the “wrong” clusters may be caused by the method with which we defined the position of their layer IV (see “Materials and Methods” Section). In the vast majority of the literature, areas 4 and 6 are described as being agranular, i.e., lacking a layer IV (Brodmann, 1909). However, recent observations using Nissl and immunohistochemical stainings demonstrated the occurrence of layer IV cells in the primary motor cortex of rhesus monkeys (García-Cabezas and Barbas, 2014; Barbas and García-Cabezas, 2015), and led to the statement that motor areas do not lack a layer IV. Since we could not identify by visual inspection a clearly recognizable layer IV in either area, we tried to define such a layer at the border between layers III and V by its topography as a band occupying 3% of the total cortical depth. This width was derived from data of von Economo and Koskinas (1925), who described the width of layer IV as being approximately this size in the rostrally adjacent frontal areas. This formal definition of a layer IV in the motor areas is, however, different from the identification of such a layer in all other areas (except for area 24) examined here, where we can clearly detect a thinner or broader (granular cortex) layer of small round cells (“granular” cells) not, or only to a minor degree, intermingled with pyramidal cells (dysgranular cortex). Since the fingerprints of supragranular and granular strata of areas 4 and 6 are shifted to the clusters of the granular and infragranular strata, respectively, our multidimensional scaling analysis is thus a hint to a receptor expression pattern of the granular stratum in these areas completely different from the patterns in other cortical regions. Thus, the question remains whether this is a specificity of the motor areas, or these areas do not have a granular stratum comparable to those of all other isocortical regions. The supragranular fingerprints of areas 44 and 45 are also shifted into the cluster of the fingerprints of the granular stratum, though only slightly.

The separation of the layer-specific fingerprints further supports the existence of the above discussed divergence of receptor supported processing mechanisms between the three strata. The principal separation of the layers by their receptor fingerprints does not exclude a vertically organized interaction between the layers according to the concept of functional columns. Rather, it emphasizes that the stratum-specific receptor balances occurring at the different levels of a column are embedded in a vertically organized interaction which enables the area-specific functions.

The degree of similarity between the fingerprints of the three strata and of all layers together was analyzed by hierarchical cluster analyses which revealed a considerable regional heterogeneity, but also some notable general rules. Early visual areas were clearly separated from the rest of the cortex if we focus on the supragranular stratum and on all layers together, thus emphasizing similar receptor balances in this stratum over all these areas. The analysis of the fingerprints of the granular stratum emphasizes the exceptional organization of layer IV in V1 and the similaritiy of these fingerprints in the ventral visual stream in contrast to the dorsal visual stream, since areas V3d and V3A are clearly separated from the cluster of the ventral stream areas. The infragranular stratum does not separate dorsal and ventral stream areas from each other. Therefore, we can conclude, that organizational principles of the visual cortex (primary vs. higher unimodal sensory areas; ventral vs. dorsal stream areas) are recognizable by the fingerprints, but to different degrees in the different strata. A further general finding was the close similarity of the receptor fingeprints of the primary and secondary somatosensory and auditory areas. This is most clearly seen in the supra- and infragranular strata, as well as in the fingerprints of all layers together. This leads again to the conclusion that primary and secondary sensory areas are clearly different from higher unimodal or multimodal cortices by their receptor balances. As discussed above, specific receptor types (M_2_, nicotinic α_4_β_2_, noradrenergic α_2_, and serotonergic 5-HT_2_) play an important role here for the special position of particularly primary secondary areas. Although the function of the single receptor types has been intensely studied, the here important aspect of the role of the different receptor types within the cortical microcircuitry is, however, presently largely unknown. Our results on strata- and area-specific fingerprints and their relationships to general classification schemes of the cortex may be seen as a stimulus to study the specific functional role of these recepotr types in a systemic environment. Beside the distinct position of primary and early sensory unimodal areas, it must be emphasized that also the separation of the cluster of all prefrontal areas (analyses of all strata and all layers together) from other multimodal association areas with separate clusters for the infraparietal and temporal regions is a strong hint to the analytical potential of the hierarchical receptor fingerprint analysis as a tool to understand principal rules of cortical segregation also in higher functional and multimodal systems.

It is remarkable that the receptor fingerprints of the primary somatosensory area 3b and the primary auditory cortex (area 41) are very similar and cluster together, while both fingerprints largely differ from that of the primary visual cortex V1. Therefore, fingerprints seem to reflect differences in modalities of sensory systems. The similarity of the fingerprints of areas 41 and 3b may be explained by their similar functional properties, i.e., mechanoreception, whereas the input in V1 is clearly different. Thus, V1 has to comply with different functional requirements compared to the auditory and somatosensory systems. Comparable differences can also be found for the unimodal sensory areas and their fingerprints. The fingerprint of area 2 (somatosensory cortex) is very similar to that of area 42 (secondary auditory cortex), and both clearly differ from the fingerprint of the secondary visual cortex V2. Finally, the fingerprints of areas 44 and 45, which are subdivisions of Broca’s language region, consistently cluster together in all strata and in the entire cortical width. All these findings suggest a modality-specific component of the shape of receptor fingerprints.

The different levels of branching are indicated in the hierarchical cluster analyses. The highest possible number of clusters after k-means analysis was determined. All areas belonging to the same cluster in the supragranular, granular or infragranular strata, as well as to a cluster defined at the level of the mean over all layers are then labeled with the identical color. The resulting map indicates a subdivision of the cortex based on the similarity or dissimilarity of the regional-specific fingerprints in the different strata or entire areas. These maps are remarkable similar, since they assign in most cases the same areas to one cluster irrespective of the stratum in which the fingerprints have been yielded. Notably, the clusters comprise neighboring areas in many cases, but it must be emphasized, that the criterion of topographical neighborhood expresses at the same time a grouping of the areas according to different modalities or principal classifications into primary sensory, motor or higher multimodal association areas. Interestingly, area 38 does not cluster with the other multimodal temporal areas, but with areas on the supramarginal gyrus (PFm, PFt) and with the temporo-occipital transition zone (areas 37B, 37L, 37M). The latter areas have been attributed to the language network of Wernicke’s region (Mesulam et al., 2015). Therefore, the clustering of the fingerprint of area 38 with other language regions may be explained by the special position of this area within the larger language system based on its role in object naming (Mesulam et al., 2013). Also areas 44 and 45 of the Broca region are consistently found in an own cluster, and segregate from neighboring areas of the premotor, lateral prefrontal, orbitofrontal and frontopolar cortices. A clear segregation is also found between anterior cingulate areas 32 and 24, which are often merged in functional imaging studies. The fingerprints clearly argue against this merging, this is corroborated by more detailed cyto- and receptorarchitectonic studies (Palomero-Gallagher et al., 2008). Finally, the higher visual areas FG1 and FG2 are found in separate clusters compared to the early visual areas. This supports the results of an early study focused on these two areas of the fusiform gyrus (Caspers et al., 2015) at the level of single strata.

In conclusion, the present results provide evidence for the fact that the regional and laminar heterogeneity of multi-receptor expression patterns in the cerebral cortex is not random. Rather, transmitter receptor densities vary sytematically between cortical areas depending on the functional networks, or subdivisions thereof, to which they can be assigned. Furthermore, a general canonical sequence of densities from highest values in the supragranular stratum, intermediate values in the granular layer IV, and lowest values in the infragranular stratum is found in most areas, and for most receptor types. The stratum-specific differences in the patterns of multi-receptor balances, point at divergent receptor supported processing mechanisms between the three strata. Finally, area- and stratum-specific multi-receptor expression patterns (i.e., receptor fingerprints) reflect the segregation of the cerebral cortex into functionally and topographically definable groups of cortical areas (visual, auditory, somatosensory, limbic, motor), and reveal their hierarchical position (primary and unimodal (early) sensory to higher sensory and finally to multimodal association areas) within sensory systems.

## Ethics Statement

This study was carried out in accordance with the recommendations of “Experimentelle wissenschaftliche Studien an Gewebeproben von Gehirnen und Organen von Körperspendern, Ethics Committee of the Medical Faculty of the Heinrich-Heine University Düsseldorf” with written informed consent from all subjects. All subjects gave written informed consent in accordance with the Declaration of Helsinki. The protocol was approved by the “Ethics Committee of the Medical Faculty of the Heinrich-Heine University Düsseldorf.”

## Author Contributions

KZ and NP-G conceived of and designed the study, analyzed data, drafted the manuscript and figures. NP-G acquired data. KZ obtained funding.

## Conflict of Interest Statement

The authors declare that the research was conducted in the absence of any commercial or financial relationships that could be construed as a potential conflict of interest.
