# Supplementary material for: Multiple Transmitter Receptors in Regions and Layers of the Human Cerebral Cortex
Source: Front Neuroanat. 2017 Sep 20;11:78. doi: 10.3389/fnana.2017.00078 (PMC5609104; doi:10.3389/fnana.2017.00078)
Supplement: Supplementary file 1 [file Table_1.pdf]

Supplementary Table 1:

Summary of incubation conditions for receptor autoradiography. * substance only present in the main incubation buffer.

Transmitter	Receptor (characteristics)	Ligand (pharmacological class) [nM]	Displacer [µm]	Incubation buffer	Pre-incubation	Main incubation	Final rinsing
Glutamate	AMPA (ionotropic, excitatory)	[^{3}H]-AMPA (agonist) [10.0]	Quisqualate [10]	50mM Tris-acetate (pH 7.2) + 100 mM KSCN*	3x 10 min, 4° C	45 min, 4° C	1) 4x 4sec 2) Acetone/glutaraldehyde (100 ml + 2,5 ml), 2x 2sec, 4° C
	NMDA (ionotropic, excitatory)	[^{3}H]-MK-801 (antagonist) [3.3]	(+)MK-801 [100]	50mM Tris-acetate (pH 7.2) + 50 µM glutamate +30 µM glycine* + 50 µM spermidine*	15 min, 4° C	60 min, 22 ° C	1) 2x 5min, 4° C 2) distilled water, 1x, 22 ° C
	Kainate (ionotropic, excitatory)	[^{3}H]-Kainate (agonist) [9.4]	SYM 2081 [100]	50mM Tris-acetate (pH 7.1) +10 mM Ca^{2+}-acetate*	3x 10 min, 4° C	45 min, 4° C	1) 3x 4sec 2) Acetone/glutaraldehyde (100 ml + 2,5 ml), 2x 2sec, 22 ° C
GABA	$GABA_A$ (ionotropic, inhibitory)	[^{3}H]-Muscimol (agonist) [7.7]	GABA [10]	50mM Tris-citrate (pH 7.0)	3 x 5 min, 4° C	40 min, 4° C	1) 3x 3 sec, 4° C 2) distilled water, 1x, 22 ° C
	$GABA_A$/BZ (ionotropic, inhibitory)	[^{3}H]-Flumazenil (antagonist) [1.0]	Clonazepam [2]	170 mM Tris-HCl (pH 7.4)	15 min, 4° C	60 min, 4° C	1) 2x 1 min, 4° C 2) distilled water, 1x, 22 ° C
	$GABA_B$ (metabotropic, inhibitory)	[^{3}H]-CGP 54626 (antagonist) [2.0]	CGP 55845 [100]	50mM Tris-HCl (pH 7.2) + 2,5 mM $CaCl_2$	3 x 5 min, 4° C	60 min, 4° C	1) 3x 2 sec, 4° C 2) distilled water, 1x, 22 ° C
Acetylcholine	M_1 (metabotropic, excitatory)	[^{3}H]-Pirenzepine (antagonist) [1.0]	Pirenzepine [2]	Modified Krebs buffer (pH 7.4)	15 min, 4° C	60 min, 4° C	1) 2x 1 min, 4° C 2) distilled water, 1 x, 22 ° C
	M_2 (metabotropic, inhibitory)	[^{3}H]-Oxotremorine-M (agonist) [1.7]	Carbachol [10]	20 mM HEPES-Tris (pH 7.5) + 10 mM $MgCl_2$ + 300 nM Pirenzepine	20 min, 22° C	60 min, 22° C	1) 2x 2 min, 4° C 2) distilled water, 1 x, 22 ° C
	M_3 (ionotropic, excitatory)	[^{3}H]-4-DAMP (antagonist) [1.0]	Atropine sulfate [10]	50 mM Tris-HCl (pH 7.4) + 0.1 mM PSMF + 1mM EDTA	15 min, 22° C	45 min, 22° C	1) 2x 5 min, 4° C 2) distilled water, 1 x, 22 ° C
	nicotinic $\alpha_4\beta_2$ (ionotropic, excitatory)	[^{3}H]-Epibatidine (agonist) [0.5]	Nicotine [100]	15mM HEPES (pH 7.5) + 120 mM NaCl + 5.4 mM KCl + 0.8 mM $MgCl_2$ + 1.8 mM $CaCl_2$	20 min, 22° C	90 min, 22° C	1) 5 min, 4° C 2) distilled water, 1 x, 22 ° C

Noradrenaline	α_1 (metabotropic, excitatory)	[^{3}H]-Prazosin (antagonist) [0.2]	Phentolamine Mesylate [10]	50mM Na/K-phosphate buffer (pH 7.4)	15 min, 22° C	60 min, 22° C	1) 2x 5 min, 4° C 2) distilled water, 1 x, 22 ° C
	α_2 (metabotropic, inhibitory)	[^{3}H]-UK 14,304 (agonist) [0.64]	Phentolamine Mesylate [10]	50 mM Tris-HCl (pH 7.7) + 100 μM $MnCl_2$	15 min, 22° C	90 min, 22° C	1) 5 min, 4° C 2) distilled water, 1 x, 22 ° C
Serotonin	5-HT_{1A} (metabotropic, inhibitory)	[^{3}H]-8-OH-DPAT (agonist) [1.0]	5-Hydroxy-tryptamine, [1]	170 mM Tris-HCl (pH 7.4) +4 mM $CaCl_2$* + 0.01% ascorbate*	30 min, 22° C	60 min, 22° C	1) 5 min, 4° C 2) distilled water, 3 x, 22 ° C
	5-HT_2 (metabotropic, excitatory)	[^{3}H]-Ketanserin (antagonist) [1.14]	Mianserin [10]	170 mM Tris-HCl (pH 7.7)	30 min, 22° C	120 min, 22° C	1) 2x 10 min, 4° C 2) distilled water, 3 x, 22 ° C
Dopamine	D_1 (metabotropic, excitatory)	[^{3}H]-SCH 23390 (antagonist) [1.67]	SKF 83566 [1]	50 mM Tris-HCl (pH 7.4) + 120 mM NaCl + 5 mM KCl + 2 mM $CaCl_2$ + 1 mM $MgCl_2$	20 min, 22° C	90 min, 22° C	1) 2x 20 min, 4° C 2) distilled water, 1 x, 22 ° C
