# Supplementary material for: Multiple Transmitter Receptors in Regions and Layers of the Human Cerebral Cortex
Source: Front Neuroanat. 2017 Sep 20;11:78. doi: 10.3389/fnana.2017.00078 (PMC5609104; doi:10.3389/fnana.2017.00078)
Supplement: Supplementary file 2 [file Table_2.pdf]

Supplementary Table 2:
Receptor densities in the supragranular, granular and infragranular strata as well as averaged over all layers in the 44 cortical areas. Primary sensory areas are highlighted in yellow, the primary motor cortex in grey. Data is presented a mean densities (in fmol/mg protein) over all examined areas, standard deviation (SD) and variation coefficients (VC)

	AMPA											
**Region**	**supragranular**	**(SD)**	***CV***	**granular**	**(SD)**	***CV***	**infragranular**	**(SD)**	***CV***	**all layers**	**(SD)**	***CV***
**1**	518	(220)	*0.424*	346	(152)	*0.440*	354	(156)	*0.442*	443	(191)	*0.430*
**2**	549	(228)	*0.416*	424	(169)	*0.400*	365	(136)	*0.373*	478	(197)	*0.412*
**3a**	564	(164)	*0.291*	389	(110)	*0.281*	334	(93)	*0.279*	473	(145)	*0.306*
**3b**	538	(216)	*0.402*	350	(144)	*0.412*	324	(133)	*0.411*	455	(179)	*0.393*
**V1**	606	(344)	*0.567*	406	(169)	*0.415*	276	(121)	*0.439*	496	(274)	*0.412*
**V2d**	569	(290)	*0.509*	373	(116)	*0.310*	333	(136)	*0.407*	451	(206)	*0.309*
**V2v**	605	(177)	*0.293*	416	(18)	*0.044*	350	(23)	*0.064*	472	(58)	*0.287*
**V3A**	639	(271)	*0.424*	414	(110)	*0.266*	425	(56)	*0.133*	534	(169)	*0.365*
**V3d**	583	(245)	*0.420*	380	(139)	*0.367*	324	(130)	*0.401*	478	(198)	*0.347*
**V3v**	611	(201)	*0.330*	414	(49)	*0.118*	333	(106)	*0.319*	477	(118)	*0.265*
**V4v**	507	(56)	*0.111*	376	(6)	*0.016*	271	(53)	*0.194*	383	(58)	*0.309*
**FG1**	740	(56)	*0.076*	548	(24)	*0.043*	504	(29)	*0.058*	623	(41)	*0.066*
**FG2**	780	(122)	*0.156*	620	(12)	*0.020*	552	(56)	*0.101*	656	(66)	*0.101*
**37B**	582	(162)	*0.278*	485	(137)	*0.282*	478	(138)	*0.289*	533	(148)	*0.278*
**37L**	564	(181)	*0.116*	444	(151)	*0.196*	438	(151)	*0.203*	506	(170)	*0.149*
**37M**	586	(271)	*0.227*	460	(209)	*0.212*	446	(206)	*0.192*	513	(240)	*0.213*
**41**	561	(187)	*0.333*	368	(124)	*0.338*	367	(119)	*0.326*	479	(164)	*0.342*
**42**	568	(179)	*0.315*	419	(123)	*0.293*	395	(103)	*0.260*	498	(152)	*0.304*
**20**	624	(285)	*0.316*	570	(247)	*0.423*	537	(248)	*0.471*	584	(259)	*0.366*
**21**	669	(311)	*0.465*	590	(268)	*0.454*	554	(259)	*0.468*	618	(289)	*0.468*
**22**	583	(332)	*0.570*	444	(219)	*0.492*	408	(179)	*0.439*	506	(263)	*0.520*
**36**	679	(306)	*0.445*	606	(252)	*0.428*	601	(250)	*0.420*	638	(275)	*0.421*
**38**	735	(645)	*0.006*	721	(586)	*0.013*	687	(561)	*0.068*	715	(606)	*0.031*
**5L**	403	(123)	*0.306*	335	(89)	*0.266*	313	(97)	*0.309*	366	(115)	*0.314*

**5M**	413	(90)	*0.218*	337	(99)	*0.295*	338	(107)	*0.316*	382	(99)	*0.259*
**PGa**	414	(74)	*0.178*	336	(62)	*0.183*	334	(64)	*0.191*	377	(62)	*0.166*
**PGp**	451	(182)	*0.176*	391	(166)	*0.193*	384	(165)	*0.175*	421	(176)	*0.179*
**PFt**	531	(177)	*0.279*	448	(138)	*0.257*	438	(130)	*0.200*	485	(157)	*0.241*
**PFm**	561	(148)	*0.265*	469	(96)	*0.205*	457	(120)	*0.263*	513	(134)	*0.261*
**24**	546	(40)	*0.074*	--	--	--	467	(16)	*0.035*	509	(24)	*0.046*
**32**	410	(50)	*0.122*	372	(40)	*0.106*	346	(19)	*0.056*	383	(36)	*0.094*
**23**	407	(141)	*0.347*	318	(101)	*0.316*	300	(68)	*0.226*	364	(114)	*0.313*
**31**	439	(140)	*0.320*	361	(141)	*0.391*	354	(133)	*0.377*	400	(139)	*0.346*
**4**	404	(73)	*0.361*	272	(30)	*0.226*	224	(9)	*0.446*	286	(40)	*0.393*
**6**	336	(82)	*0.189*	290	(99)	*0.280*	272	(106)	*0.246*	301	(97)	*0.214*
**8**	690	(431)	*0.399*	649	(480)	*0.412*	603	(456)	*0.433*	654	(444)	*0.405*
**9**	675	(151)	*0.220*	631	(245)	*0.200*	608	(204)	*0.179*	647	(176)	*0.203*
**10L**	695	(486)	*0.276*	636	(474)	*0.274*	623	(499)	*0.335*	663	(490)	*0.291*
**10M**	714	(565)	*0.411*	710	(596)	*0.370*	715	(640)	*0.426*	714	(596)	*0.406*
**11**	811	(42)	*0.212*	754	(43)	*0.208*	764	(49)	*0.166*	791	(44)	*0.204*
**44**	602	(128)	*0.229*	487	(51)	*0.031*	468	(25)	*0.015*	492	(70)	*0.151*
**45**	524	(419)	*0.018*	415	(324)	*0.069*	393	(305)	*0.011*	472	(379)	*0.028*
**46**	693	(188)	*0.271*	595	(204)	*0.342*	585	(202)	*0.346*	644	(195)	*0.302*
**47**	718	(345)	*0.257*	640	(320)	*0.193*	651	(334)	*0.186*	689	(335)	*0.233*

						NMDA						
**Region**	**supragranular**	**(SD)**	***CV***	**granular**	**(SD)**	***CV***	**infragranular**	**(SD)**	***CV***	**all layers**	**(SD)**	***CV***
**1**	1369	(262)	*0.192*	1003	(208)	*0.207*	862	(190)	*0.221*	1169	(246)	*0.210*
**2**	1450	(246)	*0.190*	1189	(224)	*0.160*	943	(189)	*0.169*	1271	(225)	*0.188*
**3a**	1352	(303)	*0.195*	1114	(253)	*0.193*	863	(195)	*0.198*	1178	(280)	*0.198*
**3b**	1438	(266)	*0.185*	1182	(210)	*0.178*	878	(132)	*0.151*	1254	(203)	*0.162*
**V1**	1963	(429)	*0.219*	1769	(366)	*0.207*	991	(116)	*0.117*	1735	(394)	*0.238*
**V2d**	1785	(386)	*0.216*	1500	(295)	*0.197*	1061	(183)	*0.172*	1450	(280)	*0.178*
**V2v**	1759	(202)	*0.115*	1523	(243)	*0.160*	1065	(199)	*0.187*	1449	(81)	*0.141*

**V3A**	1759	(350)	0.199	1451	(192)	0.132	1215	(232)	0.191	1526	(267)	0.227
**V3d**	1599	(519)	0.325	1247	(339)	0.272	979	(249)	0.254	1361	(411)	0.216
**V3v**	1784	(318)	0.178	1607	(329)	0.205	1111	(251)	0.226	1496	(215)	0.178
**V4v**	1545	(303)	0.196	1479	(391)	0.264	945	(292)	0.309	1248	(328)	0.157
**FG1**	1503	(381)	0.254	1337	(638)	0.477	1019	(636)	0.624	1279	(509)	0.398
**FG2**	1699	(463)	0.272	1425	(717)	0.503	1089	(593)	0.545	1382	(586)	0.424
**37B**	1423	(406)	0.285	1235	(413)	0.334	1009	(326)	0.323	1251	(382)	0.306
**37L**	1500	(430)	0.287	1258	(356)	0.283	1048	(310)	0.296	1312	(382)	0.291
**37M**	1589	(556)	0.274	1398	(503)	0.268	1110	(410)	0.240	1379	(514)	0.268
**41**	1498	(361)	0.241	1145	(325)	0.284	867	(234)	0.270	1266	(314)	0.248
**42**	1506	(287)	0.190	1136	(237)	0.209	873	(202)	0.232	1272	(266)	0.209
**20**	1387	(329)	0.122	1269	(329)	0.149	1089	(352)	0.236	1257	(322)	0.146
**21**	1453	(575)	0.290	1272	(521)	0.287	1128	(464)	0.280	1314	(525)	0.289
**22**	1408	(338)	0.240	1126	(267)	0.237	935	(230)	0.246	1212	(287)	0.237
**36**	1460	(394)	0.269	1374	(400)	0.291	1187	(336)	0.283	1343	(369)	0.275
**38**	1591	(325)	0.204	1429	(114)	0.079	1225	(43)	0.035	1432	(169)	0.118
**5L**	1244	(451)	0.070	1054	(360)	0.089	851	(224)	0.134	1095	(360)	0.055
**5M**	1113	(198)	0.178	948	(172)	0.181	811	(166)	0.204	1000	(181)	0.181
**PGa**	1341	(415)	0.310	1129	(396)	0.350	960	(346)	0.360	1187	(395)	0.333
**PGp**	1372	(361)	0.263	1176	(287)	0.244	969	(190)	0.196	1214	(306)	0.253
**PFt**	1327	(263)	0.198	1121	(312)	0.279	968	(307)	0.318	1162	(283)	0.243
**PFm**	1434	(340)	0.237	1188	(343)	0.289	982	(292)	0.298	1244	(311)	0.250
**24**	1622	(1369)	0.046	--	--	--	1329	(1117)	0.035	1478	(1241)	0.025
**32**	1421	(1277)	0.074	1352	(1192)	0.036	1122	(928)	0.050	1299	(1126)	0.070
**23**	1302	(380)	0.292	1068	(389)	0.364	875	(331)	0.378	1137	(334)	0.294
**31**	1366	(336)	0.246	1234	(349)	0.282	1037	(295)	0.284	1234	(316)	0.256
**4**	947	(8)	0.119	776	(53)	0.083	617	(17)	0.109	739	(1)	0.128
**6**	873	(104)	0.344	789	(197)	0.053	633	(153)	0.330	751	(118)	0.351
**8**	1421	(1362)	0.114	1227	(1144)	0.164	1054	(938)	0.148	1255	(1159)	0.125
**9**	1272	(1261)	0.163	1090	(1017)	0.175	933	(834)	0.185	1123	(1067)	0.174

**10L**	1102	(1052)	*0.116*	966	(868)	*0.066*	861	(753)	*0.032*	1006	(934)	*0.085*
**10M**	1187	(1232)	*0.001*	1077	(1036)	*0.041*	934	(886)	*0.021*	1079	(1076)	*0.008*
**11**	1341	(1372)	*0.001*	1235	(1231)	*0.003*	1067	(1036)	*0.035*	1234	(1236)	*0.010*
**44**	1097	(169)	*0.034*	958	(62)	*0.073*	856	(55)	*0.077*	964	(95)	*0.050*
**45**	1191	(37)	*0.031*	1092	(28)	*0.025*	901	(18)	*0.021*	1078	(40)	*0.038*
**46**	1211	(1107)	*0.183*	1014	(874)	*0.141*	931	(763)	*0.167*	1091	(959)	*0.174*
**47**	1115	(913)	*0.170*	999	(786)	*0.124*	901	(691)	*0.118*	1033	(818)	*0.149*

						**kainate**						
**Region**	**supragranular**	**(SD)**	***CV***	**granular**	**(SD)**	***CV***	**infragranular**	**(SD)**	***CV***	**all layers**	**(SD)**	***CV***
**1**	599	(309)	*0.097*	608	(342)	*0.048*	649	(375)	*0.036*	615	(330)	*0.059*
**2**	591	(282)	*0.307*	640	(342)	*0.394*	669	(386)	*0.338*	625	(323)	*0.313*
**3a**	598	(356)	*0.445*	598	(410)	*0.517*	590	(416)	*0.577*	593	(378)	*0.496*
**3b**	526	(286)	*0.378*	529	(332)	*0.536*	644	(395)	*0.507*	560	(325)	*0.439*
**V1**	297	(333)	*1.124*	417	(477)	*1.144*	304	(335)	*1.102*	329	(367)	*0.889*
**V2d**	310	(344)	*1.110*	359	(416)	*1.157*	320	(335)	*1.048*	318	(346)	*0.860*
**V2v**	295	(321)	*1.091*	305	(348)	*1.142*	422	(509)	*1.204*	366	(425)	*0.907*
**V3A**	354	(399)	*1.128*	408	(488)	*1.197*	397	(453)	*1.140*	376	(429)	*0.923*
**V3d**	370	(431)	*1.164*	492	(598)	*1.216*	340	(373)	*1.094*	369	(424)	*0.914*
**V3v**	323	(351)	*1.088*	344	(384)	*1.117*	461	(547)	*1.188*	391	(448)	*0.920*
**V4v**	308	(335)	*1.086*	352	(358)	*1.018*	417	(452)	*1.083*	362	(390)	*0.848*
**FG1**	413	(230)	*0.556*	429	(216)	*0.504*	524	(369)	*0.704*	462	(90)	*0.626*
**FG2**	316	(117)	*0.370*	324	(189)	*0.582*	362	(200)	*0.553*	335	(159)	*0.473*
**37B**	454	(279)	*0.300*	581	(429)	*0.332*	594	(363)	*0.265*	519	(330)	*0.289*
**37L**	546	(314)	*0.169*	612	(387)	*0.105*	642	(337)	*0.050*	588	(323)	*0.102*
**37M**	530	(307)	*0.150*	582	(300)	*0.036*	642	(355)	*0.076*	577	(320)	*0.082*
**41**	532	(324)	*0.608*	577	(344)	*0.596*	657	(425)	*0.646*	573	(351)	*0.613*
**42**	583	(291)	*0.415*	626	(374)	*0.500*	666	(387)	*0.414*	615	(327)	*0.420*
**20**	555	(381)	*0.665*	730	(532)	*0.604*	829	(588)	*0.513*	666	(453)	*0.589*
**21**	665	(366)	*0.327*	794	(484)	*0.287*	876	(517)	*0.296*	762	(438)	*0.314*

**22**	623	(352)	*0.244*	714	(408)	*0.186*	745	(439)	*0.151*	675	(386)	*0.198*
**36**	568	(371)	*0.652*	700	(510)	*0.729*	810	(558)	*0.688*	673	(456)	*0.677*
**38**	402	(238)	*0.148*	493	(289)	*0.018*	626	(360)	*0.071*	496	(294)	*0.092*
**5L**	422	(289)	*0.477*	448	(349)	*0.410*	476	(354)	*0.402*	441	(315)	*0.440*
**5M**	368	(186)	*0.230*	409	(285)	*0.455*	417	(337)	*0.562*	388	(245)	*0.370*
**PGa**	490	(302)	*0.138*	577	(360)	*0.096*	645	(387)	*0.087*	553	(334)	*0.111*
**PGp**	477	(374)	*0.558*	564	(451)	*0.509*	570	(420)	*0.509*	519	(397)	*0.531*
**PFt**	414	(192)	*0.424*	509	(255)	*0.401*	532	(249)	*0.318*	469	(217)	*0.379*
**PFm**	484	(337)	*0.336*	559	(394)	*0.287*	585	(415)	*0.266*	532	(373)	*0.283*
**24**	753	(398)	*0.046*	--	--	--	914	(466)	*0.083*	829	(434)	*0.050*
**32**	615	(419)	*0.300*	716	(461)	*0.172*	767	(427)	*0.173*	681	(430)	*0.218*
**23**	504	(227)	*0.451*	530	(227)	*0.429*	606	(320)	*0.528*	541	(260)	*0.481*
**31**	522	(244)	*0.151*	587	(309)	*0.144*	681	(362)	*0.118*	587	(301)	*0.128*
**4**	346	(50)	*0.088*	283	(25)	*0.149*	257	(20)	*0.135*	286	(35)	*0.102*
**6**	280	(24)	*0.143*	296	(17)	*0.076*	330	(21)	*0.184*	305	(21)	*0.115*
**8**	593	(461)	*0.259*	705	(509)	*0.240*	780	(496)	*0.217*	677	(489)	*0.225*
**9**	634	(359)	*0.038*	749	(418)	*0.159*	899	(443)	*0.060*	759	(415)	*0.044*
**10L**	562	(303)	*0.162*	727	(339)	*0.227*	828	(398)	*0.166*	673	(336)	*0.180*
**10M**	582	(338)	*0.004*	726	(363)	*0.045*	844	(426)	*0.032*	699	(381)	*0.029*
**11**	654	(386)	*0.009*	838	(475)	*0.049*	923	(503)	*0.035*	766	(444)	*0.018*
**44**	459	(112)	*0.191*	521	(91)	*0.091*	601	(78)	*0.143*	498	(84)	*0.163*
**45**	397	(275)	*0.173*	442	(225)	*0.134*	490	(268)	*0.098*	434	(264)	*0.117*
**46**	592	(316)	*0.125*	716	(336)	*0.076*	800	(333)	*0.066*	676	(327)	*0.087*
**47**	501	(239)	*0.126*	627	(277)	*0.090*	733	(282)	*0.062*	586	(267)	*0.089*

						**GABAA**						
**Region**	**supragranular**	**(SD)**	***CV***	**granular**	**(SD)**	***CV***	**infragranular**	**(SD)**	***CV***	**all layers**	**(SD)**	***CV***
**1**	2053	(403)	*0.196*	1296	(319)	*0.246*	791	(219)	*0.277*	1545	(336)	*0.217*
**2**	2050	(408)	*0.199*	1594	(415)	*0.261*	985	(304)	*0.308*	1687	(344)	*0.204*
**3a**	2071	(476)	*0.230*	1456	(408)	*0.280*	836	(269)	*0.322*	1630	(413)	*0.253*

**3b**	2294	(434)	*0.189*	1678	(368)	*0.219*	864	(232)	*0.268*	1849	(378)	*0.204*
**V1**	3201	(1494)	*0.470*	2941	(999)	*0.341*	794	(100)	*0.127*	2702	(1178)	*0.319*
**V2d**	2719	(1181)	*0.434*	1693	(36)	*0.021*	846	(116)	*0.137*	1826	(502)	*0.216*
**V2v**	3178	(992)	*0.310*	2819	(665)	*0.236*	1420	(54)	*0.038*	2342	(255)	*0.226*
**V3A**	2716	(876)	*0.323*	2050	(611)	*0.298*	1063	(217)	*0.204*	2016	(522)	*0.291*
**V3d**	2759	(1025)	*0.371*	1983	(333)	*0.168*	947	(66)	*0.070*	2091	(629)	*0.258*
**V3v**	3154	(1006)	*0.319*	2541	(825)	*0.325*	1319	(484)	*0.367*	2338	(570)	*0.268*
**V4v**	2768	(829)	*0.299*	2931	(1584)	*0.539*	1509	(959)	*0.636*	2167	(998)	*0.350*
**FG1**	1637	(85)	*0.052*	1278	(341)	*0.267*	831	(454)	*0.546*	1260	(263)	*0.208*
**FG2**	1788	(757)	*0.424*	1612	(1080)	*0.670*	938	(736)	*0.784*	1368	(830)	*0.606*
**37B**	1928	(216)	*0.112*	1791	(274)	*0.153*	1163	(170)	*0.146*	1632	(204)	*0.125*
**37L**	1881	(588)	*0.312*	1486	(420)	*0.283*	927	(321)	*0.346*	1509	(483)	*0.320*
**37M**	1811	(447)	*0.247*	1573	(497)	*0.316*	912	(402)	*0.441*	1433	(466)	*0.325*
**41**	2205	(609)	*0.276*	1651	(687)	*0.416*	867	(335)	*0.387*	1724	(520)	*0.302*
**42**	1904	(525)	*0.276*	1462	(454)	*0.310*	959	(149)	*0.155*	1544	(387)	*0.251*
**20**	1684	(490)	*0.291*	1614	(486)	*0.301*	1076	(264)	*0.245*	1437	(376)	*0.262*
**21**	1925	(468)	*0.243*	1803	(469)	*0.260*	1208	(295)	*0.244*	1636	(381)	*0.233*
**22**	1890	(535)	*0.283*	1349	(310)	*0.230*	814	(213)	*0.262*	1452	(343)	*0.236*
**36**	1623	(579)	*0.356*	1534	(426)	*0.278*	1018	(175)	*0.171*	1366	(377)	*0.276*
**38**	1613	(621)	*0.385*	1533	(475)	*0.310*	1089	(352)	*0.324*	1406	(495)	*0.352*
**5L**	1952	(758)	*0.388*	1533	(685)	*0.447*	974	(498)	*0.511*	1626	(662)	*0.407*
**5M**	1844	(727)	*0.394*	1354	(645)	*0.476*	892	(413)	*0.462*	1463	(555)	*0.380*
**PGa**	2030	(607)	*0.299*	1684	(525)	*0.312*	1114	(410)	*0.368*	1672	(524)	*0.313*
**PGp**	2253	(573)	*0.254*	1864	(483)	*0.259*	1218	(375)	*0.308*	1848	(482)	*0.261*
**PFt**	1787	(512)	*0.286*	1389	(365)	*0.263*	862	(167)	*0.193*	1369	(285)	*0.208*
**PFm**	2112	(270)	*0.128*	1638	(358)	*0.218*	992	(253)	*0.255*	1649	(229)	*0.139*
**24**	1403	(1095)	*0.264*	--	--	--	992	(842)	*0.311*	1197	(955)	*0.253*
**32**	1440	(1120)	*0.778*	1184	(815)	*0.688*	781	(527)	*0.676*	1164	(838)	*0.720*
**23**	2187	(551)	*0.252*	1472	(427)	*0.290*	836	(257)	*0.308*	1671	(402)	*0.241*
**31**	2087	(530)	*0.254*	1805	(524)	*0.291*	1253	(413)	*0.330*	1787	(467)	*0.262*

**4**	1643	(90)	*0.293*	1032	(13)	*0.140*	639	(45)	*0.558*	999	(108)	*0.335*
**6**	1275	(133)	*0.381*	989	(291)	*0.560*	657	(150)	*0.537*	953	(116)	*0.431*
**8**	1577	(1198)	*0.331*	1257	(818)	*0.239*	816	(631)	*0.250*	1248	(914)	*0.295*
**9**	1487	(844)	*0.181*	1274	(618)	*0.241*	923	(545)	*0.312*	1272	(724)	*0.217*
**10L**	1542	(956)	*0.003*	1362	(836)	*0.005*	977	(750)	*0.032*	1330	(871)	*0.007*
**10M**	1555	(982)	*0.108*	1481	(904)	*0.165*	1036	(769)	*0.199*	1357	(885)	*0.111*
**11**	1666	(1146)	*0.116*	1496	(915)	*0.156*	1074	(716)	*0.249*	1452	(975)	*0.142*
**44**	1833	(240)	*0.142*	1513	(61)	*0.096*	1099	(30)	*0.109*	1302	(91)	*0.113*
**45**	1772	(345)	*0.194*	1479	(220)	*0.149*	1058	(163)	*0.154*	1515	(283)	*0.187*
**46**	1690	(924)	*0.547*	1440	(772)	*0.536*	1012	(548)	*0.541*	1423	(762)	*0.535*
**47**	1745	(1088)	*0.624*	1523	(924)	*0.606*	1132	(736)	*0.650*	1535	(920)	*0.600*

						**GABAA/BZ**						
**Region**	**supragranular**	**(SD)**	***CV***	**granular**	**(SD)**	***CV***	**infragranular**	**(SD)**	***CV***	**all layers**	**(SD)**	***CV***
**1**	3034	(1163)	*0.288*	2296	(1089)	*0.323*	1710	(800)	*0.365*	2507	(1058)	*0.292*
**2**	3105	(1250)	*0.239*	2758	(1394)	*0.177*	1951	(963)	*0.243*	2685	(1131)	*0.198*
**3a**	2953	(1294)	*0.438*	2307	(1187)	*0.514*	1490	(805)	*0.540*	2405	(1095)	*0.455*
**3b**	3272	(1039)	*0.147*	2801	(1049)	*0.147*	1757	(575)	*0.122*	2826	(888)	*0.133*
**V1**	4006	(3477)	*0.868*	3842	(3219)	*0.838*	1439	(1066)	*0.741*	3525	(3035)	*0.562*
**V2d**	4213	(3783)	*0.898*	3477	(2973)	*0.855*	2099	(1908)	*0.909*	3234	(2879)	*0.579*
**V2v**	4164	(3435)	*0.825*	3584	(2882)	*0.804*	2265	(1746)	*0.771*	3174	(2349)	*0.526*
**V3A**	3061	(2240)	*0.732*	2381	(1616)	*0.679*	1636	(1392)	*0.851*	2446	(1796)	*0.515*
**V3d**	3098	(2325)	*0.750*	2395	(1535)	*0.641*	1766	(1265)	*0.716*	2594	(1894)	*0.479*
**V3v**	3907	(3126)	*0.800*	3162	(2364)	*0.747*	1840	(1421)	*0.773*	2917	(2139)	*0.513*
**V4v**	3204	(2070)	*0.646*	2830	(1997)	*0.706*	1541	(1347)	*0.874*	2380	(1774)	*0.526*
**FG1**	2427	(488)	*0.325*	1962	(538)	*0.274*	1379	(192)	*0.139*	1938	(512)	*0.264*
**FG2**	2745	(346)	*0.126*	2534	(160)	*0.063*	1759	(169)	*0.096*	2238	(6)	*0.003*
**37B**	3282	(1748)	*0.533*	3181	(1867)	*0.587*	2221	(1107)	*0.499*	2850	(1510)	*0.530*
**37L**	3640	(1182)	*0.325*	3126	(1266)	*0.405*	2257	(669)	*0.297*	3118	(1025)	*0.329*
**37M**	3639	(1247)	*0.199*	3524	(1257)	*0.066*	2481	(796)	*0.153*	3181	(1089)	*0.167*

**41**	3130	(993)	*0.130*	2645	(1114)	*0.122*	1642	(576)	*0.201*	2630	(930)	*0.125*
**42**	3206	(1019)	*0.118*	2552	(654)	*0.118*	1763	(346)	*0.185*	2674	(816)	*0.141*
**20**	2741	(1438)	*0.301*	2799	(1408)	*0.229*	2113	(846)	*0.277*	2512	(1187)	*0.269*
**21**	2837	(1365)	*0.196*	2759	(1379)	*0.131*	2080	(846)	*0.171*	2552	(1218)	*0.168*
**22**	2966	(1222)	*0.248*	2591	(1185)	*0.215*	1808	(670)	*0.184*	2484	(982)	*0.231*
**36**	2514	(1541)	*0.311*	2555	(1572)	*0.237*	2013	(1171)	*0.335*	2322	(1393)	*0.302*
**38**	2130	(1442)	*0.129*	2072	(1249)	*0.149*	1696	(938)	*0.048*	1961	(1233)	*0.100*
**5L**	3718	(1185)	*0.319*	3329	(921)	*0.277*	2531	(606)	*0.239*	3319	(1020)	*0.307*
**5M**	3837	(1015)	*0.264*	3265	(1232)	*0.377*	2463	(882)	*0.358*	3321	(977)	*0.294*
**PGa**	3824	(1177)	*0.398*	3349	(971)	*0.328*	2594	(677)	*0.351*	3349	(996)	*0.376*
**PGp**	3568	(1272)	*0.256*	3182	(1149)	*0.148*	2319	(714)	*0.198*	3098	(1079)	*0.249*
**PFt**	3708	(865)	*0.221*	3275	(953)	*0.187*	2500	(764)	*0.272*	3194	(882)	*0.254*
**PFm**	3940	(1750)	*0.591*	3404	(1716)	*0.463*	2598	(1125)	*0.498*	3407	(1481)	*0.557*
**24**	3040	(1494)	*0.156*	--	--	--	2524	(1150)	*0.120*	2797	(1322)	*0.152*
**32**	3078	(1562)	*0.175*	2902	(1336)	*0.126*	2257	(903)	*0.097*	2767	(1287)	*0.155*
**23**	3480	(1137)	*0.218*	3102	(1249)	*0.257*	2263	(1036)	*0.290*	3023	(1092)	*0.239*
**31**	3246	(1137)	*0.280*	3154	(1341)	*0.439*	2547	(1158)	*0.548*	3004	(1093)	*0.358*
**4**	2638	(64)	*0.242*	1921	(133)	*0.352*	1374	(102)	*0.338*	1842	(10)	*0.234*
**6**	2187	(177)	*0.139*	1925	(419)	*0.631*	1462	(235)	*0.232*	1811	(181)	*0.147*
**8**	2531	(833)	*0.055*	2289	(533)	*0.037*	1901	(490)	*0.037*	2255	(638)	*0.039*
**9**	2808	(1091)	*0.265*	2668	(728)	*0.185*	2254	(534)	*0.132*	2577	(825)	*0.213*
**10L**	2637	(962)	*0.062*	2601	(733)	*0.092*	2170	(645)	*0.082*	2474	(840)	*0.073*
**10M**	3107	(752)	*0.132*	2975	(677)	*0.152*	2383	(557)	*0.185*	2820	(658)	*0.146*
**11**	3034	(992)	*0.014*	3003	(858)	*0.015*	2546	(682)	*0.046*	2869	(869)	*0.005*
**44**	2254	(118)	*0.059*	2003	(64)	*0.104*	1651	(66)	*0.082*	1797	(105)	*0.072*
**45**	2393	(1780)	*0.028*	1862	(1089)	*0.082*	1433	(635)	*0.062*	2038	(1372)	*0.058*
**46**	3089	(1569)	*0.040*	2735	(1168)	*0.040*	2274	(905)	*0.015*	2774	(1297)	*0.042*
**47**	2892	(1313)	*0.068*	2654	(1091)	*0.005*	2214	(858)	*0.009*	2658	(1130)	*0.041*

GABAB

Region	supragranular	(SD)	CV	granular	(SD)	CV	infragranular	(SD)	CV	all layers	(SD)	CV
**1**	2534	(564)	*0.095*	1762	(463)	*0.212*	1546	(450)	*0.239*	2108	(539)	*0.138*
**2**	2895	(876)	*0.303*	2245	(873)	*0.389*	1897	(737)	*0.388*	2495	(808)	*0.324*
**3a**	2549	(676)	*0.265*	1761	(482)	*0.274*	1345	(367)	*0.273*	2075	(580)	*0.279*
**3b**	2719	(680)	*0.250*	1718	(450)	*0.262*	1497	(406)	*0.271*	2256	(539)	*0.239*
**V1**	2664	(682)	*0.256*	1866	(477)	*0.256*	1137	(96)	*0.085*	2165	(609)	*0.159*
**V2d**	2543	(824)	*0.324*	1869	(592)	*0.317*	1328	(499)	*0.376*	1964	(649)	*0.197*
**V2v**	2269	(101)	*0.000*	1932	(265)	*0.137*	1461	(195)	*0.133*	1920	(40)	*0.071*
**V3A**	2108	(159)	*0.001*	1509	(73)	*0.048*	1151	(21)	*0.019*	1692	(26)	*0.130*
**V3d**	2008	(83)	*0.041*	1474	(205)	*0.139*	982	(165)	*0.168*	1617	(119)	*0.150*
**V3v**	2662	(282)	*0.106*	2014	(164)	*0.081*	1454	(212)	*0.146*	2119	(103)	*0.049*
**V4v**	2125	(393)	*0.185*	1988	(313)	*0.157*	1304	(293)	*0.225*	1715	(371)	*0.164*
**FG1**	2562	(270)	*0.105*	1980	(105)	*0.053*	1499	(110)	*0.073*	2058	(92)	*0.045*
**FG2**	2960	(54)	*0.018*	2325	(275)	*0.118*	1857	(343)	*0.185*	2371	(237)	*0.100*
**37B**	2399	(1079)	*0.450*	2046	(955)	*0.467*	1731	(849)	*0.491*	2105	(988)	*0.470*
**37L**	2713	(1146)	*0.422*	2120	(824)	*0.389*	1858	(744)	*0.400*	2354	(993)	*0.422*
**37M**	2648	(1077)	*0.208*	2189	(890)	*0.230*	1852	(733)	*0.223*	2274	(927)	*0.228*
**41**	2563	(598)	*0.234*	1718	(405)	*0.236*	1477	(384)	*0.260*	2131	(537)	*0.252*
**42**	2982	(890)	*0.298*	2112	(574)	*0.272*	1728	(442)	*0.256*	2453	(652)	*0.266*
**20**	2995	(953)	*0.228*	2678	(957)	*0.272*	2386	(851)	*0.274*	2729	(910)	*0.246*
**21**	3047	(1043)	*0.317*	2647	(1052)	*0.312*	2309	(933)	*0.298*	2721	(996)	*0.325*
**22**	2967	(1104)	*0.359*	2351	(1011)	*0.466*	1850	(726)	*0.421*	2422	(827)	*0.325*
**36**	3089	(1265)	*0.326*	2716	(1080)	*0.401*	2467	(971)	*0.411*	2801	(1121)	*0.358*
**38**	1880	(1432)	*0.081*	1679	(1193)	*0.091*	1489	(1024)	*0.142*	1706	(1244)	*0.101*
**5L**	2881	(664)	*0.141*	2356	(636)	*0.244*	1952	(517)	*0.190*	2527	(577)	*0.151*
**5M**	2800	(591)	*0.211*	2144	(647)	*0.302*	2001	(638)	*0.319*	2450	(574)	*0.234*
**PGa**	2168	(784)	*0.362*	1712	(669)	*0.391*	1489	(580)	*0.389*	1889	(712)	*0.377*
**PGp**	2654	(903)	*0.201*	2096	(692)	*0.166*	1819	(616)	*0.187*	2303	(793)	*0.199*
**PFt**	2582	(1091)	*0.297*	2080	(853)	*0.297*	1800	(794)	*0.277*	2222	(948)	*0.280*
**PFm**	2199	(1053)	*0.406*	1699	(852)	*0.388*	1487	(759)	*0.408*	1881	(914)	*0.417*

**24**	3018	(675)	*0.224*	--	--	--	2340	(695)	*0.297*	2702	(684)	*0.253*
**32**	2772	(863)	*0.024*	2358	(851)	*0.016*	2060	(776)	*0.007*	2481	(800)	*0.017*
**23**	2293	(628)	*0.274*	1764	(525)	*0.297*	1560	(524)	*0.336*	1988	(569)	*0.286*
**31**	2375	(569)	*0.240*	1955	(539)	*0.275*	1747	(552)	*0.316*	2115	(537)	*0.254*
**4**	1901	(6)	*0.202*	1219	(132)	*0.095*	996	(166)	*0.237*	1337	(65)	*0.196*
**6**	2004	(71)	*0.044*	1596	(83)	*0.243*	1379	(32)	*0.081*	1675	(40)	*0.026*
**8**	2995	(1575)	*0.094*	2425	(1146)	*0.096*	2155	(1040)	*0.069*	2605	(1283)	*0.089*
**9**	3224	(1302)	*0.053*	2691	(1017)	*0.057*	2428	(953)	*0.066*	2869	(1129)	*0.053*
**10L**	2740	(1020)	*0.011*	2450	(859)	*0.007*	2167	(769)	*0.015*	2503	(927)	*0.001*
**10M**	2808	(1069)	*0.026*	2521	(974)	*0.040*	2224	(836)	*0.002*	2552	(956)	*0.028*
**11**	3227	(1502)	*0.032*	2917	(1372)	*0.009*	2610	(1246)	*0.020*	2985	(1394)	*0.007*
**44**	1862	(87)	*0.079*	1494	(5)	*0.108*	1353	(44)	*0.024*	1487	(64)	*0.067*
**45**	1647	(1391)	*0.017*	1225	(950)	*0.010*	1083	(866)	*0.011*	1428	(1197)	*0.032*
**46**	2878	(617)	*0.076*	2396	(582)	*0.083*	2154	(528)	*0.080*	2584	(593)	*0.082*
**47**	2975	(1004)	*0.004*	2582	(936)	*0.056*	2322	(867)	*0.081*	2723	(923)	*0.027*

M_1

**Region**	**supragranular**	**(SD)**	***CV***	**granular**	**(SD)**	***CV***	**infragranular**	**(SD)**	***CV***	**all layers**	**(SD)**	***CV***
**1**	745	(270)	*0.517*	493	(121)	*0.498*	387	(50)	*0.428*	597	(190)	*0.506*
**2**	815	(248)	*0.430*	584	(195)	*0.515*	481	(172)	*0.538*	682	(208)	*0.453*
**3a**	656	(130)	*0.349*	435	(41)	*0.386*	315	(2)	*0.314*	535	(112)	*0.390*
**3b**	771	(165)	*0.341*	482	(85)	*0.320*	372	(61)	*0.244*	631	(122)	*0.324*
**V1**	730	(127)	*0.174*	562	(15)	*0.026*	278	(71)	*0.256*	594	(60)	*0.184*
**V2d**	660	(88)	*0.134*	476	(32)	*0.067*	294	(100)	*0.340*	487	(93)	*0.138*
**V2v**	731	(122)	*0.166*	693	(105)	*0.151*	412	(33)	*0.080*	606	(98)	*0.152*
**V3A**	460	(73)	*0.160*	352	(79)	*0.223*	264	(44)	*0.167*	377	(72)	*0.227*
**V3d**	478	(69)	*0.145*	351	(77)	*0.218*	223	(101)	*0.454*	381	(81)	*0.244*
**V3v**	803	(181)	*0.225*	617	(110)	*0.178*	313	(52)	*0.166*	578	(25)	*0.196*
**V4v**	766	(297)	*0.388*	645	(271)	*0.419*	420	(240)	*0.571*	590	(280)	*0.360*
**FG1**	721	(480)	*0.874*	600	(314)	*0.774*	420	(139)	*0.689*	580	(320)	*0.809*

**FG2**	739	(511)	*0.894*	567	(262)	*0.726*	427	(176)	*0.764*	564	(311)	*0.815*
**37B**	912	(79)	*0.251*	746	(46)	*0.263*	613	(24)	*0.206*	775	(31)	*0.234*
**37L**	1090	(157)	*0.281*	899	(48)	*0.220*	772	(195)	*0.192*	952	(98)	*0.261*
**37M**	783	(297)	*0.571*	613	(152)	*0.493*	528	(122)	*0.515*	655	(214)	*0.555*
**41**	871	(223)	*0.422*	576	(120)	*0.387*	435	(51)	*0.273*	699	(163)	*0.428*
**42**	899	(209)	*0.399*	647	(144)	*0.455*	505	(96)	*0.488*	749	(158)	*0.412*
**20**	728	(277)	*0.516*	622	(215)	*0.608*	589	(205)	*0.668*	662	(240)	*0.570*
**21**	927	(168)	*0.319*	805	(125)	*0.352*	710	(102)	*0.395*	826	(130)	*0.341*
**22**	959	(133)	*0.295*	711	(64)	*0.302*	566	(35)	*0.327*	806	(92)	*0.300*
**36**	800	(156)	*0.383*	703	(119)	*0.383*	629	(110)	*0.414*	718	(120)	*0.377*
**38**	852	(47)	*0.155*	813	(710)	*0.072*	768	(47)	*0.100*	814	(64)	*0.115*
**5L**	593	(228)	*0.663*	468	(146)	*0.544*	397	(83)	*0.584*	514	(167)	*0.622*
**5M**	646	(146)	*0.457*	455	(87)	*0.521*	388	(70)	*0.567*	541	(119)	*0.497*
**PGa**	738	(136)	*0.190*	608	(87)	*0.179*	523	(40)	*0.137*	649	(95)	*0.171*
**PGp**	716	(147)	*0.213*	613	(138)	*0.333*	482	(98)	*0.398*	621	(118)	*0.262*
**PFt**	914	(131)	*0.175*	728	(105)	*0.199*	596	(49)	*0.177*	765	(86)	*0.190*
**PFm**	854	(111)	*0.305*	677	(41)	*0.282*	575	(141)	*0.246*	733	(73)	*0.303*
**24**	820	(224)	*0.052*	--	--	--	680	(293)	*0.015*	755	(153)	*0.060*
**32**	772	(226)	*0.051*	677	(238)	*0.068*	556	(160)	*0.130*	682	(191)	*0.021*
**23**	731	(203)	*0.260*	541	(88)	*0.198*	431	(38)	*0.208*	619	(146)	*0.255*
**31**	804	(242)	*0.487*	664	(216)	*0.551*	570	(171)	*0.563*	712	(215)	*0.513*
**4**	358	(34)	*0.549*	246	(4)	*0.056*	183	(3)	*0.550*	245	(23)	*0.555*
**6**	508	(76)	*0.271*	385	(44)	*0.053*	305	(60)	*0.240*	400	(74)	*0.265*
**8**	685	(211)	*0.042*	585	(174)	*0.066*	519	(137)	*0.015*	609	(170)	*0.020*
**9**	653	(292)	*0.237*	576	(105)	*0.284*	543	(268)	*0.195*	606	(283)	*0.228*
**10L**	600	(250)	*0.025*	568	(231)	*0.086*	483	(223)	*0.153*	556	(240)	*0.061*
**10M**	570	(64)	*0.189*	548	(69)	*0.209*	466	(73)	*0.293*	531	(66)	*0.211*
**11**	606	(102)	*0.172*	617	(66)	*0.131*	529	(39)	*0.190*	581	(76)	*0.172*
**44**	679	(76)	*0.392*	695	(48)	*0.359*	635	(22)	*0.230*	631	(49)	*0.309*
**45**	696	(633)	*0.137*	677	(135)	*0.133*	615	(278)	*0.045*	676	(493)	*0.069*

**46**	749	(397)	*0.261*	672	(174)	*0.239*	583	(194)	*0.188*	684	(362)	*0.236*
**47**	761	(405)	*0.124*	730	(41)	*0.127*	653	(370)	*0.135*	725	(388)	*0.130*

M_2

**Region**	**supragranular**	**(SD)**	***CV***	**granular**	**(SD)**	***CV***	**infragranular**	**(SD)**	***CV***	**all layers**	**(SD)**	***CV***
**1**	222	(34)	*0.154*	204	(47)	*0.230*	153	(26)	*0.172*	199	(32)	*0.161*
**2**	171	(48)	*0.281*	179	(56)	*0.311*	164	(53)	*0.324*	171	(48)	*0.282*
**3a**	191	(47)	*0.246*	187	(48)	*0.258*	165	(57)	*0.346*	183	(50)	*0.274*
**3b**	313	(70)	*0.224*	291	(58)	*0.201*	207	(52)	*0.250*	282	(58)	*0.207*
**V1**	483	(65)	*0.137*	535	(92)	*0.172*	311	(104)	*0.334*	462	(80)	*0.140*
**V2d**	477	(243)	*0.503*	443	(213)	*0.479*	282	(82)	*0.290*	392	(168)	*0.322*
**V2v**	389	(64)	*0.165*	399	(119)	*0.298*	292	(42)	*0.144*	347	(41)	*0.206*
**V3A**	193	(1)	*0.006*	204	(14)	*0.068*	190	(27)	*0.141*	193	(11)	*0.095*
**V3d**	233	(15)	*0.066*	243	(7)	*0.030*	215	(31)	*0.143*	229	(19)	*0.120*
**V3v**	320	(79)	*0.248*	356	(113)	*0.318*	261	(61)	*0.233*	299	(68)	*0.256*
**V4v**	285	(55)	*0.191*	302	(43)	*0.144*	185	(90)	*0.485*	236	(76)	*0.231*
**FG1**	210	(32)	*0.155*	246	(51)	*0.206*	191	(2)	*0.011*	204	(21)	*0.103*
**FG2**	188	(15)	*0.079*	208	(33)	*0.160*	195	(36)	*0.183*	192	(27)	*0.138*
**37B**	135	(58)	*0.429*	154	(60)	*0.393*	146	(51)	*0.351*	141	(54)	*0.386*
**37L**	157	(61)	*0.389*	186	(66)	*0.356*	175	(58)	*0.331*	169	(57)	*0.337*
**37M**	162	(67)	*0.338*	179	(59)	*0.217*	166	(56)	*0.180*	167	(62)	*0.261*
**41**	313	(89)	*0.286*	320	(125)	*0.391*	184	(69)	*0.375*	277	(84)	*0.304*
**42**	193	(55)	*0.284*	218	(66)	*0.301*	158	(58)	*0.366*	187	(54)	*0.290*
**20**	133	(66)	*0.495*	166	(70)	*0.422*	167	(85)	*0.513*	146	(67)	*0.459*
**21**	140	(42)	*0.300*	183	(48)	*0.262*	174	(49)	*0.284*	157	(45)	*0.285*
**22**	187	(43)	*0.229*	191	(56)	*0.293*	140	(71)	*0.505*	171	(49)	*0.285*
**36**	121	(52)	*0.431*	120	(41)	*0.339*	137	(53)	*0.385*	127	(51)	*0.403*
**38**	187	(150)	*0.804*	195	(141)	*0.720*	216	(123)	*0.571*	199	(139)	*0.699*
**5L**	169	(47)	*0.276*	185	(53)	*0.286*	178	(48)	*0.269*	174	(46)	*0.266*
**5M**	199	(49)	*0.246*	209	(55)	*0.262*	186	(41)	*0.222*	197	(46)	*0.232*

**PGa**	219	(47)	*0.214*	259	(41)	*0.158*	225	(42)	*0.185*	227	(34)	*0.151*
**PGp**	229	(72)	*0.314*	256	(58)	*0.225*	205	(55)	*0.266*	226	(59)	*0.263*
**PFt**	168	(43)	*0.255*	199	(39)	*0.196*	180	(32)	*0.179*	177	(35)	*0.197*
**PFm**	187	(40)	*0.213*	208	(35)	*0.170*	179	(40)	*0.222*	187	(36)	*0.192*
**24**	205	(119)	*0.583*	--	--	--	237	(119)	*0.503*	219	(118)	*0.538*
**32**	210	(146)	*0.696*	236	(139)	*0.588*	209	(107)	*0.512*	211	(130)	*0.613*
**23**	208	(51)	*0.247*	219	(45)	*0.207*	163	(42)	*0.258*	196	(45)	*0.231*
**31**	201	(62)	*0.308*	241	(72)	*0.300*	215	(61)	*0.283*	210	(61)	*0.289*
**4**	156	(19)	*0.265*	138	(28)	*0.045*	128	(33)	*0.377*	141	(27)	*0.264*
**6**	150	(54)	*0.407*	157	(45)	*0.538*	139	(32)	*0.575*	145	(44)	*0.455*
**8**	200	(147)	*0.135*	224	(134)	*0.183*	207	(129)	*0.097*	204	(138)	*0.121*
**9**	198	(143)	*0.202*	233	(152)	*0.140*	185	(68)	*0.068*	199	(120)	*0.146*
**10L**	167	(111)	*0.188*	210	(121)	*0.141*	199	(118)	*0.122*	183	(114)	*0.154*
**10M**	176	(140)	*0.214*	217	(156)	*0.176*	217	(147)	*0.094*	195	(144)	*0.148*
**11**	152	(120)	*0.332*	197	(148)	*0.216*	200	(125)	*0.207*	173	(125)	*0.271*
**44**	253	(43)	*0.004*	281	(25)	*0.142*	253	(13)	*0.198*	247	(20)	*0.091*
**45**	211	(171)	*0.204*	241	(157)	*0.197*	214	(147)	*0.278*	215	(162)	*0.222*
**46**	236	(172)	*0.146*	275	(191)	*0.162*	258	(160)	*0.152*	248	(169)	*0.148*
**47**	184	(120)	*0.165*	220	(131)	*0.098*	215	(108)	*0.157*	198	(119)	*0.150*

						**M_3**						
**Region**	**supragranular**	**(SD)**	***CV***	**granular**	**(SD)**	***CV***	**infragranular**	**(SD)**	***CV***	**all layers**	**(SD)**	***CV***
**1**	632	(164)	*0.259*	456	(87)	*0.192*	404	(85)	*0.210*	537	(119)	*0.222*
**2**	671	(275)	*0.410*	545	(213)	*0.392*	468	(194)	*0.415*	597	(247)	*0.414*
**3a**	598	(246)	*0.412*	474	(189)	*0.398*	384	(136)	*0.355*	526	(217)	*0.413*
**3b**	655	(251)	*0.384*	479	(173)	*0.362*	375	(171)	*0.456*	565	(245)	*0.434*
**V1**	639	(39)	*0.051*	498	(21)	*0.009*	387	(29)	*0.040*	540	(53)	*0.018*
**V2d**	566	(43)	*0.136*	415	(43)	*0.135*	249	(58)	*0.123*	421	(40)	*0.104*
**V2v**	560	(54)	*0.025*	449	(39)	*0.003*	310	(39)	*0.041*	478	(38)	*0.023*
**V3A**	437	(38)	*0.084*	395	(15)	*0.098*	284	(46)	*0.093*	382	(20)	*0.083*

**V3d**	467	(29)	*0.016*	389	(32)	*0.019*	285	(21)	*0.013*	401	(37)	*0.049*
**V3v**	569	(52)	*0.013*	482	(17)	*0.025*	329	(15)	*0.013*	485	(53)	*0.061*
**V4v**	435	(91)	*0.082*	417	(72)	*0.011*	295	(13)	*0.032*	361	(24)	*0.120*
**FG1**	651	(53)	*0.296*	543	(81)	*0.323*	457	(30)	*0.236*	561	(105)	*0.206*
**FG2**	675	(80)	*0.035*	591	(90)	*0.014*	459	(63)	*0.016*	575	(84)	*0.026*
**37B**	681	(119)	*0.175*	562	(111)	*0.197*	460	(118)	*0.257*	585	(114)	*0.195*
**37L**	1147	(732)	*0.639*	989	(582)	*0.588*	836	(533)	*0.637*	1014	(651)	*0.642*
**37M**	1134	(718)	*0.633*	960	(548)	*0.571*	791	(428)	*0.541*	963	(563)	*0.584*
**41**	681	(340)	*0.499*	465	(235)	*0.506*	371	(144)	*0.388*	568	(266)	*0.469*
**42**	688	(295)	*0.429*	532	(238)	*0.448*	415	(219)	*0.528*	579	(261)	*0.451*
**20**	672	(463)	*0.581*	604	(393)	*0.534*	538	(359)	*0.501*	610	(418)	*0.541*
**21**	722	(316)	*0.246*	625	(259)	*0.197*	547	(220)	*0.215*	649	(272)	*0.237*
**22**	787	(250)	*0.110*	622	(152)	*0.031*	497	(116)	*0.137*	644	(200)	*0.126*
**36**	683	(480)	*0.570*	593	(392)	*0.547*	517	(370)	*0.489*	604	(414)	*0.545*
**38**	569	(70)	*0.123*	530	(60)	*0.113*	484	(14)	*0.030*	534	(48)	*0.089*
**5L**	1127	(421)	*0.374*	925	(406)	*0.438*	764	(315)	*0.413*	989	(406)	*0.410*
**5M**	1116	(329)	*0.295*	915	(345)	*0.377*	802	(271)	*0.338*	1004	(312)	*0.311*
**PGa**	1232	(340)	*0.276*	1052	(334)	*0.317*	919	(297)	*0.324*	1102	(321)	*0.292*
**PGp**	1738	(161)	*0.017*	1502	(95)	*0.016*	1226	(110)	*0.012*	1500	(121)	*0.016*
**PFt**	852	(166)	*0.109*	718	(166)	*0.260*	616	(157)	*0.201*	746	(164)	*0.141*
**PFm**	847	(131)	*0.154*	632	(136)	*0.215*	556	(151)	*0.271*	720	(139)	*0.193*
**24**	594	(5)	*0.009*	--	--	--	483	(9)	*0.019*	544	(1)	*0.002*
**32**	577	(4)	*0.007*	525	(36)	*0.069*	477	(43)	*0.091*	538	(19)	*0.036*
**23**	735	(289)	*0.393*	559	(206)	*0.368*	478	(200)	*0.418*	643	(258)	*0.402*
**31**	829	(280)	*0.338*	728	(272)	*0.374*	670	(218)	*0.325*	762	(257)	*0.337*
**4**	491	(85)	*0.321*	321	(61)	*0.049*	278	(68)	*0.388*	400	(44)	*0.359*
**6**	478	(13)	*0.020*	419	(32)	*0.254*	354	(21)	*0.153*	412	(7)	*0.036*
**8**	629	(21)	*0.033*	584	(24)	*0.042*	516	(10)	*0.020*	583	(20)	*0.035*
**9**	754	(12)	*0.016*	673	(16)	*0.024*	604	(18)	*0.030*	691	(19)	*0.027*
**10L**	862	(352)	*0.004*	789	(270)	*0.025*	669	(226)	*0.004*	788	(301)	*0.005*

**10M**	810	(283)	*0.018*	812	(280)	*0.056*	728	(251)	*0.082*	779	(266)	*0.022*
**11**	924	(434)	*0.071*	911	(361)	*0.058*	789	(281)	*0.034*	879	(376)	*0.059*
**44**	476	(57)	*0.183*	417	(24)	*0.169*	363	(15)	*0.233*	432	(32)	*0.195*
**45**	458	(30)	*0.066*	404	(11)	*0.026*	366	(9)	*0.025*	420	(20)	*0.046*
**46**	710	(7)	*0.011*	640	(16)	*0.024*	579	(13)	*0.022*	656	(9)	*0.013*
**47**	662	(94)	*0.142*	644	(104)	*0.162*	583	(67)	*0.115*	640	(89)	*0.138*

						**$\alpha_4\beta_2$**						
**Region**	**supragranular**	**(SD)**	***CV***	**granular**	**(SD)**	***CV***	**infragranular**	**(SD)**	***CV***	**all layers**	**(SD)**	***CV***
**1**	33	(5)	*0.157*	26	(3)	*0.098*	24	(3)	*0.125*	29	(4)	*0.123*
**2**	30	(8)	*0.268*	28	(8)	*0.268*	25	(1)	*0.010*	28	(6)	*0.207*
**3a**	58	(25)	*0.431*	73	(25)	*0.345*	46	(21)	*0.448*	56	(22)	*0.389*
**3b**	46	(8)	*0.184*	104	(16)	*0.157*	44	(6)	*0.132*	54	(8)	*0.152*
**V1**	36	(6)	*0.023*	53	(4)	*0.128*	28	(2)	*0.007*	39	(3)	*0.123*
**V2d**	39	(7)	*0.002*	28	(8)	*0.019*	36	(4)	*0.004*	37	(5)	*0.022*
**V2v**	39	(6)	*0.024*	41	(3)	*0.047*	39	(1)	*0.024*	39	(7)	*0.087*
**V3A**	45	(11)	*0.172*	39	(9)	*0.130*	41	(3)	*0.089*	43	(16)	*0.091*
**V3d**	33	(6)	*0.024*	40	(2)	*0.017*	27	(9)	*0.071*	32	(7)	*0.014*
**V3v**	44	(2)	*0.028*	48	(6)	*0.026*	50	(12)	*0.054*	47	(7)	*0.020*
**V4v**	38	(3)	*0.061*	40	(7)	*0.091*	35	(2)	*0.049*	37	(7)	*0.081*
**FG1**	53	(8)	*0.143*	55	(7)	*0.124*	55	(7)	*0.124*	54	(7)	*0.133*
**FG2**	59	(1)	*0.016*	46	(16)	*0.345*	49	(4)	*0.079*	53	(4)	*0.076*
**37B**	40	(17)	*0.415*	42	(19)	*0.443*	41	(21)	*0.511*	41	(18)	*0.448*
**37L**	37	(13)	*0.360*	36	(9)	*0.252*	36	(10)	*0.268*	36	(11)	*0.312*
**37M**	35	(14)	*0.409*	35	(13)	*0.367*	34	(14)	*0.412*	34	(14)	*0.402*
**41**	49	(33)	*0.676*	86	(44)	*0.515*	48	(29)	*0.597*	54	(32)	*0.593*
**42**	38	(29)	*0.650*	38	(31)	*0.711*	37	(33)	*0.788*	38	(30)	*0.688*
**20**	28	(26)	*0.006*	35	(43)	*0.001*	31	(37)	*0.043*	31	(33)	*0.008*
**21**	34	(13)	*0.684*	35	(32)	*0.726*	33	(31)	*0.752*	34	(30)	*0.711*
**22**	31	(25)	*0.791*	29	(24)	*0.836*	30	(27)	*0.901*	30	(25)	*0.831*

**36**	34	(16)	*0.470*	36	(14)	*0.400*	34	(19)	*0.557*	34	(17)	*0.506*
**38**	39	(5)	*0.116*	40	(3)	*0.124*	38	(3)	*0.193*	39	(4)	*0.101*
**5L**	39	(28)	*0.708*	39	(30)	*0.763*	37	(26)	*0.706*	39	(28)	*0.715*
**5M**	41	(25)	*0.615*	40	(26)	*0.647*	42	(29)	*0.698*	41	(27)	*0.647*
**PGa**	40	(18)	*0.457*	42	(21)	*0.488*	46	(23)	*0.496*	43	(20)	*0.473*
**PGp**	46	(21)	*0.470*	49	(22)	*0.451*	51	(26)	*0.505*	48	(23)	*0.479*
**PFt**	27	(18)	*0.641*	30	(21)	*0.687*	30	(20)	*0.690*	28	(19)	*0.664*
**PFm**	33	(15)	*0.459*	32	(15)	*0.461*	35	(17)	*0.491*	33	(16)	*0.470*
**24**	75	(38)	*0.802*	--	--	--	73	(17)	*0.787*	74	(59)	*0.795*
**32**	138	(60)	*0.019*	131	(57)	*0.091*	106	(45)	*0.063*	124	(53)	*0.011*
**23**	59	(22)	*0.377*	65	(27)	*0.410*	51	(23)	*0.444*	58	(22)	*0.380*
**31**	44	(17)	*0.378*	49	(20)	*0.412*	46	(17)	*0.377*	45	(17)	*0.383*
**4**	85	(19)	*0.141*	78	(12)	*0.250*	62	(8)	*0.212*	69	(13)	*0.139*
**6**	37	(12)	*0.053*	42	(23)	*0.408*	31	(18)	*0.023*	34	(15)	*0.049*
**8**	120	(53)	*0.432*	108	(33)	*0.801*	94	(11)	*0.540*	109	(51)	*0.372*
**9**	71	(5)	*0.068*	76	(58)	*0.373*	68	(15)	*0.285*	71	(11)	*0.179*
**10L**	60	(45)	*0.084*	67	(41)	*0.018*	71	(15)	*0.102*	65	(44)	*0.019*
**10M**	62	(54)	*0.222*	69	(51)	*0.139*	76	(56)	*0.141*	68	(54)	*0.172*
**11**	59	(37)	*0.010*	63	(39)	*0.023*	61	(42)	*0.273*	60	(39)	*0.083*
**44**	51	(16)	*0.426*	51	(15)	*0.418*	49	(12)	*0.501*	47	(13)	*0.451*
**45**	48	(5)	*0.675*	50	(6)	*0.787*	44	(44)	*0.525*	47	(49)	*0.633*
**46**	109	(30)	*0.249*	109	(42)	*0.236*	110	(46)	*0.125*	109	(51)	*0.202*
**47**	87	(31)	*0.103*	85	(10)	*0.034*	95	(15)	*0.017*	89	(15)	*0.124*

α$_1$

**Region**	**supragranular**	**(SD)**	***CV***	**granular**	**(SD)**	***CV***	**infragranular**	**(SD)**	***CV***	**all layers**	**(SD)**	***CV***
**1**	316	(149)	*0.471*	185	(99)	*0.534*	225	(116)	*0.518*	272	(136)	*0.499*
**2**	367	(174)	*0.070*	244	(145)	*0.129*	289	(189)	*0.158*	328	(175)	*0.095*
**3a**	349	(184)	*0.251*	225	(125)	*0.242*	216	(127)	*0.294*	292	(162)	*0.251*
**3b**	290	(142)	*0.232*	172	(100)	*0.237*	176	(92)	*0.290*	244	(117)	*0.232*

**V1**	305	(38)	*0.126*	230	(41)	*0.180*	191	(27)	*0.141*	262	(45)	*0.178*
**V2d**	412	(124)	*0.302*	238	(92)	*0.386*	237	(108)	*0.454*	322	(113)	*0.251*
**V2v**	394	(69)	*0.175*	246	(35)	*0.143*	253	(58)	*0.228*	323	(39)	*0.196*
**V3A**	313	(4)	*0.013*	219	(3)	*0.013*	259	(10)	*0.039*	284	(6)	*0.134*
**V3d**	324	(21)	*0.065*	201	(26)	*0.128*	217	(59)	*0.273*	277	(36)	*0.140*
**V3v**	346	(6)	*0.017*	210	(32)	*0.152*	248	(101)	*0.407*	301	(28)	*0.166*
**V4v**	290	(100)	*0.346*	239	(87)	*0.363*	248	(98)	*0.397*	266	(99)	*0.254*
**FG1**	297	(71)	*0.239*	224	(24)	*0.109*	289	(27)	*0.092*	287	(47)	*0.164*
**FG2**	262	(119)	*0.454*	144	(77)	*0.531*	193	(109)	*0.568*	219	(107)	*0.484*
**37B**	310	(80)	*0.259*	233	(49)	*0.211*	296	(93)	*0.315*	297	(82)	*0.275*
**37L**	327	(67)	*0.204*	232	(75)	*0.324*	304	(98)	*0.321*	307	(76)	*0.247*
**37M**	338	(103)	*0.303*	237	(92)	*0.386*	305	(95)	*0.311*	312	(99)	*0.316*
**41**	257	(145)	*0.370*	157	(109)	*0.407*	182	(115)	*0.374*	223	(132)	*0.378*
**42**	332	(163)	*0.491*	211	(137)	*0.651*	235	(153)	*0.650*	289	(156)	*0.540*
**20**	654	(753)	*0.526*	532	(622)	*0.699*	684	(783)	*0.682*	648	(746)	*0.604*
**21**	655	(781)	*0.536*	534	(639)	*0.591*	702	(831)	*0.632*	656	(775)	*0.581*
**22**	658	(773)	*0.545*	478	(637)	*0.712*	575	(733)	*0.712*	608	(741)	*0.611*
**36**	707	(862)	*0.447*	606	(751)	*0.645*	724	(824)	*0.533*	701	(836)	*0.494*
**38**	324	(124)	*0.219*	277	(84)	*0.194*	320	(128)	*0.153*	317	(121)	*0.193*
**5L**	348	(170)	*0.209*	240	(147)	*0.348*	257	(150)	*0.289*	309	(160)	*0.242*
**5M**	368	(185)	*0.501*	235	(130)	*0.554*	272	(172)	*0.633*	317	(172)	*0.545*
**PGa**	327	(163)	*0.223*	219	(121)	*0.412*	282	(156)	*0.363*	299	(157)	*0.286*
**PGp**	331	(153)	*0.092*	230	(116)	*0.160*	280	(150)	*0.154*	303	(149)	*0.109*
**PFt**	306	(103)	*0.337*	220	(67)	*0.305*	275	(109)	*0.397*	284	(99)	*0.347*
**PFm**	353	(114)	*0.211*	222	(84)	*0.232*	304	(109)	*0.238*	321	(106)	*0.211*
**24**	547	(275)	*0.012*	--	--	--	506	(228)	*0.042*	526	(250)	*0.028*
**32**	433	(254)	*0.047*	349	(196)	*0.067*	357	(193)	*0.089*	395	(223)	*0.069*
**23**	318	(129)	*0.234*	204	(72)	*0.160*	234	(75)	*0.213*	277	(104)	*0.216*
**31**	431	(162)	*0.324*	313	(134)	*0.367*	322	(143)	*0.288*	379	(149)	*0.307*
**4**	263	(13)	*0.424*	179	(0)	*0.187*	155	(3)	*0.595*	193	(7)	*0.478*

**6**	2287	(209)	*0.467*	1750	(383)	*0.592*	1563	(264)	*0.422*	1880	(232)	*0.443*
**8**	361	(169)	*0.097*	271	(121)	*0.183*	298	(141)	*0.156*	326	(148)	*0.137*
**9**	319	(219)	*0.073*	255	(176)	*0.099*	289	(201)	*0.131*	300	(205)	*0.096*
**10L**	341	(226)	*0.077*	252	(136)	*0.033*	255	(142)	*0.028*	302	(188)	*0.058*
**10M**	336	(199)	*0.045*	259	(128)	*0.011*	269	(144)	*0.014*	304	(172)	*0.030*
**11**	385	(279)	*0.053*	308	(230)	*0.067*	302	(224)	*0.009*	349	(253)	*0.041*
**44**	208	(20)	*0.447*	140	(20)	*0.401*	158	(7)	*0.527*	126	(9)	*0.466*
**45**	239	(174)	*0.344*	144	(95)	*0.337*	146	(96)	*0.278*	200	(143)	*0.347*
**46**	347	(175)	*0.076*	242	(97)	*0.009*	268	(109)	*0.007*	308	(145)	*0.049*
**47**	337	(143)	*0.087*	249	(88)	*0.100*	281	(116)	*0.125*	308	(124)	*0.095*

						**α_2**						
**Region**	**supragranular**	**(SD)**	***CV***	**granular**	**(SD)**	***CV***	**infragranular**	**(SD)**	***CV***	**all layers**	**(SD)**	***CV***
**1**	258	(108)	*0.420*	222	(103)	*0.466*	190	(85)	*0.444*	232	(103)	*0.443*
**2**	251	(126)	*0.370*	233	(138)	*0.442*	192	(108)	*0.374*	230	(121)	*0.378*
**3a**	284	(126)	*0.358*	213	(105)	*0.434*	180	(95)	*0.482*	244	(116)	*0.401*
**3b**	366	(144)	*0.366*	360	(156)	*0.420*	241	(99)	*0.413*	332	(132)	*0.377*
**V1**	417	(176)	*0.422*	349	(148)	*0.425*	164	(76)	*0.463*	353	(161)	*0.342*
**V2d**	275	(161)	*0.586*	304	(221)	*0.728*	249	(187)	*0.753*	267	(177)	*0.497*
**V2v**	241	(114)	*0.473*	273	(132)	*0.484*	205	(133)	*0.646*	230	(116)	*0.415*
**V3A**	243	(144)	*0.591*	235	(158)	*0.674*	208	(168)	*0.812*	230	(152)	*0.512*
**V3d**	234	(132)	*0.563*	250	(128)	*0.514*	203	(112)	*0.554*	225	(124)	*0.462*
**V3v**	233	(96)	*0.413*	258	(86)	*0.332*	195	(73)	*0.377*	219	(82)	*0.416*
**V4v**	208	(79)	*0.382*	248	(137)	*0.553*	156	(99)	*0.636*	185	(96)	*0.366*
**FG1**	165	(3)	*0.017*	149	(6)	*0.043*	105	(25)	*0.239*	137	(10)	*0.074*
**FG2**	159	(2)	*0.010*	168	(31)	*0.186*	116	(10)	*0.088*	138	(11)	*0.078*
**37B**	174	(70)	*0.334*	181	(84)	*0.374*	141	(67)	*0.365*	162	(72)	*0.354*
**37L**	151	(80)	*0.526*	157	(95)	*0.603*	134	(84)	*0.628*	146	(82)	*0.560*
**37M**	167	(97)	*0.580*	170	(113)	*0.662*	119	(78)	*0.656*	149	(93)	*0.623*
**41**	288	(149)	*0.366*	271	(192)	*0.450*	214	(139)	*0.419*	263	(152)	*0.386*

						5-HT_{1A}						
**Region**	**supragranular**	**(SD)**	***CV***	**granular**	**(SD)**	***CV***	**infragranular**	**(SD)**	***CV***	**all layers**	**(SD)**	***CV***
**42**	234	(130)	*0.554*	210	(135)	*0.643*	162	(95)	*0.587*	210	(119)	*0.568*
**20**	177	(82)	*0.460*	188	(103)	*0.549*	153	(87)	*0.570*	169	(86)	*0.511*
**21**	198	(137)	*0.557*	200	(147)	*0.622*	157	(110)	*0.585*	184	(129)	*0.570*
**22**	216	(131)	*0.608*	200	(123)	*0.615*	148	(84)	*0.567*	190	(114)	*0.598*
**36**	183	(92)	*0.500*	203	(117)	*0.579*	157	(90)	*0.577*	175	(94)	*0.536*
**38**	137	(93)	*0.138*	141	(78)	*0.062*	110	(65)	*0.068*	127	(80)	*0.103*
**5L**	173	(80)	*0.464*	153	(80)	*0.524*	127	(71)	*0.557*	157	(76)	*0.485*
**5M**	175	(80)	*0.458*	149	(81)	*0.542*	135	(76)	*0.560*	159	(77)	*0.486*
**PGa**	168	(51)	*0.305*	165	(52)	*0.317*	132	(49)	*0.373*	155	(51)	*0.327*
**PGp**	172	(81)	*0.470*	170	(85)	*0.501*	137	(73)	*0.537*	159	(79)	*0.494*
**PFt**	152	(72)	*0.470*	151	(78)	*0.515*	125	(70)	*0.557*	142	(72)	*0.507*
**PFm**	174	(82)	*0.225*	169	(82)	*0.303*	136	(62)	*0.287*	159	(73)	*0.240*
**24**	290	(173)	*0.094*	--	--	--	188	(115)	*0.204*	242	(145)	*0.105*
**32**	331	(235)	*0.010*	239	(164)	*0.167*	203	(138)	*0.192*	269	(180)	*0.062*
**23**	145	(44)	*0.308*	129	(46)	*0.361*	96	(35)	*0.367*	127	(40)	*0.319*
**31**	169	(40)	*0.235*	158	(50)	*0.317*	132	(40)	*0.302*	156	(40)	*0.255*
**4**	152	(9)	*0.425*	97	(23)	*0.276*	86	(23)	*0.381*	111	(15)	*0.406*
**6**	120	(24)	*0.236*	89	(30)	*0.119*	69	(19)	*0.234*	92	(19)	*0.240*
**8**	266	(176)	*0.198*	204	(115)	*0.250*	177	(120)	*0.323*	225	(145)	*0.225*
**9**	215	(169)	*0.024*	180	(128)	*0.014*	145	(112)	*0.033*	185	(141)	*0.023*
**10L**	193	(134)	*0.105*	181	(104)	*0.030*	145	(98)	*0.090*	175	(119)	*0.094*
**10M**	196	(127)	*0.161*	183	(103)	*0.122*	146	(89)	*0.082*	177	(111)	*0.143*
**11**	225	(196)	*0.070*	221	(180)	*0.052*	174	(154)	*0.090*	207	(179)	*0.033*
**44**	226	(13)	*0.010*	180	(25)	*0.004*	160	(15)	*0.044*	179	(15)	*0.021*
**45**	186	(154)	*0.285*	155	(122)	*0.260*	132	(107)	*0.242*	165	(136)	*0.284*
**46**	239	(169)	*0.087*	212	(129)	*0.064*	177	(110)	*0.099*	215	(145)	*0.083*
**47**	212	(131)	*0.107*	189	(101)	*0.075*	158	(89)	*0.131*	193	(114)	*0.103*

**1**	608	(401)	*0.393*	104	(50)	*0.257*	170	(52)	*0.245*	410	(269)	*0.356*
**2**	780	(515)	*0.660*	186	(91)	*0.491*	259	(167)	*0.646*	576	(321)	*0.557*
**3a**	591	(445)	*0.434*	108	(66)	*0.067*	148	(101)	*0.500*	420	(312)	*0.363*
**3b**	536	(378)	*0.316*	84	(35)	*0.185*	116	(63)	*0.603*	354	(213)	*0.259*
**V1**	433	(296)	*0.684*	72	(28)	*0.391*	37	(3)	*0.077*	267	(198)	*0.657*
**V2d**	537	(225)	*0.420*	124	(98)	*0.785*	84	(39)	*0.463*	306	(131)	*0.475*
**V2v**	588	(99)	*0.168*	95	(31)	*0.322*	94	(61)	*0.648*	341	(3)	*0.256*
**V3A**	570	(340)	*0.596*	63	(23)	*0.364*	107	(73)	*0.679*	345	(198)	*0.419*
**V3d**	534	(222)	*0.415*	75	(22)	*0.294*	85	(24)	*0.287*	342	(143)	*0.376*
**V3v**	614	(60)	*0.097*	119	(76)	*0.640*	104	(26)	*0.250*	366	(14)	*0.250*
**V4v**	750	(649)	*0.866*	206	(198)	*0.961*	151	(87)	*0.578*	428	(359)	*0.459*
**FG1**	377	(96)	*0.253*	124	(75)	*0.606*	169	(137)	*0.809*	268	(20)	*0.073*
**FG2**	525	(154)	*0.294*	115	(87)	*0.752*	129	(69)	*0.537*	308	(137)	*0.442*
**37B**	665	(406)	*0.611*	215	(86)	*0.400*	284	(151)	*0.530*	555	(187)	*0.337*
**37L**	573	(343)	*0.598*	156	(90)	*0.578*	253	(118)	*0.466*	450	(192)	*0.427*
**37M**	758	(454)	*0.304*	166	(89)	*0.343*	282	(108)	*0.421*	501	(281)	*0.268*
**41**	695	(624)	*0.546*	93	(49)	*0.172*	142	(85)	*0.341*	482	(366)	*0.450*
**42**	838	(699)	*0.496*	127	(76)	*0.254*	186	(131)	*0.197*	602	(427)	*0.301*
**20**	764	(327)	*0.428*	236	(77)	*0.326*	379	(169)	*0.447*	615	(221)	*0.359*
**21**	872	(473)	*0.503*	246	(95)	*0.313*	406	(212)	*0.318*	664	(316)	*0.413*
**22**	725	(456)	*0.300*	163	(91)	*0.250*	286	(160)	*0.268*	509	(315)	*0.239*
**36**	947	(485)	*0.524*	281	(90)	*0.300*	382	(142)	*0.332*	688	(273)	*0.366*
**38**	834	(432)	*0.059*	288	(94)	*0.040*	391	(142)	*0.025*	603	(279)	*0.056*
**5L**	616	(306)	*0.393*	164	(72)	*0.230*	210	(72)	*0.247*	441	(176)	*0.263*
**5M**	581	(148)	*0.256*	122	(32)	*0.263*	203	(67)	*0.328*	397	(108)	*0.271*
**PGa**	516	(251)	*0.204*	163	(76)	*0.131*	245	(86)	*0.116*	369	(191)	*0.244*
**PGp**	502	(275)	*0.315*	179	(89)	*0.251*	230	(110)	*0.341*	381	(185)	*0.224*
**PFt**	657	(301)	*0.396*	217	(76)	*0.328*	313	(88)	*0.280*	493	(185)	*0.312*
**PFm**	631	(332)	*0.448*	191	(108)	*0.357*	283	(126)	*0.351*	488	(194)	*0.275*
**24**	776	(536)	*0.691*	--	--	--	370	(174)	*0.471*	568	(338)	*0.596*

**32**	653	(401)	*0.150*	211	(98)	*0.166*	268	(114)	*0.005*	453	(245)	*0.090*
**23**	395	(127)	*0.150*	128	(28)	*0.172*	226	(63)	*0.197*	305	(92)	*0.147*
**31**	542	(245)	*0.452*	167	(61)	*0.363*	229	(129)	*0.564*	430	(139)	*0.324*
**4**	291	(60)	*0.615*	73	(8)	*0.078*	94	(1)	*0.494*	162	(31)	*0.592*
**6**	357	(59)	*0.582*	113	(34)	*1.069*	140	(49)	*0.967*	238	(51)	*0.696*
**8**	528	(122)	*0.231*	221	(87)	*0.393*	279	(90)	*0.321*	408	(101)	*0.247*
**9**	572	(220)	*0.006*	214	(188)	*0.149*	313	(190)	*0.015*	444	(204)	*0.022*
**10L**	634	(285)	*0.143*	193	(73)	*0.082*	280	(109)	*0.157*	458	(198)	*0.139*
**10M**	605	(252)	*0.048*	208	(95)	*0.273*	301	(95)	*0.108*	444	(164)	*0.093*
**11**	667	(269)	*0.065*	234	(110)	*0.294*	331	(126)	*0.043*	505	(190)	*0.105*
**44**	491	(64)	*0.177*	158	(54)	*0.031*	228	(46)	*0.296*	284	(38)	*0.204*
**45**	573	(484)	*0.414*	132	(102)	*0.284*	173	(111)	*0.321*	404	(346)	*0.445*
**46**	674	(409)	*0.203*	202	(112)	*0.124*	326	(141)	*0.177*	496	(284)	*0.190*
**47**	672	(308)	*0.052*	228	(90)	*0.021*	321	(121)	*0.006*	505	(208)	*0.041*

						**$5\text{-}HT_2$**						
**Region**	**supragranular**	**(SD)**	***CV***	**granular**	**(SD)**	***CV***	**infragranular**	**(SD)**	***CV***	**all layers**	**(SD)**	***CV***
**1**	382	(135)	*0.352*	329	(105)	*0.320*	224	(63)	*0.282*	332	(105)	*0.316*
**2**	426	(144)	*0.339*	380	(143)	*0.377*	264	(128)	*0.485*	374	(126)	*0.337*
**3a**	423	(148)	*0.351*	377	(156)	*0.414*	263	(108)	*0.411*	338	(121)	*0.358*
**3b**	482	(173)	*0.144*	467	(178)	*0.272*	246	(115)	*0.424*	411	(157)	*0.197*
**V1**	551	(264)	*0.480*	601	(262)	*0.459*	263	(106)	*0.403*	506	(241)	*0.444*
**V2d**	520	(133)	*0.256*	473	(89)	*0.187*	233	(16)	*0.071*	393	(74)	*0.330*
**V2v**	508	(82)	*0.162*	570	(221)	*0.368*	350	(121)	*0.345*	451	(78)	*0.310*
**V3A**	397	(56)	*0.141*	436	(85)	*0.196*	277	(13)	*0.048*	354	(34)	*0.245*
**V3d**	418	(81)	*0.194*	337	(58)	*0.172*	254	(58)	*0.228*	357	(21)	*0.230*
**V3v**	521	(103)	*0.198*	504	(125)	*0.247*	238	(62)	*0.258*	404	(56)	*0.385*
**V4v**	454	(101)	*0.221*	481	(284)	*0.591*	285	(160)	*0.562*	374	(149)	*0.300*
**FG1**	257	(41)	*0.109*	157	(16)	*0.115*	116	(33)	*0.045*	187	(37)	*0.123*
**FG2**	318	(71)	*0.009*	278	(19)	*0.009*	203	(33)	*0.005*	253	(25)	*0.023*

**37B**	460	(213)	*0.462*	323	(152)	*0.470*	261	(64)	*0.245*	301	(107)	*0.354*
**37L**	416	(150)	*0.361*	372	(154)	*0.413*	260	(105)	*0.405*	343	(122)	*0.357*
**37M**	422	(155)	*0.368*	383	(187)	*0.489*	294	(175)	*0.597*	350	(170)	*0.485*
**41**	481	(188)	*0.392*	469	(273)	*0.582*	264	(150)	*0.569*	388	(193)	*0.496*
**42**	502	(179)	*0.356*	405	(175)	*0.433*	291	(119)	*0.409*	389	(164)	*0.421*
**20**	508	(256)	*0.505*	379	(138)	*0.364*	301	(139)	*0.463*	372	(139)	*0.372*
**21**	516	(182)	*0.378*	427	(137)	*0.297*	332	(112)	*0.233*	410	(131)	*0.262*
**22**	442	(143)	*0.324*	358	(148)	*0.413*	268	(108)	*0.402*	346	(141)	*0.408*
**36**	464	(177)	*0.382*	387	(212)	*0.547*	346	(172)	*0.497*	375	(198)	*0.527*
**38**	457	(282)	*0.107*	415	(246)	*0.038*	300	(167)	*0.130*	392	(232)	*0.020*
**5L**	395	(174)	*0.387*	425	(181)	*0.285*	302	(160)	*0.494*	387	(152)	*0.308*
**5M**	429	(135)	*0.315*	418	(197)	*0.472*	309	(159)	*0.514*	377	(150)	*0.397*
**PGa**	413	(190)	*0.451*	430	(214)	*0.425*	318	(160)	*0.466*	382	(179)	*0.436*
**PGp**	362	(160)	*0.188*	369	(204)	*0.203*	234	(107)	*0.128*	325	(144)	*0.168*
**PFt**	461	(99)	*0.215*	379	(154)	*0.407*	308	(88)	*0.285*	357	(120)	*0.336*
**PFm**	461	(152)	*0.330*	406	(204)	*0.503*	305	(126)	*0.412*	368	(155)	*0.423*
**24**	435	(214)	*0.077*	--	--	--	367	(209)	*0.055*	401	(209)	*0.051*
**32**	400	(254)	*0.018*	410	(261)	*0.028*	309	(189)	*0.008*	367	(227)	*0.001*
**23**	447	(190)	*0.424*	434	(199)	*0.459*	304	(119)	*0.393*	399	(169)	*0.423*
**31**	503	(173)	*0.377*	475	(236)	*0.500*	361	(164)	*0.500*	413	(188)	*0.482*
**4**	300	(10)	*0.292*	265	(8)	*0.251*	194	(5)	*0.385*	234	(2)	*0.320*
**6**	227	(24)	*0.660*	221	(41)	*0.399*	180	(38)	*0.676*	203	(31)	*0.700*
**8**	354	(140)	*0.028*	329	(122)	*0.027*	249	(73)	*0.075*	313	(114)	*0.050*
**9**	330	(125)	*0.063*	296	(101)	*0.014*	229	(72)	*0.036*	289	(101)	*0.017*
**10L**	320	(74)	*0.020*	317	(68)	*0.011*	251	(60)	*0.039*	296	(69)	*0.017*
**10M**	315	(99)	*0.190*	325	(95)	*0.171*	260	(79)	*0.141*	297	(90)	*0.174*
**11**	360	(152)	*0.150*	359	(161)	*0.158*	282	(122)	*0.222*	334	(141)	*0.165*
**44**	317	(10)	*0.081*	312	(27)	*0.072*	235	(23)	*0.053*	271	(11)	*0.020*
**45**	295	(201)	*0.213*	282	(205)	*0.282*	211	(167)	*0.262*	266	(192)	*0.256*
**46**	388	(182)	*0.046*	365	(190)	*0.004*	292	(153)	*0.004*	352	(174)	*0.025*

**47**	393	(173)	*0.126*	384	(190)	*0.103*	307	(146)	*0.119*	366	(163)	*0.120*

D_1

**Region**	**supragranular**	**(SD)**	***CV***	**granular**	**(SD)**	***CV***	**infragranular**	**(SD)**	***CV***	**all layers**	**(SD)**	***CV***
**1**	87	(32)	*0.364*	60	(24)	*0.393*	48	(24)	*0.512*	72	(28)	*0.382*
**2**	99	(25)	*0.255*	81	(25)	*0.311*	68	(21)	*0.303*	87	(24)	*0.279*
**3a**	76	(22)	*0.286*	59	(14)	*0.231*	44	(15)	*0.335*	65	(18)	*0.281*
**3b**	108	(24)	*0.224*	80	(30)	*0.372*	57	(16)	*0.285*	90	(22)	*0.250*
**V1**	135	(74)	*0.552*	122	(58)	*0.479*	87	(16)	*0.277*	118	(55)	*0.366*
**V2d**	133	(50)	*0.372*	99	(22)	*0.222*	76	(11)	*0.145*	106	(30)	*0.300*
**V2v**	132	(43)	*0.326*	121	(55)	*0.456*	59	(34)	*0.391*	111	(32)	*0.286*
**V3A**	118	(37)	*0.312*	88	(16)	*0.187*	76	(15)	*0.194*	99	(25)	*0.282*
**V3d**	114	(34)	*0.297*	95	(28)	*0.292*	72	(15)	*0.204*	98	(27)	*0.273*
**V3v**	128	(50)	*0.390*	113	(51)	*0.448*	70	(13)	*0.180*	102	(28)	*0.306*
**V4v**	115	(39)	*0.337*	114	(50)	*0.439*	70	(28)	*0.399*	93	(36)	*0.280*
**FG1**	69	(13)	*0.189*	59	(1)	*0.003*	38	(9)	*0.239*	55	(3)	*0.047*
**FG2**	85	(8)	*0.091*	72	(11)	*0.160*	57	(12)	*0.203*	70	(12)	*0.168*
**37B**	97	(27)	*0.276*	82	(18)	*0.221*	71	(20)	*0.279*	86	(23)	*0.267*
**37L**	91	(27)	*0.292*	75	(23)	*0.308*	66	(17)	*0.250*	81	(23)	*0.288*
**37M**	97	(27)	*0.282*	78	(21)	*0.273*	71	(24)	*0.337*	84	(26)	*0.303*
**41**	86	(26)	*0.304*	68	(20)	*0.298*	53	(17)	*0.317*	74	(22)	*0.299*
**42**	79	(8)	*0.099*	61	(6)	*0.091*	48	(5)	*0.114*	67	(5)	*0.070*
**20**	68	(21)	*0.313*	58	(18)	*0.311*	52	(16)	*0.305*	61	(18)	*0.290*
**21**	78	(24)	*0.303*	69	(22)	*0.313*	56	(17)	*0.310*	69	(21)	*0.299*
**22**	68	(17)	*0.245*	51	(14)	*0.273*	43	(12)	*0.281*	57	(14)	*0.237*
**36**	74	(29)	*0.351*	64	(26)	*0.351*	60	(24)	*0.326*	67	(26)	*0.334*
**38**	111	(47)	*0.074*	100	(39)	*0.122*	79	(33)	*0.323*	97	(41)	*0.166*
**5L**	88	(46)	*0.515*	72	(39)	*0.545*	58	(32)	*0.554*	77	(40)	*0.519*
**5M**	85	(46)	*0.538*	70	(36)	*0.515*	56	(27)	*0.480*	74	(38)	*0.506*
**PGa**	75	(36)	*0.471*	64	(27)	*0.423*	54	(23)	*0.416*	67	(31)	*0.458*

**PGp**	96	(34)	*0.356*	82	(24)	*0.296*	64	(16)	*0.249*	84	(27)	*0.327*
**PFt**	84	(37)	*0.440*	71	(29)	*0.402*	58	(26)	*0.440*	73	(32)	*0.436*
**PFm**	87	(41)	*0.478*	74	(40)	*0.538*	63	(30)	*0.471*	77	(36)	*0.468*
**24**	111	(35)	*0.314*	--	--	--	81	(27)	*0.326*	97	(30)	*0.312*
**32**	93	(43)	*0.482*	90	(37)	*0.052*	70	(25)	*0.507*	83	(35)	*0.357*
**23**	86	(23)	*0.265*	70	(17)	*0.238*	55	(18)	*0.323*	74	(20)	*0.264*
**31**	93	(45)	*0.110*	81	(37)	*0.124*	66	(34)	*0.158*	83	(40)	*0.115*
**4**	61	(6)	*0.563*	54	(1)	*0.075*	42	(2)	*0.650*	49	(5)	*0.601*
**6**	36	(7)	*0.490*	32	(5)	*0.172*	24	(4)	*0.518*	30	(6)	*0.502*
**8**	81	(21)	*0.266*	76	(18)	*0.234*	60	(14)	*0.229*	72	(17)	*0.230*
**9**	86	(17)	*0.201*	82	(11)	*0.138*	63	(9)	*0.148*	77	(14)	*0.183*
**10L**	84	(9)	*0.103*	81	(6)	*0.076*	63	(10)	*0.160*	76	(9)	*0.117*
**10M**	78	(11)	*0.140*	85	(9)	*0.109*	66	(12)	*0.181*	74	(10)	*0.133*
**11**	91	(17)	*0.188*	92	(19)	*0.201*	72	(20)	*0.280*	85	(18)	*0.209*
**44**	74	(23)	*0.453*	71	(18)	*0.370*	54	(17)	*0.406*	62	(20)	*0.426*
**45**	76	(58)	*0.523*	69	(46)	*0.476*	56	(38)	*0.546*	69	(51)	*0.531*
**46**	80	(20)	*0.253*	74	(17)	*0.227*	61	(13)	*0.213*	73	(17)	*0.239*
**47**	75	(15)	*0.005*	74	(12)	*0.045*	62	(11)	*0.016*	71	(13)	*0.009*
