# Supplementary material for: Multiple Transmitter Receptors in Regions and Layers of the Human Cerebral Cortex
Source: Front Neuroanat. 2017 Sep 20;11:78. doi: 10.3389/fnana.2017.00078 (PMC5609104; doi:10.3389/fnana.2017.00078)
Supplement: Supplementary file 3 [file Table_3.pdf]

Supplementary Table 3:

P values resulting from the discriminant analyses of fingerprints of mean areal densities (averaged over all layers). Red codes p ≤ 0.01, orange codes p ≤ 0.05.

	1	2	3a	3b	V1	V2d	V2v	V3A	V3d	V3v	V4v	FG1	FG2	37B	37L	37M	41	42	20	21	22	36	38	5L	5M	PGa	PGp	PFt	PFm	24	32	23	31	4	6	8	9	10L	10M	11	44	45	46	47	
1	--	0.70	0.82	0.50	0.76	0.72	0.54	0.09	0.24	0.35	0.60	0.21	0.20	0.33	0.71	0.34	0.64	0.55	0.99	0.92	0.11	0.48	0.19	0.61	0.84	0.77	0.22	0.33	0.18	0.02	0.18	0.02	0.78	0.28	0.32	0.62	0.16	0.64	0.63	0.27	0.20	0.34	0.07	0.25	1
2		--	0.69	0.24	0.28	0.17	0.23	0.54	0.14	0.61	0.54	0.54	0.17	0.60	0.26	0.32	0.35	0.25	0.51	0.85	0.91	0.67	0.14	0.93	0.89	0.28	0.21	0.11	0.41	0.08	0.32	0.14	0.90	0.02	0.07	0.32	0.02	1.00	0.99	0.20	0.14	0.35	0.04	0.16	2
3a			--	0.54	0.69	0.20	0.73	0.92	0.97	0.20	0.98	0.63	0.60	0.12	0.80	0.31	0.09	0.12	0.69	0.72	0.04	0.67	0.13	0.47	0.74	0.62	0.66	0.29	0.53	0.24	0.20	0.14	0.62	0.60	0.39	0.90	0.39	0.32	0.47	0.24	0.77	0.11	0.45	0.31	3a
3b				--	0.20	0.00	0.37	0.07	0.04	0.04	0.49	0.33	0.07	0.16	0.09	0.02	0.02	0.33	0.16	0.33	0.37	0.48	0.02	0.01	0.02	0.09	0.09	0.06	0.24	0.27	0.02	0.02	0.55	0.01	0.01	0.31	0.22	0.31	0.38	0.05	0.02	0.04	0.28	0.10	3b
V1					--	0.15	0.18	0.53	0.52	0.17	0.04	0.84	0.02	0.01	0.44	0.14	0.02	0.48	0.34	0.05	0.02	0.05	0.04	0.02	0.54	0.40	0.02	0.08	0.72	0.15	0.42	0.65	0.13	0.01	0.00	0.81	0.75	0.12	0.62	0.27	0.21	0.34	0.07	0.14	V1
V2d						--	1.00	0.50	0.78	0.89	0.66	0.57	0.81	0.33	0.34	0.07	0.22	0.32	0.19	0.27	0.29	0.14	0.74	0.77	0.78	0.35	0.97	0.23	0.83	0.36	0.18	0.41	0.06	0.14	0.56	0.66	0.62	0.69	0.83	0.12	0.54	0.56	0.61	0.28	V2d
V2v							--	0.86	0.73	0.69	0.55	0.85	0.55	0.30	0.28	0.07	0.19	0.52	0.40	0.51	0.54	0.49	0.15	0.83	0.39	0.20	0.62	0.14	0.37	0.61	0.65	0.34	0.15	0.38	0.45	0.87	0.96	0.77	0.88	0.86	0.09	0.19	0.94	0.83	V2v
V3A								--	0.60	0.67	0.89	0.59	0.38	0.88	0.18	0.10	0.21	0.21	0.09	0.13	0.19	0.33	0.07	0.14	0.18	0.21	0.75	0.20	0.32	0.42	0.26	0.32	0.36	0.20	0.27	0.34	0.91	0.60	0.29	0.16	0.39	0.11	0.89	0.73	V3A
V3d									--	0.59	0.89	0.78	0.29	0.20	0.17	0.11	0.18	0.31	0.11	0.16	0.34	0.37	0.58	0.60	0.22	0.19	0.48	0.38	0.41	0.34	0.33	0.32	0.15	0.17	0.09	0.54	0.48	0.74	0.63	0.36	0.13	0.25	0.43	0.32	V3d
V3v										--	0.62	0.14	0.09	0.30	0.09	0.07	0.09	0.59	0.09	0.24	0.77	0.48	0.10	0.91	0.53	0.09	0.18	0.08	0.46	0.69	0.22	0.64	0.58	0.27	0.17	0.62	0.96	0.58	0.65	0.33	0.01	0.07	0.95	0.67	V3v
V4v											--	0.93	0.40	0.88	0.19	0.23	0.24	0.55	0.26	0.31	0.11	0.81	0.14	0.27	0.64	0.14	0.42	0.24	0.46	0.47	0.48	0.30	0.81	0.40	0.29	0.97	0.91	0.63	0.72	0.92	0.19	0.19	0.86	0.86	V4v
FG1												--	0.46	0.96	0.12	0.28	0.08	0.63	0.10	0.10	0.02	0.90	0.04	0.44	0.42	0.09	0.56	0.10	0.60	0.21	0.13	0.05	0.20	0.25	0.08	0.52	0.83	0.37	0.45	0.75	0.16	0.93	0.70	0.63	FG1
FG2													--	1.00	0.17	0.84	0.10	0.22	0.14	0.15	0.02	0.98	0.04	0.41	0.36	0.13	0.53	0.15	0.98	0.18	0.12	0.05	0.29	0.16	0.08	0.50	0.77	0.35	0.43	0.69	0.49	0.02	0.63	0.58	FG2
37B														--	0.77	0.73	0.24	0.68	0.33	0.84	0.82	0.65	0.51	0.53	0.59	0.42	0.43	0.50	0.92	0.21	0.15	0.02	0.27	0.05	0.08	0.68	0.63	0.37	0.49	0.51	0.19	0.33	0.57	0.50	37B
37L															--	0.93	0.46	0.31	0.85	0.84	0.97	0.88	0.59	0.56	0.96	0.18	0.27	0.50	0.80	0.12	0.64	0.18	0.37	0.20	0.24	0.84	0.38	0.87	0.93	0.40	0.85	0.05	0.35	0.49	37L
37M																--	0.10	0.49	0.83	0.76	0.40	0.90	0.06	0.98	0.97	0.37	0.52	0.49	0.19	0.93	0.09	0.02	0.95	0.17	0.05	0.28	0.69	0.01	0.14	0.10	0.15	0.37	0.87	0.38	37M
41																	--	0.22	0.09	0.14	0.03	0.51	0.09	0.11	0.57	0.29	0.09	0.04	0.11	0.09	0.24	0.14	0.42	0.06	0.08	0.71	0.07	0.14	0.17	0.15	0.04	0.09	0.08	0.09	41
42																		--	0.57	0.80	0.60	0.78	0.09	0.96	0.59	0.30	0.29	0.15	0.50	0.40	0.43	0.49	0.92	0.22	0.25	0.58	0.40	0.81	0.89	0.62	0.08	0.09	0.38	0.62	42
20																			--	0.36	0.11	0.83	0.14	0.43	0.93	0.18	0.78	0.68	0.33	0.09	0.32	0.09	0.33	0.26	0.38	0.70	0.32	0.66	0.77	0.33	0.66	0.11	0.28	0.40	20
21																				--	0.16	0.84	0.18	0.46	0.93	0.24	0.57	0.66	0.54	0.10	0.38	0.11	0.46	0.30	0.33	0.74	0.34	0.72	0.81	0.35	0.79	0.08	0.30	0.42	21
22																					--	0.69	0.04	0.59	0.82	0.07	0.03	0.03	0.02	0.06	0.77	0.13	0.40	0.27	0.03	0.20	0.07	0.23	0.46	0.13	0.01	0.11	0.06	0.11	22
36																						--	0.27	0.81	0.41	0.80	0.98	0.79	0.87	0.95	0.31	0.09	0.77	0.11	0.30	0.64	0.48	0.82	0.35	0.82	0.69	0.63	0.17	0.67	36
38																							--	0.08	0.22	0.09	0.04	0.15	0.03	0.08	0.10	0.09	0.06	0.12	0.10	0.12	0.08	0.10	0.10	0.08	0.68	0.05	0.08	0.08	38
5L																								--	0.87	0.36	0.47	0.44	0.33	0.77	0.71	0.34	0.84	0.20	0.43	0.80	0.80	0.89	0.91	0.86	0.48	0.31	0.80	0.92	5L
5M																									--	0.85	0.40	0.52	0.34	0.21	0.24	0.80	0.48	0.50	0.07	0.94	0.41	0.97	0.98	0.46	0.48	0.21	0.46	0.61	5M
PGa																										--	0.14	0.14	0.57	0.08	0.23	0.07	0.21	0.21	0.63	0.61	0.28	0.55	0.66	0.29	0.45	0.36	0.25	0.35	PGa
PGp																											--	0.28	0.07	0.24	0.14	0.06	0.31	0.18	0.09	0.54	0.88	0.39	0.47	0.80	0.16	0.02	0.76	0.67	PGp
PFt																												--	0.74	0.09	0.35	0.54	0.73	0.12	0.36	0.72	0.33	0.68	0.78	0.34	0.10	0.10	0.29	0.41	PFt
PFm																													--	0.12	0.10	0.04	0.16	0.22	0.07	0.43	0.59	0.29	0.37	0.53	0.12	0.15	0.47	0.47	PFm
24																														--	0.12	0.09	0.58	0.21	0.10	0.14	0.85	0.25	0.24	0.62	0.08	0.02	0.95	0.58	24
32																															--	0.17	0.89	0.21	0.12	0.63	0.55	0.52	0.69	0.14	0.46	0.25	0.07	0.06	32
23																																--	0.73	0.25	0.11	0.12	0.23	0.21	0.22	0.65	0.05	0.02	0.09	0.72	23
31																																	--	0.41	0.31	0.99	0.66	1.00	0.99	0.70	0.37	0.26	0.74	0.85	31
4																																		--	0.66	0.22	0.16	0.23	0.20	0.16	0.50	0.34	0.22	0.23	4
6																																			--	0.18	0.09	0.11	0.12	0.08	0.64	0.23	0.09	0.09	6
8																																				--	0.78	0.73	0.54	0.19	0.10	0.51	0.15	0.12	8
9																																					--	0.22	0.18	0.81	0.07	0.12	0.99	0.50	9
10L																																						--	0.71	0.04	0.05	0.13	0.31	0.15	10L
10M																																							--	0.09	0.06	0.02	0.31	0.06	10M
11																																								--	0.15	0.16	0.89	0.97	11
44																																									--	0.36	0.09	0.73	44
45																																										--	0.02	0.06	45
46																																											--	0.02	46
47																																												--	47
