# Supplementary material for: Multiple Transmitter Receptors in Regions and Layers of the Human Cerebral Cortex
Source: Front Neuroanat. 2017 Sep 20;11:78. doi: 10.3389/fnana.2017.00078 (PMC5609104; doi:10.3389/fnana.2017.00078)
Supplement: Supplementary file 4 [file Table_4.pdf]

Supplementary Table 4:

P values resulting from the discriminant analyses of fingerprints obtained from the supragranular stratum of each area. Red codes p ≤ 0.01, orange codes p ≤ 0.05.

	1	2	3a	3b	V1	V2d	V2v	V3A	V3d	V3v	V4v	FG1	FG2	37B	37L	37M	41	42	20	21	22	36	38	5L	5M	PGa	PGp	PFt	PFm	24	32	23	31	4	6	8	9	10L	10M	11	44	45	46	47
1	--	0.54	0.85	0.09	0.83	0.83	0.59	0.73	0.71	0.61	0.69	0.27	0.28	0.50	0.80	0.30	0.49	0.15	0.85	0.98	0.09	0.65	0.07	0.64	0.80	0.70	0.35	0.29	0.26	0.04	0.50	0.27	0.79	0.17	0.30	0.90	0.22	0.94	0.97	0.33	0.20	0.23	0.04	0.51
2		--	0.76	0.75	0.67	0.94	0.51	0.17	0.21	0.70	0.15	0.15	0.05	0.49	0.09	0.18	0.26	0.48	0.83	0.43	0.79	0.57	0.08	0.74	0.92	0.13	0.07	0.05	0.13	0.11	0.27	0.65	0.96	0.06	0.17	0.16	0.11	0.33	0.27	0.40	0.12	0.63	0.33	0.75
3a			--	0.22	0.66	0.84	0.30	0.50	0.12	0.38	1.00	0.64	0.65	0.60	0.76	0.64	0.85	0.48	0.61	0.66	0.12	0.70	0.08	0.40	0.71	0.55	0.79	0.12	0.60	0.16	0.43	0.36	0.67	0.22	0.32	0.87	0.39	0.93	0.85	0.42	0.87	0.07	0.46	0.15
3b				--	0.01	0.02	0.01	0.02	0.02	0.08	0.01	0.27	0.10	0.08	0.19	0.01	0.01	0.32	0.60	0.81	0.39	0.61	0.01	0.01	0.01	0.23	0.15	0.09	0.24	0.14	0.36	0.01	0.54	0.03	0.04	0.29	0.15	0.47	0.25	0.02	0.04	0.06	0.32	0.04
V1					--	0.22	0.62	0.58	0.47	0.99	0.01	0.86	0.05	0.01	0.73	0.90	0.00	0.96	0.63	0.07	0.05	0.08	0.07	0.01	0.57	0.58	0.01	0.62	0.82	0.86	0.87	0.67	0.45	0.01	0.05	0.76	0.89	0.74	0.82	0.77	0.07	0.54	0.96	0.87
V2d						--	0.77	0.72	0.64	0.99	0.69	0.77	0.78	0.16	0.74	0.94	0.32	0.85	0.62	0.66	0.13	0.94	0.09	0.97	0.81	0.57	0.92	0.61	0.73	0.77	0.70	0.46	0.69	0.12	0.17	0.88	0.94	0.90	0.88	0.90	0.15	0.71	0.99	0.98
V2v							--	0.00	0.19	0.43	0.87	0.74	0.75	0.79	0.54	0.81	0.13	0.65	0.42	0.46	0.21	0.93	0.07	0.61	0.70	0.38	0.97	0.42	0.68	0.18	0.68	0.45	0.74	0.24	0.22	0.44	0.45	0.14	0.33	0.59	0.06	0.01	0.56	0.06
V3A								--	0.56	0.37	0.90	0.88	0.90	0.98	0.62	0.81	0.33	0.42	0.52	0.55	0.11	0.90	0.08	0.37	0.49	0.48	0.95	0.52	0.84	0.13	0.31	0.33	0.39	0.20	0.33	0.79	0.31	0.46	0.57	0.27	0.07	0.24	0.40	0.03
V3d									--	0.25	0.88	0.76	0.77	0.94	0.67	0.90	0.07	0.34	0.55	0.59	0.08	0.88	0.10	0.20	0.74	0.50	0.93	0.55	0.71	0.12	0.21	0.26	0.58	0.50	0.59	0.89	0.31	0.29	0.40	0.28	0.05	0.17	0.37	0.12
V3v										--	0.62	0.78	0.79	0.40	0.57	0.81	0.16	0.68	0.45	0.49	0.50	0.99	0.07	0.10	0.77	0.41	0.99	0.45	0.72	0.89	0.56	0.74	0.85	0.01	0.11	0.51	0.82	0.42	0.52	0.62	0.03	0.38	0.93	0.65
V4v											--	0.22	0.23	0.26	0.40	0.74	0.09	0.39	0.23	0.28	0.06	0.86	0.35	0.72	0.75	0.19	0.36	0.23	0.19	0.60	0.44	0.21	0.58	0.37	0.15	0.95	0.69	0.79	0.81	0.82	0.31	0.07	0.65	0.82
FG1												--	0.53	0.94	0.13	0.87	0.15	0.52	0.11	0.11	0.01	0.91	0.00	0.34	0.29	0.10	0.32	0.10	0.88	0.20	0.13	0.00	0.26	0.10	0.08	0.46	0.67	0.30	0.37	0.55	0.21	0.99	0.51	0.49
FG2													--	0.95	0.25	0.13	0.02	0.18	0.20	0.22	0.01	0.99	0.00	0.34	0.25	0.19	0.47	0.20	0.78	0.21	0.14	0.00	0.36	0.06	0.08	0.47	0.69	0.30	0.37	0.56	0.98	0.01	0.53	0.50
37B														--	0.77	0.38	0.57	0.51	0.34	0.82	0.84	0.65	0.51	0.56	0.59	0.50	0.40	0.50	0.95	0.20	0.20	0.11	0.57	0.13	0.05	0.71	0.66	0.40	0.49	0.60	0.33	0.38	0.55	0.48
37L															--	0.73	0.01	0.34	0.88	0.85	0.30	0.92	0.03	0.61	0.87	0.25	0.34	0.36	0.83	0.23	0.97	0.01	0.60	0.09	0.22	0.89	0.45	0.97	0.94	0.48	0.86	0.03	0.36	0.62
37M																--	0.19	0.34	0.97	0.82	0.59	0.99	0.04	0.98	0.98	0.27	0.53	0.53	0.09	0.97	0.12	0.12	0.47	0.01	0.10	0.26	0.50	0.04	0.10	0.21	0.17	0.23	0.74	0.03
41																	--	0.23	0.02	0.04	0.03	0.17	0.09	0.03	0.51	0.01	0.02	0.01	0.13	0.15	0.18	0.26	0.12	0.05	0.19	0.27	0.16	0.27	0.27	0.14	0.00	0.05	0.21	0.12
42																		--	0.67	0.59	0.88	0.36	0.05	0.35	0.75	0.37	0.27	0.14	0.47	0.07	0.24	0.87	0.91	0.09	0.25	0.13	0.14	0.23	0.31	0.63	0.09	0.05	0.32	0.87
20																			--	0.39	0.04	0.85	0.04	0.38	0.80	0.20	0.74	0.66	0.41	0.14	0.40	0.00	0.48	0.14	0.46	0.67	0.34	0.63	0.79	0.34	0.67	0.09	0.25	0.44
21																				--	0.07	0.86	0.30	0.45	0.87	0.32	0.56	0.64	0.50	0.16	0.55	0.01	0.66	0.15	0.33	0.74	0.37	0.74	0.88	0.39	0.83	0.05	0.28	0.49
22																					--	0.66	0.06	0.21	0.99	0.03	0.02	0.03	0.01	0.06	0.20	0.06	0.68	0.12	0.14	0.11	0.12	0.12	0.14	0.47	0.03	0.20	0.05	0.67
36																						--	0.24	0.85	0.49	0.82	0.99	0.76	0.91	0.29	0.49	0.24	0.83	0.10	0.29	0.83	0.88	0.44	0.66	0.95	0.15	0.61	0.55	0.84
38																							--	0.07	0.12	0.02	0.01	0.04	0.00	0.05	0.07	0.02	0.11	0.12	0.08	0.08	0.05	0.08	0.07	0.06	0.64	0.06	0.05	0.06
5L																								--	0.84	0.32	0.50	0.35	0.30	0.42	0.77	0.56	0.90	0.10	0.18	0.55	0.70	0.49	0.59	0.90	0.44	0.90	0.66	0.96
5M																									--	0.74	0.34	0.36	0.27	0.19	0.50	0.24	0.71	0.50	0.20	0.91	0.44	0.92	0.93	0.54	0.44	0.13	0.40	0.72
PGa																										--	0.13	0.34	0.49	0.12	0.29	0.00	0.31	0.10	0.76	0.59	0.30	0.52	0.68	0.30	0.51	0.79	0.22	0.38
PGp																											--	0.27	0.08	0.39	0.19	0.00	0.48	0.08	0.10	0.60	0.97	0.40	0.47	0.82	0.28	0.01	0.86	0.69
PFt																												--	0.75	0.14	0.39	0.57	0.73	0.16	0.47	0.66	0.33	0.62	0.78	0.34	0.66	0.09	0.25	0.44
PFm																													--	0.17	0.12	0.00	0.24	0.09	0.08	0.43	0.60	0.27	0.34	0.49	0.20	0.39	0.44	0.44
24																														--	0.03	0.40	0.49	0.05	0.14	0.03	0.27	0.10	0.10	0.15	0.06	0.01	0.87	0.25
32																															--	0.38	0.78	0.16	0.24	0.85	0.01	0.32	0.36	0.10	0.66	0.60	0.08	0.30
23																																--	0.17	0.10	0.20	0.31	0.67	0.35	0.37	0.65	0.10	0.03	0.45	0.68
31																																	--	0.42	0.44	0.97	0.62	0.99	0.99	0.69	0.47	0.38	0.67	0.83
4																																		--	0.73	0.08	0.10	0.13	0.06	0.13	0.29	0.10	0.10	0.17
6																																			--	0.31	0.15	0.30	0.28	0.18	0.29	0.33	0.14	0.18
8																																				--	0.02	0.92	0.82	0.12	0.13	0.48	0.13	0.29
9																																					--	0.15	0.15	0.21	0.09	0.28	0.92	0.56
10L																																						--	0.41	0.13	0.06	0.62	0.24	0.04
10M																																							--	0.11	0.08	0.01	0.26	0.12
11																																								--	0.91	0.08	0.49	0.90
44																																									--	0.24	0.11	0.63
45																																										--	0.01	0.05
46																																											--	0.01
47																																												--
