# Supplementary material for: Multiple Transmitter Receptors in Regions and Layers of the Human Cerebral Cortex
Source: Front Neuroanat. 2017 Sep 20;11:78. doi: 10.3389/fnana.2017.00078 (PMC5609104; doi:10.3389/fnana.2017.00078)
Supplement: Supplementary file 5 [file Table_5.pdf]

Supplementary Table 5:

P values resulting from the discriminant analyses of fingerprints obtained from the granular stratum of each area. Red codes p ≤ 0.01, orange codes p ≤ 0.05.

	1	2	3a	3b	V1	V2d	V2v	V3A	V3d	V3v	V4v	FG1	FG2	37B	37L	37M	41	42	20	21	22	36	38	5L	5M	PGa	PGp	PFt	PFm	32	23	31	4	6	8	9	10L	10M	11	44	45	46	47	
1	--	0.31	0.79	0.96	0.72	0.66	0.22	0.57	0.66	0.29	0.22	0.12	0.09	0.30	0.43	0.39	0.28	0.57	0.51	0.41	0.21	0.14	0.26	0.55	0.81	0.93	0.11	0.45	0.10	0.23	0.45	0.68	0.11	0.92	0.41	0.16	0.38	0.35	0.25	0.32	0.84	0.09	0.17	1
2		--	0.32	0.04	0.09	0.45	0.04	0.36	0.08	0.41	0.80	0.50	0.19	0.86	0.33	0.45	0.09	0.36	0.41	0.71	0.83	0.12	0.12	0.88	0.32	0.30	0.31	0.21	0.54	0.82	0.08	0.77	0.07	0.31	0.25	0.02	0.64	0.66	0.38	0.20	0.51	0.10	0.38	2
3a			--	0.53	0.69	0.78	0.52	0.93	0.95	0.28	0.54	0.53	0.40	0.11	0.82	0.55	0.46	0.11	0.77	0.84	0.11	0.46	0.17	0.49	0.84	0.64	0.48	0.30	0.46	0.05	0.29	0.77	0.12	0.51	0.48	0.19	0.03	0.19	0.02	0.04	0.18	0.28	0.21	3a
3b				--	0.05	0.04	0.05	0.09	0.08	0.06	0.02	0.00	0.00	0.02	0.00	0.01	0.01	0.05	0.00	0.00	0.01	0.07	0.01	0.00	0.01	0.00	0.00	0.01	0.00	0.01	0.01	0.43	0.05	0.05	0.00	0.01	0.04	0.03	0.00	0.05	0.00	0.16	0.00	3b
V1					--	0.02	0.08	0.04	0.16	0.03	0.15	0.15	0.13	0.04	0.01	0.90	0.04	0.01	0.01	0.08	0.05	0.07	0.26	0.02	0.69	0.01	0.04	0.02	0.32	0.11	0.01	0.14	0.07	0.12	0.02	0.31	0.82	0.89	0.01	0.02	0.02	0.02	0.02	V1
V2d						--	0.68	0.59	0.61	0.81	0.94	0.46	0.34	0.16	0.73	0.69	0.88	0.99	0.67	0.75	0.12	0.75	0.07	0.81	0.91	0.54	0.42	0.67	0.40	1.00	0.42	0.35	0.19	0.57	0.98	0.77	0.96	0.98	0.73	0.06	0.18	0.76	0.85	V2d
V2v							--	0.51	0.51	0.89	0.23	0.12	0.07	0.24	0.13	0.39	0.26	0.91	0.11	0.14	0.14	0.16	0.04	0.90	0.28	0.08	0.10	0.11	0.09	0.68	0.44	0.45	0.07	0.30	0.81	0.66	0.88	0.95	0.70	0.11	0.12	0.65	0.82	V2v
V3A								--	0.98	0.23	0.51	0.54	0.38	0.68	0.58	0.84	0.49	0.33	0.54	0.60	0.09	0.27	0.17	0.33	0.10	0.43	0.48	0.53	0.45	0.01	0.21	0.46	0.06	0.66	0.54	0.22	0.08	0.23	0.06	0.13	0.16	0.30	0.24	V3A
V3d									--	0.18	0.50	0.54	0.41	0.57	0.75	0.76	0.35	0.49	0.70	0.77	0.05	0.22	0.17	0.18	0.23	0.58	0.49	0.70	0.47	0.07	0.14	0.56	0.09	0.19	0.67	0.35	0.21	0.38	0.20	0.14	0.12	0.41	0.37	V3d
V3v										--	0.75	0.30	0.19	0.79	0.33	0.66	0.17	0.80	0.29	0.35	0.04	0.69	0.08	0.27	0.69	0.22	0.25	0.29	0.23	0.74	0.09	0.26	0.11	0.21	0.60	0.73	0.91	0.91	0.75	0.07	0.07	0.75	0.90	V3v
V4v											--	0.03	0.02	0.28	0.07	0.07	0.28	0.93	0.06	0.08	0.25	0.93	0.11	0.77	0.86	0.04	0.03	0.06	0.02	0.93	0.82	0.74	0.15	0.10	0.83	0.58	0.99	0.99	0.61	0.16	0.11	0.50	0.69	V4v
FG1												--	0.31	0.94	0.08	0.50	0.18	0.55	0.07	0.08	0.06	0.90	0.03	0.41	0.47	0.06	0.80	0.07	0.57	0.19	0.23	0.03	0.03	0.27	0.57	0.82	0.41	0.51	0.89	0.03	0.39	0.67	0.63	FG1
FG2													--	0.86	0.03	0.64	0.07	0.20	0.03	0.03	0.05	0.98	0.02	0.29	0.34	0.03	0.15	0.03	0.19	0.13	0.16	0.04	0.02	0.12	0.44	0.52	0.29	0.39	0.58	0.34	0.03	0.38	0.40	FG2
37B														--	0.78	0.72	0.08	0.72	0.44	0.81	0.73	0.51	0.67	0.09	0.37	0.12	0.51	0.48	0.18	0.13	0.42	0.37	0.12	0.31	0.71	0.59	0.34	0.51	0.49	0.03	0.28	0.55	0.52	37B
37L															--	0.78	0.06	0.33	0.78	0.83	0.95	0.85	0.05	0.61	0.92	0.33	0.28	0.61	0.72	0.42	0.74	0.05	0.03	0.19	0.78	0.37	0.73	0.78	0.37	0.96	0.15	0.30	0.40	37L
37M																--	0.08	0.53	0.45	0.96	0.15	0.76	0.06	0.64	0.34	0.15	0.56	0.49	0.46	0.09	0.45	0.81	0.07	0.15	0.35	0.99	0.07	0.36	0.62	0.40	0.22	0.72	0.82	37M
41																	--	0.12	0.07	0.11	0.17	0.04	0.03	0.06	0.15	0.05	0.11	0.24	0.20	0.14	0.24	0.19	0.10	0.03	0.04	0.03	0.05	0.10	0.08	0.13	0.06	0.01	0.03	41
42																		--	0.42	0.75	0.56	0.25	0.19	0.99	0.84	0.29	0.34	0.26	0.53	0.49	0.71	0.88	0.04	0.50	0.35	0.26	0.78	0.35	0.33	0.09	0.06	0.35	0.12	42
20																			--	0.38	0.64	0.84	0.05	0.55	0.97	0.07	0.94	0.67	0.17	0.35	0.63	0.05	0.02	0.22	0.73	0.34	0.66	0.72	0.34	0.39	0.18	0.28	0.37	20
21																				--	0.80	0.86	0.06	0.63	0.99	0.05	0.72	0.65	0.67	0.45	0.78	0.10	0.02	0.31	0.79	0.38	0.75	0.79	0.37	0.75	0.14	0.31	0.41	21
22																					--	0.53	0.06	0.04	0.56	0.22	0.06	0.08	0.05	0.35	0.44	0.15	0.03	0.22	0.85	0.15	0.54	0.44	0.21	0.05	0.44	0.15	0.20	22
36																						--	0.19	0.29	0.10	0.78	0.95	0.83	0.85	0.58	0.17	0.70	0.00	0.64	0.63	0.94	0.10	0.46	0.89	0.16	0.37	0.97	0.58	36
38																							--	0.10	0.11	0.03	0.02	0.04	0.02	0.10	0.07	0.06	0.05	0.30	0.12	0.08	0.09	0.10	0.08	0.05	0.11	0.08	0.08	38
5L																								--	0.91	0.43	0.36	0.62	0.34	0.38	0.12	0.85	0.03	0.40	1.00	0.54	0.55	0.73	0.47	0.43	0.33	0.61	0.63	5L
5M																									--	0.88	0.42	0.72	0.42	0.58	0.98	0.38	0.22	0.28	0.15	0.42	0.56	0.65	0.59	0.28	0.36	0.62	0.34	5M
PGa																										--	0.09	0.19	0.51	0.23	0.41	0.03	0.03	0.19	0.61	0.29	0.51	0.59	0.29	0.12	0.51	0.23	0.30	PGa
PGp																											--	0.30	0.09	0.16	0.20	0.03	0.03	0.19	0.52	0.70	0.36	0.47	0.78	0.03	0.04	0.55	0.53	PGp
PFt																												--	0.69	0.35	0.68	0.78	0.14	0.26	0.73	0.34	0.66	0.72	0.34	0.07	0.18	0.28	0.37	PFt
PFm																													--	0.15	0.19	0.02	0.03	0.33	0.50	0.65	0.34	0.44	0.72	0.03	0.06	0.50	0.49	PFm
32																														--	0.40	0.94	0.02	0.38	0.61	0.19	0.24	0.48	0.09	0.14	0.87	0.26	0.26	32
23																															--	0.77	0.01	0.34	0.79	0.53	0.97	0.98	0.57	0.06	0.05	0.51	0.63	23
31																																--	0.05	0.08	0.87	0.73	0.97	0.95	0.68	0.04	0.21	0.72	0.76	31
4																																	--	0.29	0.03	0.02	0.02	0.08	0.02	0.14	0.06	0.02	0.02	4
6																																		--	0.45	0.21	0.08	0.27	0.04	0.02	0.02	0.28	0.21	6
8																																			--	0.02	0.51	0.28	0.24	0.10	0.10	0.19	0.04	8
9																																				--	0.37	0.20	0.97	0.07	0.09	0.74	0.06	9
10L																																					--	0.99	0.02	0.08	0.90	0.52	0.53	10L
10M																																						--	0.29	0.08	0.20	0.45	0.33	10M
11																																							--	0.14	0.11	0.88	0.68	11
44																																								--	0.22	0.07	0.70	44
45																																									--	0.02	0.07	45
46																																										--	0.01	46
47																																											--	47
