# Supplementary material for: Multiple Transmitter Receptors in Regions and Layers of the Human Cerebral Cortex
Source: Front Neuroanat. 2017 Sep 20;11:78. doi: 10.3389/fnana.2017.00078 (PMC5609104; doi:10.3389/fnana.2017.00078)
Supplement: Supplementary file 6 [file Table_6.pdf]

Supplementary Table 6:

P values resulting from the discriminant analyses of fingerprints obtained from the infragranular stratum of each area. Red codes p ≤ 0.01, orange codes p ≤ 0.05.

	1	2	3a	3b	V1	V2d	V2v	V3A	V3d	V3v	V4v	FG1	FG2	37B	37L	37M	41	42	20	21	22	36	38	5L	5M	PGa	PGp	PFt	PFm	24	32	23	31	4	6	8	9	10L	10M	11	44	45	46	47	
1	--	0.69	0.72	0.78	0.78	0.65	0.25	0.31	0.52	0.76	0.84	0.06	0.05	0.14	0.24	0.39	0.08	0.77	0.36	0.40	0.31	0.09	0.24	0.39	0.16	0.85	0.05	0.59	0.04	0.04	0.16	0.38	0.65	0.73	0.67	0.55	0.18	0.48	0.47	0.25	0.34	0.06	0.20	0.19	1
2		--	0.13	0.64	0.31	0.42	0.27	0.13	0.48	0.43	0.90	0.22	0.08	0.49	0.11	0.64	0.10	0.55	0.12	0.21	0.35	0.23	0.22	0.83	0.65	0.13	0.11	0.11	0.19	0.03	0.16	0.68	0.75	0.33	0.13	0.15	0.09	0.44	0.39	0.19	0.08	0.80	0.03	0.17	2
3a			--	0.35	0.55	0.89	0.50	0.33	0.26	0.32	0.71	0.46	0.41	0.71	0.99	0.30	0.62	0.53	0.91	0.89	0.09	0.70	0.35	0.56	0.50	0.80	0.39	0.37	0.33	0.09	0.01	0.23	0.73	0.86	0.26	0.32	0.21	0.09	0.13	0.04	0.36	0.33	0.23	0.24	3a
3b				--	0.24	0.23	0.55	0.40	0.04	0.64	0.01	0.27	0.13	0.68	0.17	0.01	0.01	0.40	0.19	0.26	0.19	0.34	0.01	0.00	0.01	0.19	0.17	0.40	0.24	0.17	0.09	0.00	0.79	0.01	0.01	0.33	0.17	0.19	0.18	0.10	0.16	0.14	0.19	0.20	3b
V1					--	0.01	0.55	0.22	0.20	0.37	0.05	0.39	0.04	0.00	0.90	0.52	0.78	0.03	1.00	0.02	0.04	0.01	0.30	0.01	0.47	0.88	0.01	0.90	0.29	0.09	0.01	0.25	0.53	0.10	0.01	0.31	0.20	0.09	0.13	0.04	0.03	0.01	0.22	0.23	V1
V2d						--	0.85	0.33	0.57	0.75	0.39	0.53	0.48	0.22	0.87	0.70	0.34	0.53	0.79	0.78	0.05	0.21	0.06	0.78	0.57	0.69	0.45	0.87	0.37	0.54	0.24	0.10	0.53	0.79	0.17	0.90	0.66	0.62	0.69	0.66	0.51	0.47	0.69	0.71	V2d
V2v							--	0.34	0.35	0.97	0.19	0.13	0.11	0.16	0.31	0.25	0.56	0.85	0.23	0.22	0.20	0.25	0.09	0.73	0.40	0.17	0.10	0.31	0.08	0.23	0.55	0.17	0.47	0.30	0.24	0.91	0.33	0.57	0.57	0.40	0.15	0.05	0.35	0.37	V2v
V3A								--	0.59	0.17	0.27	0.56	0.43	0.78	0.32	0.92	0.49	0.38	0.28	0.27	0.09	0.64	0.46	0.33	0.51	0.23	0.38	0.32	0.26	0.07	0.01	0.17	0.24	0.27	0.18	0.16	0.11	0.01	0.04	0.01	0.04	0.86	0.12	0.14	V3A
V3d									--	0.13	0.44	0.50	0.45	0.66	0.90	0.66	0.23	0.20	0.82	0.80	0.05	0.41	0.55	0.41	0.74	0.71	0.42	0.90	0.35	0.09	0.02	0.16	0.52	0.31	0.07	0.32	0.22	0.12	0.17	0.07	0.06	0.41	0.25	0.25	V3d
V3v										--	0.88	0.46	0.40	0.20	0.83	0.64	0.83	0.57	0.74	0.72	0.08	0.27	0.12	0.93	0.48	0.63	0.38	0.84	0.31	0.31	0.20	0.33	0.73	0.41	0.43	0.92	0.48	0.46	0.53	0.37	0.01	0.57	0.51	0.54	V3v
V4v											--	0.19	0.17	0.12	0.75	0.27	0.09	0.77	0.95	0.99	0.42	0.46	0.01	0.54	0.62	0.77	0.16	0.74	0.13	0.23	0.56	0.15	0.91	0.77	0.44	0.73	0.35	0.57	0.56	0.40	0.90	0.52	0.38	0.39	V4v
FG1												--	0.10	0.86	0.11	0.21	0.15	0.71	0.10	0.10	0.09	0.87	0.16	0.41	0.55	0.09	0.63	0.11	0.27	0.56	0.11	0.15	0.60	0.25	0.10	0.59	0.93	0.49	0.60	1.00	0.02	0.34	0.96	0.87	FG1
FG2													--	0.93	0.17	0.96	0.07	0.43	0.16	0.15	0.07	0.97	0.13	0.34	0.47	0.14	0.78	0.17	0.73	0.26	0.09	0.13	0.55	0.18	0.09	0.53	0.90	0.43	0.54	0.81	0.94	0.01	0.81	0.71	FG2
37B														--	0.79	0.46	0.51	0.76	0.30	0.82	0.69	0.69	0.39	0.21	0.43	0.66	0.53	0.54	0.22	0.14	0.06	0.47	0.10	0.48	0.06	0.62	0.57	0.34	0.47	0.33	0.01	0.24	0.59	0.55	37B
37L															--	0.81	0.10	0.42	0.75	0.82	0.99	0.84	0.28	0.46	0.97	0.15	0.05	0.67	0.85	0.08	0.29	0.65	0.91	0.25	0.28	0.83	0.32	0.82	0.82	0.31	0.14	0.02	0.38	0.36	37L
37M																--	0.07	0.62	0.41	0.90	0.07	0.79	0.10	0.91	0.69	0.65	0.71	0.39	0.37	0.55	0.03	0.20	0.90	0.13	0.08	0.24	0.97	0.12	0.02	0.94	0.49	0.65	0.89	0.79	37M
41																	--	0.09	0.11	0.15	0.30	0.11	0.32	0.69	0.28	0.11	0.09	0.20	0.13	0.08	0.21	0.18	0.82	0.50	0.05	0.22	0.08	0.16	0.15	0.13	0.14	0.21	0.09	0.09	41
42																		--	0.43	0.60	0.30	0.43	0.01	0.09	0.90	0.40	0.58	0.24	0.47	0.32	0.89	0.03	0.91	0.33	0.60	0.19	0.21	0.38	0.24	0.06	0.26	0.21	0.34	0.11	42
20																			--	0.23	0.54	0.82	0.21	0.39	0.97	0.16	0.84	0.73	0.23	0.07	0.21	0.46	0.43	0.26	0.35	0.77	0.29	0.73	0.76	0.28	0.87	0.03	0.34	0.33	20
21																				--	0.49	0.81	0.21	0.38	0.98	0.18	0.83	0.70	0.61	0.07	0.20	0.43	0.51	0.32	0.37	0.76	0.29	0.72	0.74	0.28	0.69	0.03	0.34	0.32	21
22																					--	0.59	0.04	0.13	0.21	0.28	0.07	0.14	0.06	0.13	0.47	0.70	0.24	0.20	0.07	0.88	0.14	0.31	0.29	0.18	0.01	0.22	0.15	0.16	22
36																						--	0.15	0.26	0.21	0.79	0.92	0.81	0.82	0.69	0.09	0.17	0.63	0.14	0.29	0.70	0.61	0.16	0.51	0.85	0.39	0.28	0.51	0.84	36
38																							--	0.01	0.17	0.16	0.12	0.28	0.09	0.13	0.09	0.02	0.15	0.10	0.08	0.22	0.14	0.14	0.15	0.12	0.02	0.01	0.14	0.15	38
5L																								--	0.84	0.32	0.31	0.55	0.23	0.59	0.39	0.04	0.85	0.35	0.21	0.97	0.65	0.79	0.86	0.70	0.18	0.22	0.71	0.75	5L
5M																									--	0.93	0.46	0.74	0.39	0.57	0.37	0.89	0.64	0.65	0.45	0.44	0.31	0.84	0.63	0.59	0.45	0.35	0.61	0.56	5M
PGa																										--	0.09	0.21	0.44	0.06	0.15	0.31	0.86	0.27	0.52	0.68	0.26	0.63	0.67	0.25	0.11	0.06	0.31	0.29	PGa
PGp																											--	0.23	0.55	0.20	0.08	0.12	0.53	0.20	0.09	0.51	0.82	0.40	0.51	0.73	0.01	0.01	0.74	0.65	PGp
PFt																												--	0.74	0.08	0.30	0.46	0.54	0.36	0.28	0.84	0.32	0.82	0.83	0.31	0.14	0.02	0.38	0.36	PFt
PFm																													--	0.11	0.06	0.10	0.42	0.22	0.07	0.42	0.57	0.33	0.42	0.50	0.01	0.46	0.53	0.46	PFm
24																														--	0.09	0.22	0.37	0.20	0.11	0.08	0.82	0.25	0.23	0.72	0.02	0.01	0.34	0.18	24
32																															--	0.39	0.97	0.12	0.13	0.40	0.08	0.06	0.08	0.01	0.99	0.60	0.09	0.10	32
23																																--	0.99	0.16	0.33	0.82	0.38	0.98	0.95	0.47	0.31	0.18	0.43	0.45	23
31																																	--	0.53	0.35	0.98	0.71	0.94	0.91	0.67	0.82	0.19	0.70	0.75	31
4																																		--	0.66	0.25	0.21	0.24	0.21	0.14	0.89	0.67	0.21	0.20	4
6																																			--	0.21	0.11	0.13	0.13	0.10	0.34	0.36	0.12	0.13	6
8																																				--	0.11	0.46	0.36	0.20	0.02	0.82	0.16	0.19	8
9																																					--	0.41	0.44	0.99	0.02	0.28	0.15	0.18	9
10L																																						--	0.78	0.13	0.02	0.86	0.48	0.55	10L
10M																																							--	0.24	0.02	0.01	0.54	0.63	10M
11																																								--	0.19	0.04	0.95	0.84	11
44																																									--	0.03	0.02	0.23	44
45																																										--	0.01	0.02	45
46																																											--	0.01	46
47																																												--	47
