# Supplementary material for: Multiple Transmitter Receptors in Regions and Layers of the Human Cerebral Cortex
Source: Front Neuroanat. 2017 Sep 20;11:78. doi: 10.3389/fnana.2017.00078 (PMC5609104; doi:10.3389/fnana.2017.00078)

Supplementary Figure 2: Normalized multi-receptor fingerprints of 15 different receptor types in each cortical area. The fingerprints are from additional cortical areas not shown in Figure 5. The fingerprints are normalized by their z-scores, and the dotted line indicates the average z-score over all areas. For further information, see Figure 7.

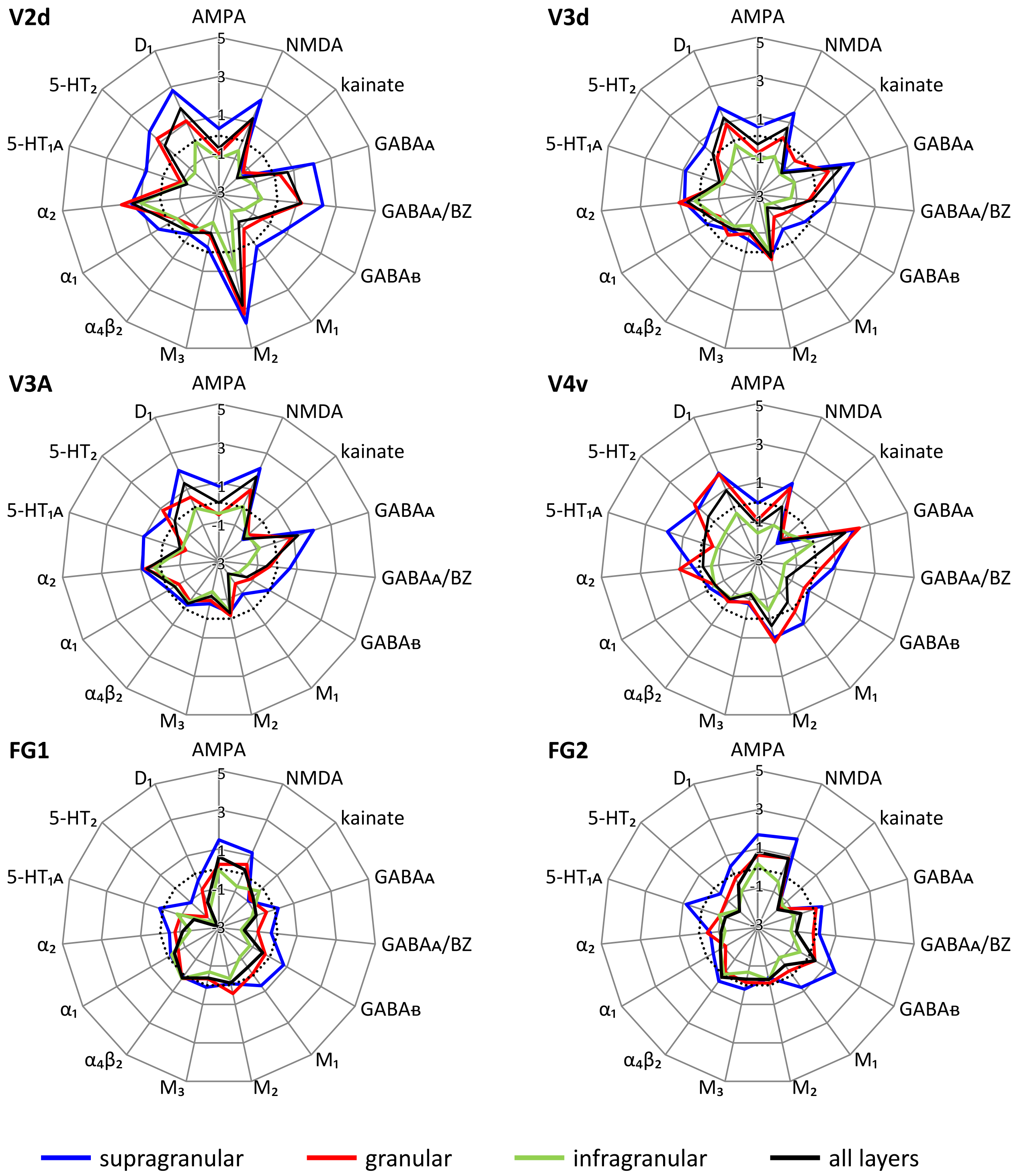

Supplementary Figure 2 (continued - 1)

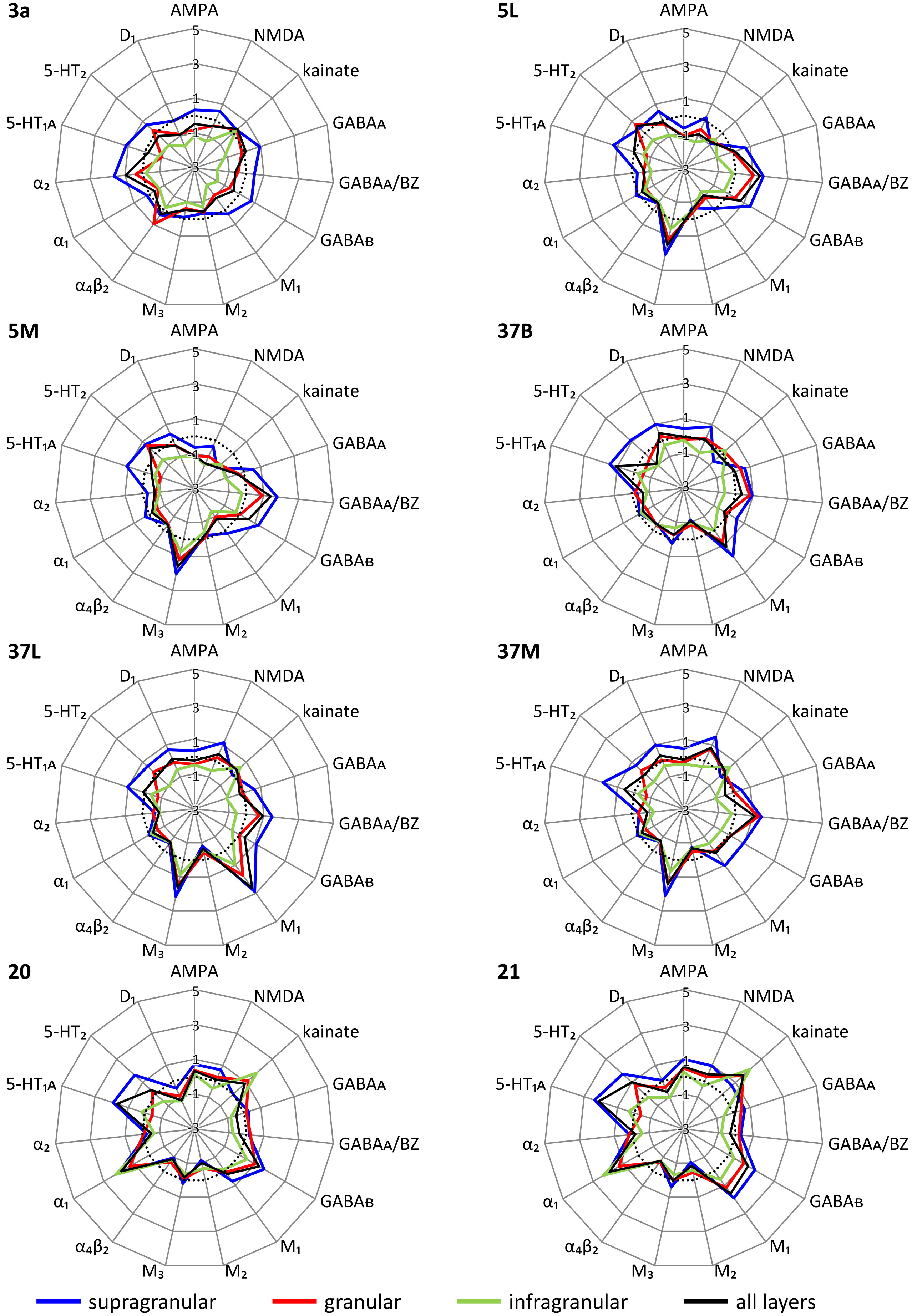

Supplementary Figure 2 (continued - 2)

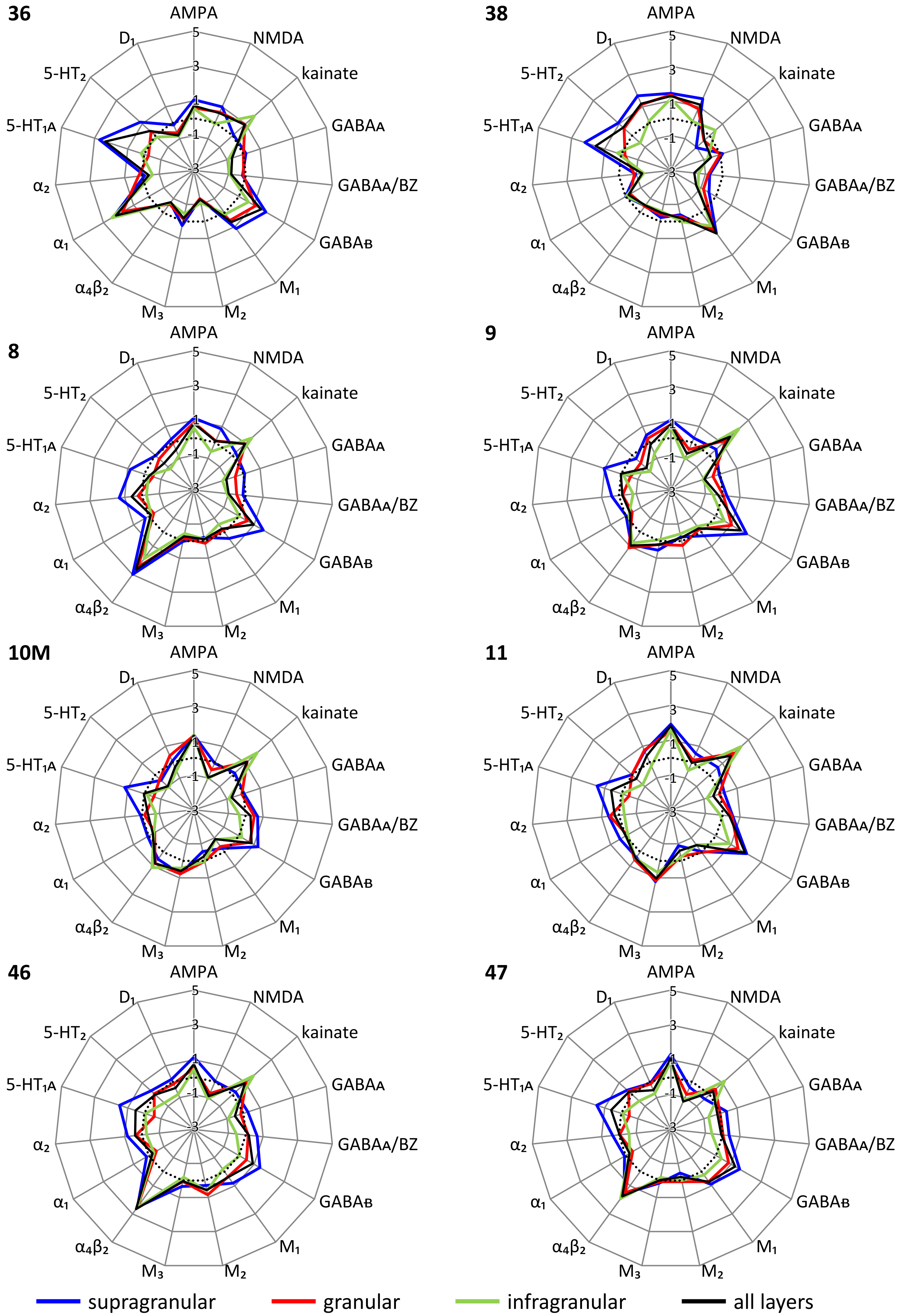

Supplementary Figure 2 (continued - 3)

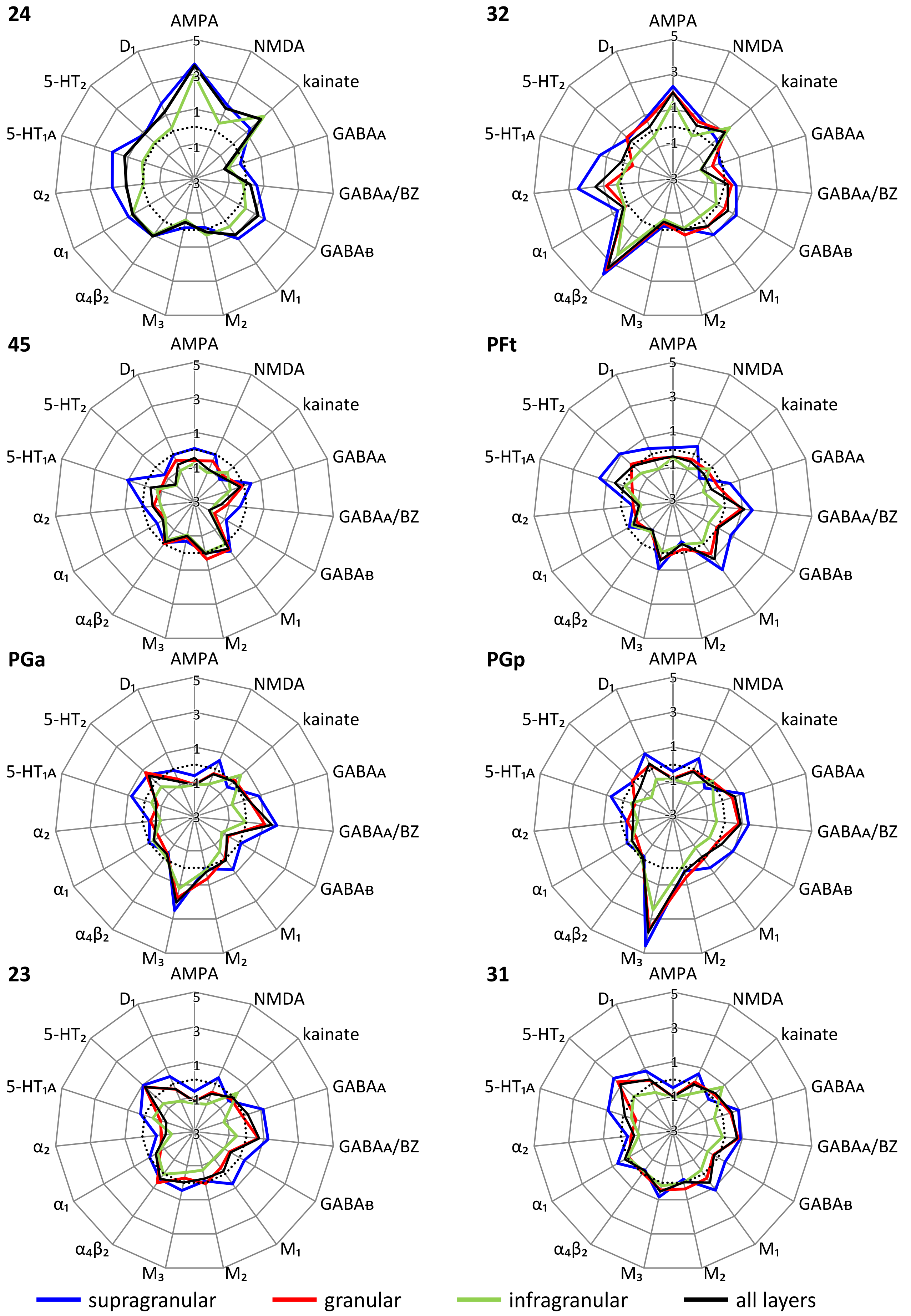

Supplement: Supplementary file 8 [file Image_2.pdf]
